# Supplementary material for: Identification and temporal expression of putative circadian clock transcripts in the amphipod crustacean Talitrus saltator
Source: PeerJ. 2016 Oct 5;4:e2555. doi: 10.7717/peerj.2555 (PMC5068443; doi:10.7717/peerj.2555)
Supplement: Figure S1 — Alignment of Danaus plexippus CRY2 (Danpl-CRY2; Accession No. ABA62409) with the T. saltator CRY2 (Tal-CRY2) deduced from homology cloning, together with the top two tblastn species homologue sequences Eurydice pulchra CRY2 (Ep-CRY2; Accession No. KC885970) and Bombus terrestris cryptochrome-1-like (Bomte-CRY1; Accession No. XM_012312885). ’*’ indicates identical amino acid residues in the two proteins, ’.’ and ’:’ indicate similar amino acid residues between the two proteins. In this figure SMART identified domains of one DNA photolyase domain and one FAD binding 7 domain are highlighted in yellow and green respectively. [file peerj-04-2555-s001.pdf]

```
Danpl-CRY2      ----------------------------MSVAETLPLRARSPTAQKSSQPAGVPKEKHTV
Tal-CRY2        MSPNKIASPGERSIHRTILGDNNLTFSESGSSSGLAFTAEKKNTSKVVSG------KHVV
Ep-CRY2         ----------------------------------MKMTG--TNGSGKAKP------KNVV
Bomte-CRY1      ----------------------------MTGSRSSEINPDVTVRGEGG--------KHTV
                                                    :                   *:.*

Danpl-CRY2      HWFRKGLRLHDNPALREGLVDATTFRCVFIIDPWFASSSNVGINKWRFLLQCLEDLDKNL
Tal-CRY2        HWFRRGLRLHDNPALRDAIVNCETFRCIYILDPWFAGSSNVGVNKWRFLLQCLEDVDNSL
Ep-CRY2         HWFRKGLRIHDNPALRLGLRNCTTFRCIFILDPWFAGSSNVGVNRWRFLLQSLEDLDRSL
Bomte-CRY1      HWFRKGLRLHDNPSLREGLTGATTFRCVFVLDPWFAGSTNVGINKWRFLLQCLEDLDCSL
                ****:***:****:** .: .. ****::::*****.*:***:*:******.***:* .*

Danpl-CRY2      RKLNSRLFVVRGQPADALPKLFREWGTTALTFEEDPEPYGRVRDHNIMTKCREVGIQVTS
Tal-CRY2        RNLNSRLFVVRGQPANALPQLFKEWNTTVLSFEEDPEPFGRARDASIIGIAQEMGIEVIV
Ep-CRY2         RTFNSRLFVIRGQPAKALPELFKEWDTTILTFEEDPEPFGKARDANVVAMAKEMGIKVIV
Bomte-CRY1      RKLNSRLFVIRGQPADALPKLFKEWGTTNLTFEEDPEPFGRVRDHNISALCKELGISVVQ
                *.:******:*****.***:**:**.** *:*******:*:.** .:   .:*:**.*

Danpl-CRY2      RVSHTLYKLDDIIEKNGGKAPLTYHQFQALIASMPPPPSAEPTISLETLNRAVTPISDNH
Tal-CRY2        RTSHTLYELDKIIKKKGGKPPLTYKTFQNILAMMDPPPPPVAPIEASDLKHAYTPLQHDH
Ep-CRY2         RTSHTLYKLDRIIETNGGKPPLTYKSFQNILSHIGPPSKPDSPVNREEINGGITPVSDDH
Bomte-CRY1      KVSHTLYKLDEIIERNGGKPPLTYHQFQNVVASMDPPEPSVSTVTSACIGSAYTPLKEDH
                :.*****:** **: :***.****: ** ::: : **  . ..:    :  . **:..:*

Danpl-CRY2      DERFGVPTLEELGFDTEGLKPPIWIGGENEALLRLERHLERKAWVASFGRPKMTPESLLS
Tal-CRY2        DDKYGVPNLEHLGFETEHLPPAVWKGGETEALSRLKHHLERKAWVASFGRPKMTPQSLFA
Ep-CRY2         DDKFGVPSLEDLGFDTDHLPNPVWKGGETEALTRLEHHLERKAWVASFGRPKMTPQSLYA
Bomte-CRY1      DDHYGVPTLEELGFDTEGLLPPVWVGGESEALARLERHLERKAWVASFGRPKMTPQSLLP
                *:::***.**.***:*: *  .:* ***.*** **::******************:** .

Danpl-CRY2      SQTGLSPYLRFGCLSTRLFYYQLSELYKRIKQERPPLSLHGQILWREFFYCAATRNPNFD
Tal-CRY2        CPTGLSPYLRFGCLSARKFYTELNVLYTKIKKVPAPVSLHGHLLWREFFYTAATNNPKFD
Ep-CRY2         SPTGLSPYLRFGCLSPRQFYVKLNELFQKIKKLPPPLSIHGQLLWREFYYCAATNNPKFD
Bomte-CRY1      SQTGLSPYLRFGCLSTRLFYYQLTDLYKKIKKAVPPLSLHGQLLWREFFYCAATKNPNFD
                . *************.* ** :*. *: :**:  .*:*:**::*****:* ***.**:**

Danpl-CRY2      RMEGNPICVQIPWEKNQEALKKWANGQTGFPWIDAIMIQLRNDGWIHHLARHAVACFLTR
Tal-CRY2        HMKGNPICVQIPWDKNPEALAKWAHGQTGFPWIDAIMTQLRTEGWIHNVARHAVACFLTR
Ep-CRY2         HMEGNPICVQIPWDKNPEALAKWANGQTGYPWIDAIMTQLRQEGWVHNVARHAVACFLTR
Bomte-CRY1      RMQGNPICVQIPWDKNVEALAKWANGQTGFPWIDAIMTQLREEGWIHHLARHAVACFLTR
                :*:**********:** *** ***:****:******* *** :**:*::***********

Danpl-CRY2      GDLWISWEEGMKVFDELLLDADWSVNAGMWMWLSCSSFFQQFFHCYCPVRFGRKTDPNGD
Tal-CRY2        GNLWVSWEEGMKVFDELLLDADWSVNAGSWMWLSCSSFFQQFFHCYCPVRYGRKADPNGD
Ep-CRY2         GDLWVSWEEGMKVFDEPLLDADWAVNAGSWMWLSCSSFFQQFFHCYCPVRYGRKADPNGD
Bomte-CRY1      GDLWISWEEGMKVFDELLLDADWSVNAGMWMWLSCSSFFQQFFHCYCPVRFGRKADPNGD
                *:**:*********** ******:**** *********************:***:*****

Danpl-CRY2      FIRKYIPVLKNMPTRYIHEPWVCPEEIQKSIRCIIGKDYPMPIVDHTKASEINLERIKQV
Tal-CRY2        FIRTYLPVLKNFPTKYIHEPWMAPESVQRNARCIIGQHYPLPMVDHGTQSQNNIERMKQV
Ep-CRY2         YIRTYLPVLKNFPSRYIHEPWTAPEGVQRAAKCIIGIDYPMPMVDHSKQSQHNIERMKQV
Bomte-CRY1      YIRRYLPILKNFPTRYIHEPWNAPLSVQRAAKCIIGKDYSLPMVNHSKSSRINIERMKQV
                :** *:*:***:*::****** .*  :*:  :**** .*.:*:*:* . *. *:**:***

Danpl-CRY2      YAQLAKFKPQGALIPQMLQRPNVLQSSPSPTSIIANINQSNYLCSQSSDVPTPTNQTTNQ
Tal-CRY2        YQQLAHYR---ANISTRPCGDSKLRCKYPLH---STA-----------------------
Ep-CRY2         YQQLTHYRGTAPPFSKSMKNRNHINIEFPPP---RFEFEKPFPKKDKREVKTAVD-----
Bomte-CRY1      YQQLNKYRGNGASLKG--ETVGLLNALPPPS--VKEKEEEKKRTKQSP--PPPENQSK--
                * ** :::   . :       . :.   .

Danpl-CRY2      FKEDAVFLKPTVNNIKSNVDKQQQFKQVVIVQEDKHSENQRHSVGNKYIVNEINKNINDI
Tal-CRY2        ------------------------------------------------------------
Ep-CRY2         ------------------------------------------------------------
Bomte-CRY1      -----------MEALAKTTQHQQQHHQHQ-------------------------------
```

```
Danpl-CRY2       PVKQNNYDFKALTLNLNKFSNEPLTFLNQTPNKNESFGQDVNNVIDVYSTSKPKFYFTDN
Tal-CRY2         ------------------------------------------------------------
Ep-CRY2          ------------------------------------------------------------
Bomte-CRY1       ------------------------------------------------------------

Danpl-CRY2       GVITHNENAQTFKRDSYSDNYNKESTGSNRVGEVHSNNPQTDKISSEKKN
Tal-CRY2         --------------------------------------------------
Ep-CRY2          --------------------------------------------------
Bomte-CRY1       --------------------------------------------------
```

Figure S1. Putative *Talitrus saltator* CRYPTOCHROME 2 (CRY2) protein

Alignment of *Danaus plexippus* CRY2 (Danpl-CRY2; Accession No. ABA62409) with the *T. saltator* CRY2 (Tal-CRY2) deduced from homology cloning, together with the top two tblastn species homologue sequences *Eurydice pulchra* CRY2 (Ep-CRY2; Accession No. KC885970) and *Bombus terrestris* cryptochrome-1-like (Bomte-CRY1; Accession No. XM_012312885). '*' indicates identical amino acid residues in the two proteins, '.' and ':' indicate similar amino acid residues between the two proteins. In this figure SMART identified domains of one DNA photolyase domain and one FAD binding 7 domain are highlighted in yellow and green respectively.
