# Supplementary material for: Identification and temporal expression of putative circadian clock transcripts in the amphipod crustacean Talitrus saltator
Source: PeerJ. 2016 Oct 5;4:e2555. doi: 10.7717/peerj.2555 (PMC5068443; doi:10.7717/peerj.2555)
Supplement: Figure S2 — Alignment of Drosophila melanogaster PER (Drome-PER; Accession No. AAF45804) with the T. saltator PER (Tal-PER) deduced from the Trinity de novo transcriptome assembly, together with the top two tblastn species homologue sequences Eurydice pulchra PER (Ep-PER; Accession No. KC885967) and Periplaneta americana (Peram-PER; Accession No. U12772). ’*’ indicates identical amino acid residues in the two proteins, ’.’ and ’:’ indicate similar amino acid residues between the two proteins. In this figure SMART identified domains of two PAS domains, one PAC domain and one Period C domain are highlighted in yellow, green and blue respectively. The region of Ep-PER that corresponds to the PAC domain is shown in green text, though no PAC domain per se was identified in this protein using SMART. Drome-PER DBT/CK1ε binding site is highlighted in pink. Dark red text indicates Drome-PER CLK/CYC inhibitory domain (CCID). Pink text indicates regions in Tal-PER, Ep-PER and Peram-PER corresponding to sequence within both the overlapping Drome-PER DBT/CK1ε binding site and CCID regions. [file peerj-04-2555-s002.pdf]

```
Drome-PER       -----------------------------MEGGESTESTHNTKVSDS-------------
Tal-PER         MVLGDHRTSNGSSCTQEAMEVLPPKLEEAGNQSLPSGVPTDQHVSNSPPESQEDTAAASA
Ep-PER          ---------------------------------MDTGTPHEDVPSE--------------
Peram-PER       ---------------------------------MEETATHNTKISDS-------------
                                                      . :   *:

Drome-PER       --------------AYSNSCSNSQSQRS-GSSKSRLSGSHSSGSSGYGGK-PSTQASSSD
Tal-PER         IESDPEGRRQQPQNGQDPTNPCSQADQGYGSTESSFNG-QSRKSSSYSGGGSLTKSRNSG
Ep-PER          ---------DHTLEEGDSKNPSCQQESAYGSLESSSNG-QSQKS----FGGSGSKSLNSG
Peram-PER       --------------AYSNS-SNSQSQRSSGSSKSRHS--NSSGSSGYCGHGSSIQGSSNE
                                . . . .* : . ** :*  .  :*  *       .  :. ..

Drome-PER       -----MIIKRNKDKSRKKKKNKGAGQGAGQAQTLISASTSLEGRDEEKPRPSGTGCVEQQ
Tal-PER         SSQSSGFGEQHKKDAHTASITSSDKESATRAPHIDTQHSNDGNTEELTDNLAAISCKSKS
Ep-PER          SSHSSGFGDQNDFKG----------IHLHEAKHIALKKKKTGKGGEKVAEIPFQTASEAE
Peram-PER       PFPQPSVTKRNKDKEHKKKKLKSSVTTAATATVTSVVTTVSEYTEHENGTSHMSLGVSGT
                      . .::. .                *       .      .           .

Drome-PER       ICRELQDQQHGEDHSEPQAIEQLQQEEEEDQSGSESEADRVEGV----------------
Tal-PER         SSVGVDKLHDGAHYGSSTPREKRTKEQKLRDFKRKKVEESHYQTYDSFPRPPESYSFAAA
Ep-PER          LSS----------KGNETEKEKETSLEESPAAKEEAIIE---------------------
Peram-PER       VVPLSGTVAEETEITEAGSEGSVISSHAGVALGAAGVVPATTPG----------------
                               .     .  . .

Drome-PER       ------------------------------------------------------------
Tal-PER         AARASDLPDKHVENFTAPLISLQTQKDEFREPLRNFSGPSCSKNDYAIGSKATCLQSASM
Ep-PER          ------------------------------------------------------------
Peram-PER       ------------------------------------------------------------

Drome-PER       --------------------------AKSEAAQSFPIPSPLSVTIVPPSMGGCGGVGHAA
Tal-PER         PSASGVKRSLDSSVVPGAQQSFFSQCAASTSYTVSSSCRVKNEITDQPTLIYTQALNYIR
Ep-PER          ---------------------------KESRYIHPRNSFFEERHGSQTSLVYTQALNYIR
Peram-PER       --------------------------PENEAHQMASLTQTLNSIKKMKKMK---DLSTDI
                                            .            .      .:     :.

Drome-PER       GLDSGLAKFDKTWEAGPGKLESMTGVGAAAAGTGQRGERVKEDSFCCVISMHDGIVLYTT
Tal-PER         RIKEGFTNKGQVFQSPE--REHLSSNQSEAIADFIKSFGSNQRGFTMVLSIRDGTIIRVS
Ep-PER          KIKERSAEQGVPFASPY--LDQLSSNQ-EDITEFFKSFISS-RGFTVAISIQDGSVLQVS
Peram-PER       PEETEGHSFSLPMVAEE-KEEHIRNSFDAEPPAHNEGE------FCVVVSMQDGVVVFTT
                  .    . .    :     : : .          ..       *  .:*::** :: .:

Drome-PER       PSITDVLGYPRDMWLGRSFIDFVHLKDRATFASQITTGIPIAESRGS---VPKDAKSTFC
Tal-PER         TNISEVLGFPEDMVVGHSFIDFVYPRDSVHFSSKIVSGMSLFTKNSSTRAESLMP--PFY
Ep-PER          PTITDILGFPKDMLLGQSFIDFVYPKDSINFSSKIINGLNIPFRN-SAIKDTHGT--TFF
Peram-PER       PSITDVVGFPKDMWLGRSFIDFVHPRDRTAFANHITSGVITPLSNSNPKGGSHPGKNSFY
                ..*::::*:*.** :*:******: :*   *:.:* .*:     . .    .     .*

Drome-PER       VMLRRYRGLKSGGFGVIGRPVSYEPFRLGLTFREAP-------EEARPDNYMVSNGTN-M
Tal-PER         CRVRENKCYQASAFDMRGK-NSYKPCKITLKFNDTLT----PLEEEVMAPISPFHLPTSD
Ep-PER          CRLRMYHGLKNTGYGIRNKKTLYKPCKIIFKSQDAAR----FTDSKSVAHSS--------
Peram-PER       CCLRRYRGLKSTGYGVTEKEVSYLPFQLNMTFRELLPHSNPLEVEGNTSPESVPGGCNSM
                  :*  :  :  .:.:  :   * * :: :. .:          .

Drome-PER       LLVICATPIKSSYKVPDEILSQKSPKFAIRHTATGIISHVDSAAVSALGYLPQDLIGRSI
Tal-PER         VLLAEVIPVPSFYQVPDEVIN--GGNFVIRHSASCNFSEYDPDAIPFLGHLPQDLTGNSI
Ep-PER          LIIAEIIPIESAYKETDEATA--MGSFSIRHSASCNFSEYDPEAIPYLGHLPQDLTGNSI
Peram-PER       FLVITAKLICSAYKHAGETCA--SPKFVTRHLATCKLNYVDPECMPYLGYLPHEMLGNSV
                .::     : * *: ..*       .*  ** *:  :.  *. .:. **:**::: *.*:

Drome-PER       MDFYHHEDLSVMKETYETVMKKGQTAGASFCSKPYRFLIQNGCYVLLETEWTSFVNPWSR
Tal-PER         FDCYHPEDLPLLLTIYREIIREE---GKPFRSESYRFRTFNGSWVVLETEWLCFVNPWTR
Ep-PER          FDCYHPEDMSKLKNIYVEIVKHQ---GKPYRSEPYRFRTFNNSWVTLITEWLCFVNPWTK
Peram-PER       LDFYHPEDLPFLKEVYQIVMQEN---GAPFRSKPYRFRSHNGGYILLETEWSSFVNPWSK
                :* ** **:. :   *  :::.    * .: *:.***   *. :: * *** .*****::
```

```
Drome-PER       KLEFVVGHHRVFQGPKQCNVFEA-APTCKLKISEEAQSRNTRIKEDIVKRLAETVSR---
Tal-PER         KIDSIIGQHKVIKGPRDISIYMEQCEGSLSTYSEEVCIIARKARREIIDLLSRPVAASLA
Ep-PER          RIESIIGQHRILKVPKDINLFMDPSDKALSPLPEDILLEAQSAQKEILDLLMKPVTS---
Peram-PER       KLEFVVGQHRVLKGPENADVFMAPVEDDTLQISEEVLKESKIIQEEIRSLLSEMVKN---
                ::: ::*:*:::: *.: .::           .*:        :.:* . * . *

Drome-PER       ------------------PSDTVKQEVSRRCQALASFMETLMDEVSRADLKLELPHENEL
Tal-PER         EVLRHETRHPTVSPMQHENCVGIKVPASKRRRTLAEMMSCLVNDIHAQGMKNLTSAAKIS
Ep-PER          ------------------YQDPCKEPMVKRRRTLADIMSPFVEELDNIEKKNSGDYHQTN
Peram-PER       ------------------NGHLEKQQMSKRCRDLATFMESLMDDITKPDLKLELPQE-EH
                                       *    :* : ** :*. :::::     *

Drome-PER       TVSER---------------------------------DSVMLGEISPHHDYYDSKSSTE
Tal-PER         TLPTSGISTSTSTAVTTSSAAINNSMAVQTSAPARNAVEPSASAAVTQAAKGESLLSSSE
Ep-PER          LSMSCGVGRKSVGVSIANEGHMGHGTPLHKNS-LRHCQSGVMPSIITPGQ--ESLNSSIE
Peram-PER       SFSEH---------------------------------DSVMLGEISPHHDYYDSKSSTE
                                                      .    . ::      .  ** *

Drome-PER       TPPSYNQLNYNENLLRFFNSKPVTAPAELDPPKTEPPEPRGTCVSGASGPMSPVHEGSGG
Tal-PER         SPSTYSRLNYSATMLRFFQSNPRTASTD---------ASAESKMESSIRGSVDEAKSSSP
Ep-PER          TPSSYGQLNYSATLQRFFRSNPKTVSSD---------ESGDSKMEISHPESIGFSTSASA
Peram-PER       TPPSYNQLNYNDNIQRFFESKPKTTLSD---------ESGESKIEANRSLMSTDEEGKSG
                :*.:*.:***. .: ***.*:* *. ::          .  : :.           . .

Drome-PER       SGSSGNFTTASNIHMSSVTNTSIAGTGGTGTGTGTGTGTGTGTGTGTGTGTGTGTGTGTG
Tal-PER         KQQYCKFSNSVPSGVNKSL-----------------------YMSGYGSGSGDTIYLTRK
Ep-PER          NRKSQSQSQSQSQSQTQSQ-----------------------SGSGSGSGDNNFNFESNS
Peram-PER       PAADSSLGSSNRKCCSPVN--------------------GSGSGSGSGHSSGS-------
                     .   :     .                            :* * . .

Drome-PER       TGTGTGTGNGTNSGTGTGTASSSKGGSAAIPPVTLTESLLNKHNDEMEKFMLKKHRESR-
Tal-PER         YGSKDVTDSGSGESASGPSSASGGAGSANYRHVPLTEEVLSRHNQEMQLFMERQKKNTV
Ep-PER          FRKSNTKCKFAKNEESG----EGQSTDDQPYMLPLTEEILSRHNQHMQKQFMECQRKNER
Peram-PER       ----AGIGGSAESRRDTSATNTSHG---SYKPPLLTEALLCRHNEDMEKKMVQKHREQR-
                          : .  .      . .         *** :* :**:.*:  ::: :::.

Drome-PER       -----------------GRTGEKSKKSANDTLKMLEYSGPGHGIKRGGSHSWEGEANKPK
Tal-PER         AITTPSRQRERLSRQKLKQVKKASRKPPSKCALKRPNSGSQRGDNRAHKFPFIEKTSAKQ
Ep-PER          ILNSNK------YDFKTRTFKKHTKKRERSYAMKRSCTPKVYEGN-AHKTTHINSSAANS
Peram-PER       -----------------NKGSERESK-LKKCVHDKLLQEQCHGVKRSGSHSWEGEVYK--
                                     :   *   .              : . . .   .

Drome-PER       QQLTLGTDAIKGAAGSAGGAVGTGGVGSGGAGVAGGGGSGTGVAGTPEGRATTTSGTGTP
Tal-PER         SEQTGPSHLPGPLHTTHGNKLTINSFHSGSGSRADHVTRRTDLAVPVIPATCSSEQPQAP
Ep-PER          NKTLHISAMNTAKSSIAPGNEGSKREKSFPSS---PMVSRQVPNIPDGRTMKGEAGPSFQ
Peram-PER       --------ASKHPHVEGGKELNPGSGGSGGGGVTSGLSQCLGVAAT--GKQYSGVGSVPP
                                           *  ..             .          .

Drome-PER       GGAGGGGGAGAAAAAGASSSVGSSTPGPSSYPTCTQNINLWPPFSVGITPPVHSTHTAMA
Tal-PER         YFFPGHPLPTPSNGFQNTSLPSNPSTSLSQEPQLPSNPHPYPMTPMMMGPHQTHFMTPQG
Ep-PER          RVLPNYYIHANHTSSPVKSTEPIVGHSTLPPCPVSSAGTPFSAPPLMMPPQTTFLPSQP-
Peram-PER       IFQGG------------------------------TNVNLWPPFSVTVTP----------
                    .                                   :.  .: : *

Drome-PER       -QSSFSSAGLFPTFYYIPASLTPTSPTRSPRMHKHPHKGGTDMPTTSQQAAAAAAQA---
Tal-PER         LALPLHYMGAYPGMYIPHPSFNQAMYGAGPLMMSNMMMPHPFIQQSTSADPLHVSPTNLN
Ep-PER          --LPIQYVTGLPGVIYQP--IAPPLFSAPPVMLPNLVFQHTLVQPPMGHMQVPILTD---
Peram-PER       --------------------LQPTPPCSTHGFATN------NIPTP--------------
                                    :  .       :  :       :  .

Drome-PER       -----------------------------MPLQYMAGVMYPHPSLFYTHPAAAAATAMMY
Tal-PER         TPSRQADNQSYIAQVAPVERSSQSLQQRHTDHHHTHSHHQQQAQLQQPLPHPKSPHGVVH
Ep-PER          -----------------------------ERRYFTESEHETTETTHQATKSLQTDKQVVT
Peram-PER       ---------------------------------------------------PHMASMIPVYY
                                                                     :   :
```

```
Drome-PER        QPMPFPGMANALQIPERPLGSQSAYN---------------------------KSVYTTT
Tal-PER          LRKRSDSRATSVKVEPGSVRGSVASASGQLRCSMSHRPESLRSETDEPGHAEQGLVYVPR
Ep-PER           LR-RPDSQATSVKAEPGSVRGSNASASCKVNAAESIRSYVDDLSLLQNIPNSKQDRSHTP
Peram-PER        IEKRAPNFKNN-------------------------------------------------
                       .  .

Drome-PER        PASMTKKVPGAFHSVTTPAQVQRPSSQSASVKTEPGSSAAVSDPCKKEV-----------
Tal-PER          VASHASHFSRSTSVLGEAESIASPDKRPSGMEDQHAE--NSSDTVMSDS-----------
Ep-PER           NYSQVSPFSQSTSVIAEPESVRSPEKQDRLVEDTETKNYNSSDMILSMDSYSGPDDKSDE
Peram-PER        ------------------------------------------------------------

Drome-PER        -------PDSSPIPSVMGDYNSDPPCSSSNPANNKKYTDSNGNSDDMDGSSFSSFYSSFI
Tal-PER          ----SPAQDSMPMLSEFSVDTSESIGVIQPPCDDRPVLHDPAWLDHIEVTPQLLFRYQLH
Ep-PER           EDSPSPDMGTSKDLEEDNERRDGSKMSMKRKDGTRPVLCEPPWIEDVKVTPELLFRYQLK
Peram-PER        ------------------------------------------------------------

Drome-PER        KTTDGSESPPDTEKDPKHRKLKSMSTSESKIMEHPEEDQTQHGDG---------------
Tal-PER          TKELVDVLKNDMAALKTLNQPALVEDQLSSLYQELEIDGEQLQLDEGITSSSGEEMLDVS
Ep-PER           TSELVDVLRQDMDLLKNTKQVKKMNMYWKACWIYKFNRTYYTQRNRAHICV---------
Peram-PER        ------------------------------------------------------------

Drome-PER        ------------------------------------------------------------
Tal-PER          TKTSSEAGAARGAKKARSSRYFDKQAILHEVEAAIPPPDLSISHISPYSVARRLPNKHKS
Ep-PER           ------------------------------------------------------------
Peram-PER        ------------------------------------------------------------

Drome-PER        -
Tal-PER          S
Ep-PER           -
Peram-PER        -
```

Figure S2. Putative *Talitrus saltator* PERIOD (PER) protein

Alignment of *Drosophila melanogaster* PER (Drome-PER; Accession No. AAF45804) with the *T. saltator* PER (Tal-PER) deduced from the Trinity *de novo* transcriptome assembly, together with the top two tblastn species homologue sequences *Eurydice pulchra* PER (Ep-PER; Accession No. KC885967) and *Periplaneta americana* (Peram-PER; Accession No. U12772). '*' indicates identical amino acid residues in the two proteins, '.' and ':' indicate similar amino acid residues between the two proteins. In this figure SMART identified domains of two PAS domains, one PAC domain and one Period C domain are highlighted in yellow, green and blue respectively. The region of Ep-PER that corresponds to the PAC domain is shown in green text, though no PAC domain *per se* was identified in this protein using SMART. Drome-PER DBT/CK1ε binding site is highlighted in pink. Dark red text indicates Drome-PER CLK/CYC inhibitory domain (CCID). Pink text indicates regions in Tal-PER, Ep-PER and Peram-PER corresponding to sequence within both the overlapping Drome-PER DBT/CK1ε binding site and CCID regions.
