# Supplementary material for: Identification and temporal expression of putative circadian clock transcripts in the amphipod crustacean Talitrus saltator
Source: PeerJ. 2016 Oct 5;4:e2555. doi: 10.7717/peerj.2555 (PMC5068443; doi:10.7717/peerj.2555)
Supplement: Figure S3 — Alignment of Drosophila melanogaster TIM (Drome-TIM; Accession no. AAC46920) with the T. saltator TIM (Tal-TIM) deduced from the Trinity de novo transcriptome assembly, together with the top two tblastn species homologue sequences Lottia gigantea hypothetical protein partial (Lotgi; Accession No. XM_009048115) and Tribolium castaneum TIM (Trica-TIM; Accession No. XM_008202829). 3’ sequence removed from all sequences except Tal-TIM. ’*’ indicates identical amino acid residues in the two proteins, ’.’ and ’:’ indicate similar amino acid residues between the two proteins. In this figure the SMART identified Timeless domain is highlighted in yellow. [file peerj-04-2555-s003.pdf]

```
Drome-TIM       MSRVRQLHNHIWNNQNFDKVKSVMDWLLATPQLYSAFSSLGCLEGD--TYVVNPNALAIL
Tal-TIM         ------------------------------------------------------------
Lotgi           --------------------------MVMHVELQATCSALGYLEGK--RYIKEPDCLETV
Trica-TIM       ------------------------MSSLLSAELAATCSALGYYDATSNKYYADSNTLETV


Drome-TIM       EEINYKLTYEDQTLRTFRRAIGFGQNVRSDLIPLLEN-AKDDAVLESVIRILVNLTVPVE
Tal-TIM         ------------------------RVLQTDWLPLLREHHSDSALLDLVLRLLVNLTTPAL
Lotgi           KDLIRFLKREDDTC-DIRRQLGHAGIVQNDLLQLIKYYKDDAVLFDTVLRLLVNLTQPAY
Trica-TIM       KDLIRYLRRDDESH-DIRRQLGEIKVLQNDLVPLLKSYWEETDLFDVLLRLLVNLTTPAL
                                          ::.* : *:.   .:  ::: ::*:***** *.

Drome-TIM       CLFSVDVMYRTDVGRHTIFELNKLLYTSKEAFTEARSTKSVVEYMKHILESD-PKLSPHK
Tal-TIM         LVFHQEIPE-DKAGREMYLRLVSQQQGFKEAFTDAGVWASVAGVLGSRLQQG-SERDDDA
Lotgi           LCFNNQIPE-DKTLRNYYLEIESHLYNYKEAFVDEELFAVLTEKIGDILKLDWEHRQEED
Trica-TIM       MLWNEELPT-DKITRNHYLQIEDHLKSYKQTFADETFWAVLSTRLSKILETSYAERGDEN
                  :  ::    .  *.  :.: .     *::*.:      :   :   *: .  .   .

Drome-TIM       CDQINNCLLLLRNILHIPETHAHCVMPMMQSMPHGISMQNTILWNLFIQSIDKLLLYLMT
Tal-TIM         NLIIEMCLVLLRNVLAVAPGHQ-----DTTRTSDDADLHDQVLWSLHLAGIPDLLLYLST
Lotgi           KLTLERMLILLRNVLHIAPDPS-----MEKRTDDDASLHDQVIWVFHVSGMEDMLLYIAS
Trica-TIM       SLIIERILILIRNILYVPPDPN-----TEKRPDNDASIHDQVLWALHQSGMLDIILFITS
                   ::  *:*:**:* :.               .. .::: ::* :.  .: .::*:: :

Drome-TIM       CPQRAFWGVTMVQLIALIYKDQHGSGDSSPMLTSDPTSDSSDNGSNGRGMGGGMREGTAA
Tal-TIM         STDESDLSLHTLEIISLMLRQQDPQNLAGSALHRSAEEQRKD------------------
Lotgi           ADDERQLCMHILEIISLMFREQKPEVLASAGVKKSKSEKEAE------------------
Trica-TIM       STNEKAYYMHTLEILSFMLREQKAAELARAALQRSETEKMRD------------------
                . :.    :  ::::::: ::*.    : . :  .  ..  :

Drome-TIM       TLQEVSRKGQEYQNAMARVPADKPDGSEEASDMTGNDSEQPGSPEQSQPAGESMDDGDYE
Tal-TIM         ------------------------------------------------------------
Lotgi           ------------------------------------------------------------
Trica-TIM       ------------------------------------------------------------


Drome-TIM       DQRHRQLNEHGEEDEDEDEVEEEEYLQLGPASEPLNLTQQPADKVNNTTNPTSSAPQGCL
Tal-TIM         ------------------------------AEALVQVRQAEKARRQQQVRKHYNARHSRF
Lotgi           ------------------------------ERELEMIREQERAKRRAEFLKQS-SRHSRF
Trica-TIM       ------------------------------EAELLAIRHRETNQKQQKIKLYNGARHSRF
                                                    : .    : .        : :. :

Drome-TIM       GNEPFKPPPPLPVRASTSAHAQMQKFNESSYASHVSAVKLGQKSPHAGQLQLTKGKCCPQ
Tal-TIM         GG----------------------------------------------------------
Lotgi           GG----------------------------------------------------------
Trica-TIM       GG----------------------------------------------------------
                *.

Drome-TIM       KRECPSSQSELSDCGYGTQVENQESISTSSNDDDGPQGKPQHQKPPCNTKPRNKPRTIMS
Tal-TIM         ----------------TYYVQNMKSISDRDVIAHKPVADVNSFNFDQNKRGKKIPKNRAP
Lotgi           ----------------TYVVKNLKSISEREMIYHKAKGDVEAITLNDKKRRTKIAKNRQP
Trica-TIM       ----------------TYVLKSMKSISDNELIYHKPLNKLEALNFDADKRKPKTPKNRMP
                                   ::. :***  .   . .  . :  .   ..:  : .:.  .

Drome-TIM       PMDKKELRRKKLVKRSKSSLINMKGLVQHTPTDDDISNLLKEFTVDFLLKGYSYLVEELH
Tal-TIM         LPDSAVTRRSTLAIR----------------------LFLQEFCVEFLNGAYNNIMSIVK
Lotgi           IKNTEVCRRSTLSIR----------------------LCLKEFCVQFLENCYNPIMFAVK
Trica-TIM       VQSSTFERRSAFSIR----------------------LFLKEFCVEFLNGAYNTLMYHVK
                  ..   **. :  *                        *:** *:**   *. ::  ::

Drome-TIM       MQLLSNAKVPIDTSHFFWLVTYFLKFAAQLELDMEHIDTILTYDVLSYLTYEGVSLCEQL
Tal-TIM         DNLNRARVQEHDESYYLWAMKFFMEFNRHHEFKVELVTETLSIQSVHYVQTNIETYHEMM
Lotgi           DNLVREKTQDHDETYYLWSVRFFMEFCRFHSKRVELVSETMSTTTFHYIYTNLLNYYEMM
Trica-TIM       DNLVRARAQAHDESYYLWALRFFMEFNRCYKFEVKLVSETMAVQTFRYVQQLTEKYFDMI
                 :*        * ::::* : :*::*    .  :: :   ::   . *:     .  : :
```

```
Drome-TIM       ELNARQEGSDLKPYLRRMHLVVTAIREFLQAIDTYNKVTHLNEDDKAHLRQLQLQISEMS
Tal-TIM         TT----EKKKIPLWSRRRHNGLRAYQEIMMSLSAMDKSP--DQ-----------------
Lotgi           IT----EKKEAKVWGRRAHLALKAYQELMLTLDSMDRSG--NPQVMESSKVIKGNLFYMM
Trica-TIM       QS----DKKNAVLWSRRLHLALLAYRELFLTLCAMDKSP--DETVRDSARVIKSNIFYIV
                      : ..   : ** *  : * :*:: :: : ::    :
```

Figure S3. Putative *Talitrus saltator* TIMELESS (TIM) protein

Alignment of *Drosophila melanogaster* TIM (Drome-TIM; Accession no. AAC46920) with the *T. saltator* TIM (Tal-TIM) deduced from the Trinity *de novo* transcriptome assembly, together with the top two tblastn species homologue sequences *Lottia gigantea* hypothetical protein partial (Lotgi; Accession No. XM_009048115) and *Tribolium castaneum* TIM (Trica-TIM; Accession No. XM_008202829). 3' sequence removed from all sequences except Tal-TIM. '*' indicates identical amino acid residues in the two proteins, '.' and ':' indicate similar amino acid residues between the two proteins. In this figure the SMART identified Timeless domain is highlighted in yellow.
