# Supplementary material for: Identification and temporal expression of putative circadian clock transcripts in the amphipod crustacean Talitrus saltator
Source: PeerJ. 2016 Oct 5;4:e2555. doi: 10.7717/peerj.2555 (PMC5068443; doi:10.7717/peerj.2555)
Supplement: Figure S4 — Alignment of Drosophila melanogaster CLK (Drome-CLK; Accession No. AAC62234) with the T. saltator CLK (Tal-CLK) deduced from the Trinity de novo transcriptome assembly, together with the top two tblastn species homologue sequences Macrobrachium rosenbergii CLK (Macro-CLK; Accession No. AY842303) and Eurydice pulchra CLK (Ep-CLK; Accession No. KC885976). 3’ sequence removed from all sequences. 5’ sequence removed from all sequences except Tal-CLK to aid alignment. ’*’ indicates identical amino acid residues in the two proteins, ’.’ and ’:’ indicate similar amino acid residues between the two proteins. In this figure SMART identified domains consisting of one PAS domain and one PAC domain are highlighted in green and blue respectively. [file peerj-04-2555-s004.pdf]

```
Drome-CLK       -----------------GMEKVDANAYELVKFVGYFR----------NDTNTSTGSSSEV
Tal-CLK         -------------------------------------------------QQHQQQPQQHQ
Macro-CLK       ----------------------SVSSCSRSMFGGNAG----------GGVTGSSNAGSGL
Ep-CLK          PEEYRERYTIAIHIQRGGMTPTAVPVYERIHLTGYFERYKCPSEDGVLDFSCSEAEDSSM
                                                                    .    .

Drome-CLK       SNGSNGQPAVLPRIFQQNPNAEVDKKLVFVGTGRVQNPQLIREMSIIDPTSNEFTSKHSM
Tal-CLK         QPQHPPQRPQEQQQQLQRNDQPEQNQPVFVGICRLEQPQLLREMRLLERTNTEFVSRHSL
Macro-CLK       CQSS---------LVQTNPSQEP-TKLVFVAIGRLERPQLVREMMIIEPSKTEFTSRHSL
Ep-CLK          SVGGTSRSSNPSPFTHSQHHSPEPTKIVFVAIARLEHPQLLRELIVLEPTKSEFTSRHSL
                                 .      .: ***.  *::.***:**: ::: :..**.*:**:

Drome-CLK       EWKFLFLDHRAPPIIGYMPFEVLGTSGYDYYHFDDLDSIVACHEELRQTGEGKSCYYRFL
Tal-CLK         EWKFLFLDQRASAIIGYLPFEVLGTSGYDYYHVDDLERVSTCHQFLIRTGKGSSCYYRFL
Macro-CLK       EWKFLFLDHRAPTIIGYLPFEVLGTSGYDYYHVEDLDKVASCHEQLMKTGKGTSCYYRFL
Ep-CLK          EWKFLFLDHRAPTIIGYLPFEVLGTSGYDYYHVDDLEKVSECHEMLMKKGKGISCFYRFL
                ********:**..****:**************.:**: :  **: * :.*:* **:****

Drome-CLK       TKGQQWIWLQTDYYVSYHQFNSKPDYVVCTHKVVSYAEVLKDSRKEGQKSGNSNSITNNG
Tal-CLK         TKGHQWVWLQSHYYISYHQWNSKPEFVVCTNTVVSYDDIKAELNGSSTNYNDSSTNLQTS
Macro-CLK       TKGQQWIWLQTQYYITYHQWNSKPEFIVCTNTVVSYSDVKAELVKEQMPNGLSELEINQS
Ep-CLK          TKGQQWIWLRSHYCITYHQWNSKPEFIVCTNTVVSYGNIKGNNQQNHSEREEKSDMEFES
                ***:**:**::.* ::***:****:::***:.**** ::  :   .      ..     .

Drome-CLK       SSKVIASTGTSSKSA---------------------------------------------
Tal-CLK         SLTEDKTVSSSCPSQNCTFAEKIDPETKCKSEQNQRFFQKIQSFEEQCMNSSSRAEQSSS
Macro-CLK       ES----SMGLSGAGP---------------------------------------------
Ep-CLK          HSQP-TSEGGGSFAP---------------------------------------------
                      : . .  .
```

Figure S4. Putative *Talitrus saltator* CLOCK (CLK) protein

Alignment of *Drosophila melanogaster* CLK (Drome-CLK; Accession No. AAC62234) with the *T. saltator* CLK (Tal-CLK) deduced from the Trinity *de novo* transcriptome assembly, together with the top two tblastn species homologue sequences *Macrobrachium rosenbergii* CLK (Macro-CLK; Accession No. AY842303) and *Eurydice pulchra* CLK (Ep-CLK; Accession No. KC885976). 3' sequence removed from all sequences. 5' sequence removed from all sequences except Tal-CLK to aid alignment. '*' indicates identical amino acid residues in the two proteins, '.' and ':' indicate similar amino acid residues between the two proteins. In this figure SMART identified domains consisting of one PAS domain and one PAC domain are highlighted in green and blue respectively.
