# Supplementary material for: Identification and temporal expression of putative circadian clock transcripts in the amphipod crustacean Talitrus saltator
Source: PeerJ. 2016 Oct 5;4:e2555. doi: 10.7717/peerj.2555 (PMC5068443; doi:10.7717/peerj.2555)
Supplement: Figure S5 — Alignment of Drosophila melanogaster CYC (Drome-CYC; Accession No. AAF49107) with the T. saltator BMAL1 (Tal-BMAL1) deduced from the Trinity de novo transcriptome assembly, together with the top two tblastn species homologue sequences Pacifastacus leniusculus BMAL1A (Pacle-BMAL1A; Accession No. JQ670886) and Eurydice pulchra BMAL1 (Ep-BMAL1; Accession No. KC885968). ’*’ indicates identical amino acid residues in the two proteins, ’.’ and ’:’ indicate similar amino acid residues between the two proteins. In this figure SMART identified domains of bHLH domain, PAS domains and PAC domain are highlighted in yellow, green and blue respectively. Red text indicates sequence with high similarity to C-terminal transactivation domain of Mus musculus BMAL1. [file peerj-04-2555-s005.pdf]

```
Drome-CYC           ------MEVQEFCEN-------------------MEEIEDENYDEEKSARTS-DENRKQN
Tal-BMAL1           -------SVASLSSDGVN--------IKKKLPTLGESHNDEDLDCSKNNRSSAEWNKRQN
Pacle-BMAL1A        MFGMSNFDYPKYRSECNSLASFSSDGGSKKRRGSFIDSNDDDADSIKIPRTAGDWNKRQN
Ep-BMAL1            MFGVVGFDYPEYRSDSASGASYPSEGASKKRRGSTAESTEDDKDSSKMPRTSTDWNKRQN
                           .  .  .:                     .  ::: *  *  *:: : *::**

Drome-CYC           HSEIEKRRRDKMNTYINELSSMIPMCFAMQRKLDKLTVLRMAVQHLRGIRGSGSLHPFNG
Tal-BMAL1           HSEIEKRRRDKMNTYISELSRMIPQC--QSRKLDKLSVLRMAVQHIKMLRG--SINSYTE
Pacle-BMAL1A        HSEIEKRRRDKMNTYIMELSSIIPVC--TSRKLDKLTVLRMAVQHMKMLRG--SLNSYTE
Ep-BMAL1            HSEIEKRRRDKMNTYIMELSSIIPVG--TSRKLDKLTVLRMAVQHMKMLRG--SLNSYTE
                    **************** *** :**     .******:********:: :**  *::.:.

Drome-CYC           SDYRPSFLSDQELKMIILQAS-EGFLFVVGCDRGRILYVSDSVSSVLNSTQADLLGQSWF
Tal-BMAL1           GQYKPSFVSDEQVQQLLQQQCTEGFLFVVGCDRGKILFVSESVSHILQYSQCELLGLSWF
Pacle-BMAL1A        GHYKPAFLSDDELKNLILQAA-DGFLFVVGCDRGRILYVSESVYQTLHYTQGELLGTSWF
Ep-BMAL1            GHYKPSFLSDDELKNLILQAA-DGFLFVVGCDRGRIIYVSESVSQTLQYTHSELLGTSWF
                    ..*:*:*:**:::: :: * . :***********:*::**:**   *: :: :*** ***

Drome-CYC           DVLHPKDIGKVKEQLSSLEQCPRERLIDAKTMLPVKT--------------DVPQSLCRL
Tal-BMAL1           DILHPKDLTKVKEQLSCGDISRRERLVDAKTLLPVHHSPNSSSSSSCGNYPSLPQDLTRL
Pacle-BMAL1A        DILHPKDLTKVKEQLSCSDISRRERLVDAKTLLPVKT--------------DVPQGLTKL
Ep-BMAL1            DILHPKDLAKVKEQLSSADINRRDRLVDAKTLLPVKT--------------DVPQGLSKL
                    *:*****: *******. :   *:**:****:***:               .:**.* :*

Drome-CYC           CPGARRSFFCRMKLRTASNNQIKEESDTSSSSRSSTKRKSRLTTGHKYRVIQCTGYLKSW
Tal-BMAL1           CPGSRRAFYARIRRPNVH----KAPADDASD------DASAMTGDKRYMSIHFTGYLKSW
Pacle-BMAL1A        CPGSRRAFFCRMRCKTSP--VLKEEADSSTG--CPRKKSKSQSSDKKYSVIHFTGYLKSW
Ep-BMAL1            CPGFRRSFFCRMRCRSAH--VIKEEADTTTG--CHKKKNKPHCSDKKYSVIHFTGYLKSW
                    *** **:*:.*::  .      *  :* ::.        .    .::*  *: *******

Drome-CYC           TPIKD-EDQDADSDEQTTNLSCLVAIGRIPPNVRNSTVPAS--LDNHPNIRHVLFISRHS
Tal-BMAL1           HGAFRPAGVGDE-DHDSGDAACLVAIGRLHRPLSCASVPLK-------------FVAKLS
Pacle-BMAL1A        APTKDPLEEDSGSDSDSCNLSCLVAVGRVHQPLLASSVLDTSRLGSAAPTQPIEFISKHT
Ep-BMAL1            APTKEPVEEETEVDSDSCNLSCLVAVGRVHQPLLSSVPKDTTRLSRFLPTKPIEFISKHN
                                 * :: : :****:**:   :  :    .             *::: .

Drome-CYC           GEGKFLFIDQRATLVIGFLPQEILGTSFYEYFHNEDIAALMESHKMVMQVPEKVTTQVYR
Tal-BMAL1           PEAKYNYVDQRMSVVLGWLPQEVLGASVFELSHPSDHCRLAAAHRALLSKTTVVESLQHR
Pacle-BMAL1A        NDGKFVFIDQRASLLLGWLPQELLGSSMYEYFHQDDIAFLAETHKTTLQNSESCNTQVYR
Ep-BMAL1            SEGKFVFIDQKASLLLGWLPQELLGTSMYEYYHQDDIPILAEAHRLLLQANEPTQTQVYR
                     :.*: ::**: ::::*:****:**:*.:*  * .*   *  :*:  :.      :  :*

Drome-CYC           FRCKDNSYIQLQSEWRAFKNPWTSEIDYIIAKNSVFL-----------------------
Tal-BMAL1           CRHKDGRWVRLEGSWKLFINPWTNELEYIVASNTVFSDDKL----SSTDDSSQRNSLLQY
Pacle-BMAL1A        FRTKEGSFVRLQSNWWTFKNPWTKEIEYIISKNSVVTSEAELVESTMANESVS-QSYSNF
Ep-BMAL1            FRTKDGSFMNFKSMWKTFKNPWTKDVEYIISNNFLILSEGE-VESTMVSDCASSQSFGNI
                     * *:. ::.::. *  * ****.:::**::.* :.

Drome-CYC           ------------------------------------------------------------
Tal-BMAL1           GDSVPPDP----------SLSPSLPSLTPSLGATLQQNGGGSAVPDAPAS------TCGS
Pacle-BMAL1A        SEYLNSPDPNHNQDTSPIGGAGSNNSNSRLIGGGIQAGKIGRGIADEVLDNQWKNDSTCK
Ep-BMAL1            SEFINSPG----------GGSSSSVSSSRMMGNGVQANKIGRKIAEEILDPHRRPESPAS

Drome-CYC           ------------------------------------------------------------
Tal-BMAL1           DVTDSYTVIQGQAVGSSSLASIQMARAVSGDGSDDTASQCRASGAGEMVSDQPSTEGKDA
Pacle-BMAL1A        SPVSPFEGILGSGASDRSFAALLRSDMTAHRTNVKNNAVLSSNTSTCSESDTSRSTQLSN
Ep-BMAL1            TPISPLEAILG-ANSDRSFSSLLKPETDSSR--MMN--ICGSSTDTSVDSDSTSKQQAAK

Drome-CYC           ------------------------------------------------------------
Tal-BMAL1           SSGDGNPLQLN--LDVPARLPFLHHYN---------------ARSESEASGVGEATSDSD
Pacle-BMAL1A        NHHRLPSMHNNNLINSSERGGSFSHNNSHRQPVS---NEGDLMDVVSGRDLETDGTSDSD
Ep-BMAL1            SHKPLLPPQ------PPKPENTLNHTNNHLQQVAQVPNNTDLMDVVSGRDVESEVTSDSD
```

```
Drome-CYC          -------------------------------
Tal-BMAL1          EAAMAIIMSLLEADAGLGGPAD---------
Pacle-BMAL1A       EAAMAVIMSLLEADAGLGGPVDFSHLPWPLP
Ep-BMAL1           EAAMAVIMSLLEADAGLGEPVDFSHLPWPLP
```

Figure S5. Putative *Talitrus saltator* BMAL1 protein

Alignment of *Drosophila melanogaster* CYC (Drome-CYC; Accession No. AAF49107) with the *T. saltator* BMAL1 (Tal-BMAL1) deduced from the Trinity *de novo* transcriptome assembly, together with the top two tblastn species homologue sequences *Pacifastacus leniusculus* BMAL1A (Pacle-BMAL1A; Accession No. JQ670886) and *Eurydice pulchra* BMAL1 (Ep-BMAL1; Accession No. KC885968). '*' indicates identical amino acid residues in the two proteins, '.' and ':' indicate similar amino acid residues between the two proteins. In this figure SMART identified domains of bHLH domain, PAS domains and PAC domain are highlighted in yellow, green and blue respectively. Red text indicates sequence with high similarity to C-terminal transactivation domain of *Mus musculus* BMAL1.
