# Supplementary material for: Identification and temporal expression of putative circadian clock transcripts in the amphipod crustacean Talitrus saltator
Source: PeerJ. 2016 Oct 5;4:e2555. doi: 10.7717/peerj.2555 (PMC5068443; doi:10.7717/peerj.2555)
Supplement: Figure S6 — Alignment of Drosophila melanogaster CKII-α and CKII-β (Drome-CKII-α and Drome-CKII-β; Accession No.s AAN11415 and AAF48093 respectively) with the T. saltator CKII-α and CKII-β (Tal-CKII-α and Tal-CKII-β) deduced from the Trinity de novo transcriptome assembly, together with the top two Tal-CKII-α tblastn species homologue sequences Harpegnathos saltator CKII-α (Harsa-CKII-α; Accession No. XM_011140473) and Orussus abietinus CKII-α (Oruab-CKII-α; Accession No. XM_01242321) and the top two Tal-CKII-β tblastn species homologue sequences Atta cephalotes CKII-β (Attce-CKII-β; Accession No. XM_012208415) and Orussus abietinus CKII-β (Oruab-CKII-β; Accession No. XM_012432316). 3′sequence removed from Harsa-CKII-α.’*’ indicates identical amino acid residues in the two proteins, ’.’ and ’:’ indicate similar amino acid residues between the two proteins. In this figure SMART identified domains of serine/threonine protein kinase catalytic domain and casein kinase regulatory subunit domain are highlighted in yellow and green respectively. [file peerj-04-2555-s006.pdf]

```
Drome-CKII-α      -MTLPSAARVYTDVNAHKPDEYWDYENYVVDWGNQDDYQLVRKLGRGKYSEVFEAINITT
Tal-CKII-α        MMPFRSRARVYADVNTLRPQDYWDYESHLIEWGQQDDYQLVRKLGRGKYSEVFEAININN
Harsa-CKII-α      -MALPSRARVYADVNSHKPREYWDYESYVVDWGQQDDYQLVRKLGRGKYSEVFEAINVTN
Oruab-CKII-α      -MALPSRGRVYADVNSHKPRDYWDYESYVVDWGQQDDYQLVRKLGRGKYSEVFEAINVTN
                   *.: *  .***:***: :*  :*****.::::**:***********************:..

Drome-CKII-α      TEKCVVKILKPVKKKKIKREIKILENLRGGTNIITLLAVVKDPVSRTPALIFEHVNNTDF
Tal-CKII-α        NEKCVVKILKPVKKKKIKREIKILENLRGGTNIITLQAVVKDPVSRTPALVFEHVNNTDF
Harsa-CKII-α      NEKCVVKILKPVKKKKIKREIKILENLKGGTNIITLQAVVKDPVSRTPALIFEHVNNTDF
Oruab-CKII-α      NEKCVVKILKPVKKKKIKREIKILENLRGGTNIITLQAVVKDPVSRTPALIFEHVNNTDF
                  .**************************:******** *************:*********

Drome-CKII-α      KQLYQTLTDYEIRYYLFELLKALDYCHSMGIMHRDVKPHNVMIDHENRKLRLIDWGLAEF
Tal-CKII-α        KQLYQTLNDYDIRYYLYELLKALDYCHSMGIMHRDVKPHNVMIDHENKRLRLIDWGLAEF
Harsa-CKII-α      KQLYQTLNDYDIRYYLYELLKALDYCHSMGIMHRDVKPHNVMIDHENRKLRLIDWGLAEF
Oruab-CKII-α      KQLYQTLTDYDIRYYLYELLKALDYCHSLGIMHRDVKPHNVMIDHENRKLRLIDWGLAEF
                  *******.**:*****:***********:******************::***********

Drome-CKII-α      YHPGQEYNVRVASRYFKGPELLVDYQMYDYSLDMWSLGCMLASMIFRKEPFFHGHDNYDQ
Tal-CKII-α        YHPGQEYNVRVASRYFKGPELLIDYQMYDYSLDMWSLGCMLASMIFRKEPFFHGHDNYDQ
Harsa-CKII-α      YHPGQEYNVRVASRYFKGPELLVDYQMYDYSLDMWSLGCMLASMIFRKEPFFHGHDNYDQ
Oruab-CKII-α      YHPGQEYNVRVASRYFKGPELLVDYQMYDYSLDMWSLGCMLASMIFRKEPFFHGHDNYDQ
                  **********************:*************************************

Drome-CKII-α      LVRIAKVLGTEELYAYLDKYNIDLDPRFHDILQRHSRKRWERFVHSDNQHLVSPEALDFL
Tal-CKII-α        LVRIAKVLGTEELFEYVEKYHVELDPRFNDILGRHSRKRWERFVHHENQHLVSPEALDFL
Harsa-CKII-α      LVRIAKVLGTEELFEYLDKYHIELDPRFNDILGRHSRKRWERFVHSENQHLVSPESLDFL
Oruab-CKII-α      LVRIAKVLGTEELFEYLEKYHIDLDPRFNDILGRHSRKRWERFVHSENQHLVSPESLDFL
                  *************: *::**:::*****:*** ************ :********:****

Drome-CKII-α      DKLLRYDHVDRLTAREAMAHPYFLPIVN--GQMNPNNQQ---------------------
Tal-CKII-α        DKLLRYDHQERLTAHEAMEHPYFAPIVKDQGIMIGSPPPQQPPPAVISGIQDE-------
Harsa-CKII-α      DKLLRYDHYERLTAREAMEHPYFYPIVKEQGRLTMVSSSPTPMTGSVPVGDHYRSSWRNN
Oruab-CKII-α      DKLLRYDHYERLTAREAMEHPYFYPIVKEQGRLTIVSSSPTPMPGSLPLDE---------
                  ******** :****:*** **** ***:  * :

Drome-CKII-β      ------MSSSEEVSWVTWFCGLRGNEFFCEVDEDYIQDKFNLTGLNEQVPNYRQALDMIL
Tal-CKII-β        ------MSSSEEVSWIAWFCGLRGNEFFCEVDEDYIQDKFNLTGLNEQVPHYRQALDMIL
Attce-CKII-β      MTTETKMSSSEEVSWISWFCGLRGNEFFCEVDEDYIQDKFNLTGLNEQVPHYRQALDMIL
Oruab-CKII-β      ------MSSSEEVSWISWFCGLRGNEFFCEVDEDYIQDKFNLTGLNEQVPHYRQALDMIL
                        *********::*********************************:*********

Drome-CKII-β      DLEPEDELEDNPLQSDMTEQAAEMLYGLIHARYILTNRGIAQMIEKYQTGDFGHCPRVYC
Tal-CKII-β        DLEP-DEEEDLPHQGDLVEQAAEMLYGLIHARYILTNRGIAQMIEKYQAGDFGHCPRVYC
Attce-CKII-β      DLEPDDDLDDNPNQSDLIEQAAEMLYGLIHARYILTNRGIAQMIEKYQAGDFGHCPRVYC
Oruab-CKII-β      DLEPDDDIEDNPNQSDLIEQAAEMLYGLIHARYILTNRGIAQMIEKYQAGDFGHCPRVYC
                   **** *: :* * *.*: ******************************:**********

Drome-CKII-β      ESQPMLPLGLSDIPGEAMVKTYCPKCIDVYTPKSSRHHHTDGAYFGTGFPHMLFMVHPEY
Tal-CKII-β        ENQPMLPIGLSDVPGEAMVKLYCPNCCDVYTPKSSRYNHIDGSYYGTGFPHMLFMVHPEY
Attce-CKII-β      ESQPMLPLGLSDVPGEAMVKSYCPKCMDVYTPKSSRHHHTDGAYFGTGFPHMLFMVHPEY
Oruab-CKII-β      ESQPMLPLGLSDVPGEAMVKSYCPKCMDVYTPKSSRHHHTDGAYFGTGFPHMLFMVHPEY
                   *.*****:****:******* ***:* *********::* **:*:***************

Drome-CKII-β      RPKRPTNQFVPRLYGFKIHSLAYQIQLQAAANFKMPLRAKN------
Tal-CKII-β        RPKRPGTQFVPRLYGFKIHPMAYQLQQQAAANFNAPMRPVNFNNGKR
Attce-CKII-β      RPKRAANQFVPRLYGFKIHPVAYQIQQQSASTFKAPLRALNYNNGKR
Oruab-CKII-β      RPKRATNQFVPRLYGFKIHPLAYQIQQQSASTFKAPLRTVNYNNGKR
                   ****. .************.:***:* *:*:.*: *:*. *
```

Figure S6. Putative *Talitrus saltator* CASEIN KINASE II α and β (CKII-α and CKII-β) proteins

Alignment of *Drosophila melanogaster* CKII-α and CKII-β (Drome-CKII-α and Drome-CKII-β; Accession No.s AAN11415 and AAF48093 respectively) with the *T. saltator* CKII-α and CKII-β (Tal-CKII-α and Tal-CKII-β) deduced from the Trinity *de novo* transcriptome assembly, together with the top two Tal-

CKII-α tblastn species homologue sequences *Harpegnathos saltator* CKII-α (Harsa-CKII-α; Accession No. XM_011140473) and *Orussus abietinus* CKII-α (Oruab-CKII-α; Accession No. XM_01242321) and the top two Tal-CKII-β tblastn species homologue sequences *Atta cephalotes* CKII-β (Attce-CKII-β; Accession No. XM_012208415) and *Orussus abietinus* CKII-β (Oruab-CKII-β; Accession No. XM_012432316). 3' sequence removed from Harsa-CKII-α.'*' indicates identical amino acid residues in the two proteins, '.' and ':' indicate similar amino acid residues between the two proteins. In this figure SMART identified domains of serine/threonine protein kinase catalytic domain and casein kinase regulatory subunit domain are highlighted in yellow and green respectively.
