# Supplementary material for: Identification and temporal expression of putative circadian clock transcripts in the amphipod crustacean Talitrus saltator
Source: PeerJ. 2016 Oct 5;4:e2555. doi: 10.7717/peerj.2555 (PMC5068443; doi:10.7717/peerj.2555)
Supplement: Figure S7 — Alignment of Drosophila melanogaster CWO (Drome-CWO; Accession no. AAF54527) with the T. saltator CWO (Tal-CWO) deduced from the Trinity de novo transcriptome assembly, together with the top two tblastn species homologue sequences Metaseiulus occidentalis uncharacterized (Metoc; Accession No. XM_003744642) and Ixodes scapularis conserved hypothetical protein (Ixosc; Accession No. XM_002400882). 5′and 3′sequence removed from all sequences except Tal-CWO. ’*’ indicates identical amino acid residues in the two proteins, ’.’ and ’:’ indicate similar amino acid residues between the two proteins. In this figure SMART identified domains of bHLH domain and Orange of the Hairy/E(SPL) family domain are highlighted in yellow and green respectively. [file peerj-04-2555-s007.pdf]

```
Drome-CWO       RQDPLSHRIIEKRRRDRMNSCLADLSRLIPPQYQRKGRGRIEKTEIIEMAIRHLKHLQSE
Tal-CWO         ---PMSHRIIEKRRRDRMNNCLADLSRLIPAIYLKKGRGRIEKTEIIEMAIKHLKHLQAH
Metoc           KRDPMSHRIIEKRRRDRMNNCLADLSRLIPAIYLKKGRGRVEKTEIIEMAIKHLKHLQSH
Ixosc           ARDPMSHRIIEKRRRDRMNNCLADLSRLIPAVYLKKGRGRVEKTEIIEMAIKHLRHLQAH
                   *:**************.**********. * :*****:**********:**:***:.

Drome-CWO       CQQKESDYRSGYMDCMKEAAKFLYDVHMQDFCHRLLGRLQEHIDEMFKTDCYKSTRSCHM
Tal-CWO         SCKDPATCEVAHR--IETDHRHQYRLGFHECMS-ECVRFLVEIEGMYAGDDLCIRLMNHL
Metoc           ACNDPATCEVAQR--IDTDHRHQYRLGFQECMS-ECVRFLVDIEGRYANDEFCIRLMTHL
Ixosc           SCKDPTTCEVAQR--VDSDHRLQYRLGFQECLS-ETARFLVDLDGSCTADDTCFRLVAHL
                . :. :  . .    :.   :  * : :::       *:  .::     *        *:

Drome-CWO       PDNVSASSGSPHQAYHPPLCHLRDMLATSASDVEHSQDHNDVKDLSFRNHLNQLQRSQQA
Tal-CWO         QKHFDKVAGHGFCYQIPG--GSGPTQ---------------NNSSSPNP----------I
Metoc           KKHLDKLQGHNICYQVPGAVGSGPGQSTRTSPVASSSTASNGNSSSSSSGATTPATQIIQ
Ixosc           QKHFDKVSGAGSCYPAGLVLSDEEAT--------------------------------M
                 .:..   *

Drome-CWO       AAAAAVAAAAVAVANGSSPASNAGVDSKVPLTNGGGTGGAPPAADNVPSNSTGSGSAAAC
Tal-CWO         QVNTQSASISIQIRLPFKGM----------------------------------------
Metoc           QTPQQVPAIPIQIHPPSSGVVNVMGSPAATTRLSSDDVRSLESEPSESAFTRRAGGTFAP
Ixosc           EPPERQPKQEPTAALPDGSCS------------------------SSSSCS---------
                      .
```

Figure S7. Putative *Talitrus saltator* CLOCKWORK ORANGE (CWO) protein

Alignment of *Drosophila melanogaster* CWO (Drome-CWO; Accession no. AAF54527) with the *T. saltator* CWO (Tal-CWO) deduced from the Trinity *de novo* transcriptome assembly, together with the top two tblastn species homologue sequences *Metaseiulus occidentalis* uncharacterized (Metoc; Accession No. XM_003744642) and *Ixodes scapularis* conserved hypothetical protein (Ixosc; Accession No. XM_002400882). 5' and 3' sequence removed from all sequences except Tal-CWO. '*' indicates identical amino acid residues in the two proteins, '.' and ':' indicate similar amino acid residues between the two proteins. In this figure SMART identified domains of bHLH domain and Orange of the Hairy/E(SPL) family domain are highlighted in yellow and green respectively.
