# Supplementary material for: Identification and temporal expression of putative circadian clock transcripts in the amphipod crustacean Talitrus saltator
Source: PeerJ. 2016 Oct 5;4:e2555. doi: 10.7717/peerj.2555 (PMC5068443; doi:10.7717/peerj.2555)
Supplement: Figure S8 — Alignment of Drosophila melanogaster DCO (Drome-DCO; Accession No. AAF57110) with the T. saltator DBT (Tal-DBT) deduced from the Trinity de novo transcriptome assembly, together with the top two tblastn species homologue sequences Eurydice pulchra CKI-ε (Ep-CKI-ε ; Accession No. KC885972) and Ciona intestinalis CKI (Cioin-CKI; Accession No. XM_002125796). 5’ and 3’ sequence removed from all sequences except Tal-DBT. ’*’ indicates identical amino acid residues in the two proteins, ’.’ and ’:’ indicate similar amino acid residues between the two proteins. In this figure a SMART identified serine/threonine protein kinase catalytic domain is highlighted in yellow. [file peerj-04-2555-s008.pdf]

```
Drome-DCO       -MELRVGNKYRLGRKIGSGSFGDIYLGTTINTGEEVAIKLECIRTKHPQLHIESKFYKTM
Tal-DBT         -MELRVGNKYRLGRKIGSGSFGDIYLGTNISTAEEVAIKLECIKTKHPQLHIESKFYKMM
Ep-CKI-ε        MMELRVGNKYRLGRKIGSGSFGDIYLGTNISTGEEVAIKLECIKTKHPQLHIESKFYKMM
Cioin-CKI       -MELRVGNKYRLGRKIGSGSFGDIYLGTNISTGEEVAIKLECVKTKHPQLHIESKFYKMM
                 ***************************.*.*.*********::************** *

Drome-DCO       QGGIGIPRIIWCGSEGDYNVMVMELLGPSLEDLFNFCSRRFSLKTVLLLADQMISRIDYI
Tal-DBT         AGGVGIPAIKWCGSEGDYNVMVMELLGPSLEDLFNFCTRKFSLKTVLLLADQLITRIEYI
Ep-CKI-ε        AGGVGIPSIKWCGSEGDYNVMVMELLGPSLEDLFNFCSRKFFLKTVLLLADQLITRIEYI
Cioin-CKI       QGGVGIPTVKWCGAEGDYNVLVMELLGPSLEDLFNFCSRKFSLKTVLLLADQLISRIEFI
                 **:*** : ***:******:****************:*:* **********:*:**::*

Drome-DCO       HSRDFIHRDIKPDNFLMGLGKKGNLVYIIDFGLAKKFRDARSLKHIPYRENKNLTGTARY
Tal-DBT         HSKNFIHRDIKPDNFLMGLGKKGNLVYIIDFGLAKKYRDPRTHQHIPYRENKNLTGTARY
Ep-CKI-ε        HSKNFIHRDIKPDNFLMGLGKKGNLVYIIDFGLAKKYRDSRTHLHIPYRENKNLTGTARY
Cioin-CKI       HSKNFIHRDIKPDNFLMGLGKKGNLVYIIDFGLAKKYRDGRTHQHIPYRENKNLTGTARY
                **::********************************:** *:  ****************

Drome-DCO       ASINTHLGIEQSRRDDLESLGYVLMYFNLGALPWQGLKAANKRQKYERISEKKLSTSIVV
Tal-DBT         ASVNTHLGIEQSRRDDLESLGYILMYFNRGSLPWQGLKAATKRQKYERISEKKMQTRIED
Ep-CKI-ε        ASVNTHLGIEQSRRDDLESLGYVLMYFNRGSLPWQGLKAATKRQKYERISEKKMQTPIEE
Cioin-CKI       ASINTHLGIEQSRRDDLESLGYILMYFNLGTLPWQGLKAATKRQKYERISEKKMSTPIEL
                **:*******************:***** *:*********.************:.* *

Drome-DCO       LCKGFPSEFVNYLNFCRQMHFDQRPDYCHLRKLFRNLFHRLGFTYDYVFDWNLLKFGGPR
Tal-DBT         LCKGFPAEFATYLNVCRTLRFEEKPDYSHLRQLFRQLFHRQGFTYDYVFDWNLLKFVSEH
Ep-CKI-ε        LCKSFPNEFATYLNFCRSLRFEEKPDYSYLRQLFRQLFHRQGFTYDYVFDWNMLKFGGPR
Cioin-CKI       LCKGHPSEFATYLNYCRSLRFDDKPDYSYLRQLFRNLFHRQGFTYDYVFDWNMLKFGNRS
                ***..* **..*** ** ::*:::***.:**:***:**** ***********:*** .

Drome-DCO       NPQAIQQAQDGADG----------------------------------------------
Tal-DBT         PSSKQHQQQQQ-------------------------------------------------
Ep-CKI-ε        NQEEG-DRRDRGSNRPQGT-----------------------------------------
Cioin-CKI       STDDGKDHRTSASRGLVGSSATRQASTQKKLVVRQSSTMSKAKSKVSSWLTSSVVLHPKK
                  .   : :
```

Figure S8. Putative *Talitrus saltator* DOUBLTETIME (DBT) protein

Alignment of *Drosophila melanogaster* DCO (Drome-DCO; Accession No. AAF57110) with the *T. saltator* DBT (Tal-DBT) deduced from the Trinity *de novo* transcriptome assembly, together with the top two tblastn species homologue sequences *Eurydice pulchra* CKI-ε (Ep-CKI-ε; Accession No. KC885972) and *Ciona intestinalis* CKI (Cioin-CKI; Accession No. XM_002125796). 5' and 3' sequence removed from all sequences except Tal-DBT. '*' indicates identical amino acid residues in the two proteins, '.' and ':' indicate similar amino acid residues between the two proteins. In this figure a SMART identified serine/threonine protein kinase catalytic domain is highlighted in yellow.
