# Supplementary material for: Identification and temporal expression of putative circadian clock transcripts in the amphipod crustacean Talitrus saltator
Source: PeerJ. 2016 Oct 5;4:e2555. doi: 10.7717/peerj.2555 (PMC5068443; doi:10.7717/peerj.2555)
Supplement: Figure S9 — Alignment of Drosophila melanogaster PDP1ε (Drome-PDP1-ε; Accession No. AAF04509) with the T. saltator PDP1ε (Tal-PDP1-ε ) deduced from the Trinity de novo transcriptome assembly, together with the top two tblastn species homologue sequences Atta cephalotes TEF (Attce-TEF; Accession No. XM_012203776) and Branchiostoma floridae hypothetical protein (Brafl; Accession No. XM_002601463). ’*’ indicates identical amino acid residues in the two proteins, ’.’ and ’:’ indicate similar amino acid residues between the two proteins. In this figure a SMART identified basic region leucin zipper domain is highlighted in green. [file peerj-04-2555-s009.pdf]

```
Drome-PDP1-ε      ------------------------------------------------------------
Tal-PDP1-ε        MDTLYSGKNCASLSQMADSTFPSFDGGTTEGAFRASPSCASKDRDGTYPSGAVTLLGSNL
Attce-TEF         ------------------------------------------------------------
Brafl             ------------------------------------------------------------

Drome-PDP1-ε      ---------------------------MDPQSNAAAAAQLLXPVAMLPLLQRCSCKWQPG
Tal-PDP1-ε        WNKTIPYDPDLKVALEFMDLEEFLNENSIPVDDSTRSSNVLPENSDVEASHNCG-RVTTT
Attce-TEF         ------------------------------MSDKDRSSPTLVENSLKSLLD------QP-
Brafl             ------------------------------------------------------------

Drome-PDP1-ε      IEWECQWNGNAVVAATGNGNGGNPGQNNNNNNGNNSGNSNNNSNNNVSSVQHVANAVAAA
Tal-PDP1-ε        VADSCSFASLTTTAGASSASSSPLRLNLQTHPGYSPASLHDSNSSTYFPPQSELNFMNSD
Attce-TEF         ----------SIFPLIGNVNG---------------------------------------
Brafl             ------------------------------------------------------------

Drome-PDP1-ε      ----VIANEHHNHLNSLKARFQPASSGKSTSNSKEIIC---PDDKYKE---EGDIW-NVE
Tal-PDP1-ε        SKLLLDDSDEAPDSHSMPGGRRSPSPENLTSLSSSLLRSMNADDKTVMRSVNSTISSTSE
Attce-TEF         ---------------------------KSLSNGKEILCKDGPEEKKDA---DGELWGNVE
Brafl             ------------------------------------------------------------

Drome-PDP1-ε      AQTAFLG---------PNLWDKTLPYD---AD-----------LKVT-QYADLD------
Tal-PDP1-ε        VSNASSSQPFGKASEFPNFDDYSVDFHGVTADQSSGGGENPSMLKIDQTLRDGNSEPHQK
Attce-TEF         AQAAFLG---------PNLWDKTLPYD---AD-----------LKVLNHYVDLD------
Brafl             ------------------------------------------------------------

Drome-PDP1-ε      --------------EFLSENNIP-DGLPGTHLGHSSG--LG--HRSDSLGHAAGLSLGLG
Tal-PDP1-ε        IDESCASEITSGSWESLRASSQRCDTTSSASTVETTGQERGR-RKNQGRGSAKPQSTGVR
Attce-TEF         --------------EFLSENGIPVDGVAGGGQGTMQGSQLHKINNTEAAGHQGPAGLHLE
Brafl             ----------------------------------------------------SPPHISME
                                                                            :

Drome-PDP1-ε      HITTKRERSPSPSD-----------CISPDTLNPPSPAESTFSFAS-SGRDFDPRTRAFS
Tal-PDP1-ε        HRRRRGITSYAEIDDDMISELCDFTTDKPEQVDDDRITPSQHNPNSKTGEKFNPRTRKFS
Attce-TEF         PVT-KRERSPSPSE-----------CCSPDTLNPPSPADSTLSMAS-SGRDFDPRTRAFS
Brafl             EVI-------------------------PEVEYDVSPTDVALASIP-GKEEFNPRKRAFS
                                              *:       :       .   ..*:**.* **

Drome-PDP1-ε      DEELKPQPMIKKSRKQFVPDELKDDKYWARRRKNNIAAKRSRDARRQKENQIAMRARYLE
Tal-PDP1-ε        EDELKPQPMVKKSRKQYVPPDLKDDKYWARRQKNNVAAKRSRDARRVKENQIALRASFLE
Attce-TEF         DEELKPQPMIKKSRKQFVPDDLKDDKYWARRRKNNMAAKRSRDARRMKENQIALRAGFLE
Brafl             EEELRPQPMIKKSRKIFVPEDLKDDKYWERRKKNNVAAKRSRDARRIKENQVALRASFLE
                  ::**:****:***** :** :******* **:***:********** ****:*:** :**

Drome-PDP1-ε      KENATLHQEVEQLKQENMDLRARLSKFQDV---
Tal-PDP1-ε        EMHSKLRKQLDEANDTISDLRKRLSKYEAV---
Attce-TEF         KENMGLRQELDRLKNENMLLRDKLSKYTDV---
Brafl             KENATLKEELLKAKEENVILSKKLLKYEQQNRV
                  : :  *:::: . ::    *  :* *:
```

Figure S9. Putative *Talitrus saltator* Par Domain Protein 1ε (PDP1ε) protein

Alignment of *Drosophila melanogaster* PDP1ε (Drome-PDP1-ε; Accession No. AAF04509) with the *T. saltator* PDP1ε (Tal-PDP1-ε) deduced from the Trinity *de novo* transcriptome assembly, together with the top two tblastn species homologue sequences *Atta cephalotes* TEF (Attce-TEF; Accession No. XM_012203776) and *Branchiostoma floridae* hypothetical protein (Brafl; Accession No. XM_002601463). '*' indicates identical amino acid residues in

the two proteins, '.' and ':' indicate similar amino acid residues between the two proteins. In this figure a SMART identified basic region leucin zipper domain is highlighted in green.
