# Supplementary material for: Identification and temporal expression of putative circadian clock transcripts in the amphipod crustacean Talitrus saltator
Source: PeerJ. 2016 Oct 5;4:e2555. doi: 10.7717/peerj.2555 (PMC5068443; doi:10.7717/peerj.2555)
Supplement: Figure S10 — Alignment of Drosophila melanogaster PP1 (Drome-PP1; Accession No. CAA39820) with the T. saltator PP1 (Tal-PP1) deduced from the Trinity de novo transcriptome assembly, together with the top two tblastn species homologue sequences Acromyrmex echinatior PPα2 (Acrec-PPα2; Accesion No. XM_011065955) and Vollenhovia emeryi PPα2 (Volem-PPα2; Accession No. XM_012019400). ’*’ indicates identical amino acid residues in the two proteins, ’.’ and ’:’ indicate similar amino acid residues between the two proteins. In this figure a SMART identified protein phosphatase 2Ac catalytic domains of the serine/threonine phosphatase family is highlighted in yellow. [file peerj-04-2555-s010.pdf]

```
Drome-PP1       -------MSDIMNIDSIISR-----------LLEVRGARPGK------------NVQLSE
Tal-PP1         MSSNANTTTPKLSTKERVVKSVPFPPSHKLTCAEVFDPKTGKPKFEWLKNHFILEGRIEE
Acrec-PPα2      -----MAETDKLNIDNIIAR-----------LLEVRGARPGK------------NVQLTE
Volem-PPα2      -----MAETDKLNIDNIIAR-----------LLEVRGARPGK------------NVQLTE
                        :  :. .. : :             ** ..:.**            : :: *

Drome-PP1       SEIRSLCLKSREIFLSQPILLELEAPLKICGDIHGQYYDLLRLFEYGGFPPESNYLFLGD
Tal-PP1         AAALRIINEGAALLRQEKTMIDIEAPVTVCGDIHGQYYDLLRLFEYGGFPPESNYLFLGD
Acrec-PPα2      VEIRGLCLKSREIFLSQPILLELEAPLKICGDIHGQYYDLLRLFEYGGFPPESNYLFLGD
Volem-PPα2      VEIRGLCLKSREIFLSQPILLELEAPLKICGDIHGQYYDLLRLFEYGGFPPESNYLFLGD
                     :  :.  :: .:  ::::***:.:*******************************

Drome-PP1       YVDRGKQSLETICLLLAYKIKYAENFFLLRGNHECASINRIYGFYDECKRRYTIKLWKTF
Tal-PP1         YVDRGKQSLETICLLLAYKIKYPENFFLLRGNHECASINRIYGFYDECKRRYNIKLWKTF
Acrec-PPα2      YVDRGKQSLETICLLLAYKIKYPENFFLLRGNHECASINRIYGFYDECKRRYNIKLWKTF
Volem-PPα2      YVDRGKQSLETICLLLAYKIKYPENFFLLRGNHECASINRIYGFYDECKRRYNIKLWKTF
                *********************.******************************.*******

Drome-PP1       TDCFNCLPVAAIVDEKIFCCHGGLSPDLSSMEQIRRIMRPTDVPDQGLLCDLLWSDPDKD
Tal-PP1         TDCFNCLPVAAIVDEKIFCCHGGLSPDLQSMEQIRRIMRPTDVPDQGLLCDLLWSDPDKD
Acrec-PPα2      TDCFNCLPVAAIVDEKIFCCHGGLSPDLQSMEQIRRIMRPTDVPDQGLLCDLLWSDPDKD
Volem-PPα2      TDCFNCLPVAAIVDEKIFCCHGGLSPDLQSMEQIRRIMRPTDVPDQGLLCDLLWSDPDKD
                ****************************.*******************************

Drome-PP1       TMGWGENDRGVSFTFGAEVVGKFLQKHEFDLICRAHQVVEDGYEFFAKRQLVTLFSAPNY
Tal-PP1         TMGWGENDRGVSFTFGAEVVAKFLHKHDFDLICRAHQVVEDGYEFFAKRQLVTLFSAPNY
Acrec-PPα2      TMGWGENDRGVSFTFGAEVVAKFLHKHDFDLICRAHQVVEDGYEFFAKRQLVTLFSAPNY
Volem-PPα2      TMGWGENDRGVSFTFGAEVVAKFLHKHDFDLICRAHQVVEDGYEFFAKRQLVTLFSAPNY
                ********************.***:**:********************************

Drome-PP1       CGEFDNAGAMMSVDDTLMCSFQILKPADKRRFVYPNFGSSGRPLTPPRGANNKNKKK-
Tal-PP1         CGEFDNAGAMMSVDETLMCSFQILKPADKKKFSYVSLN-SGRPVTPPRGAANQKPKKK
Acrec-PPα2      CGEFDNAGAMMSVDETLMCSFQILKPADKRKFTYGGLN-AGRPVTPPRGANNKNKKK-
Volem-PPα2      CGEFDNAGAMMSVDETLMCSFQILKPADKRKFTYGGLN-AGRPVTPPRGANNKNKKK-
                **************:**************::* * .:. :***:****** *:: **
```

Figure S10. Putative *Talitrus saltator* Protein phosphatase 1 (PP1) protein.

Alignment of *Drosophila melanogaster* PP1 (Drome-PP1; Accession No. CAA39820) with the *T. saltator* PP1 (Tal-PP1) deduced from the Trinity *de novo* transcriptome assembly, together with the top two tblastn species homologue sequences *Acromyrmex echinatior* PPα2 (Acrec-PPα2; Accesion No. XM_011065955) and *Vollenhovia emeryi* PPα2 (Volem-PPα2; Accession No. XM_012019400). '*' indicates identical amino acid residues in the two proteins, '.' and ':' indicate similar amino acid residues between the two proteins. In this figure a SMART identified protein phosphatase 2Ac catalytic domains of the serine/threonine phosphatase family is highlighted in yellow.
