# Supplementary material for: Identification and temporal expression of putative circadian clock transcripts in the amphipod crustacean Talitrus saltator
Source: PeerJ. 2016 Oct 5;4:e2555. doi: 10.7717/peerj.2555 (PMC5068443; doi:10.7717/peerj.2555)
Supplement: Figure S11 — Alignment of Drosophila melanogaster MTS (Drome-MTS; Accession no. AAF52567) with the T. saltator MTS (Tal-MTS) deduced from the Trinity de novo transcriptome assembly, together with the top two tblastn species homologue sequences Pediculus humanus corporis PP-V (Pedhu-PP-V; Accession No. XM_002426681) and Nasonia vitripennis PPIIA (Nasvi-PPIIA; Accession No. XM_001602456). ’*’ indicates identical amino acid residues in the two proteins, ’.’ and ’:’ indicate similar amino acid residues between the two proteins. In this figure SMART identified protein phosphatase 2Ac catalytic domains of the serine/threonine phosphatase family are highlighted in green. [file peerj-04-2555-s011.pdf]

```
Drome-MTS       MEDKATTKDLDQWIEQLNECNQLTETQVRTLCDKAKEILSKESNVQEVKCPVTVCGDVHG
Tal-MTS         MEEKTQIKELDQWIEQLMECKQLGENQVKTLCEKAKEVLAKESNVQEVKSPVTVCGDVHG
Pedhu-PP-V      MEEKTSLKELDQWIEQLNECKQLTESQVKTLCDKAKEILAKESNVQEVKCPVTVCGDVHG
Nasvi-PPIIA     MEEKASLKELDQWIEQLNDCKQLTESQVKTLCDKAKEILAKESNVQEVKSPVTVCGDVHG
                **:*:  *:******** :*:** *.**:***:****:*:*********.**********

Drome-MTS       QFHDLMELFRIGGKSPDTNYLFMGDYVDRGYYSVETVTLLVALKVRYRERITILRGNHES
Tal-MTS         QFHDLMELFKIGGRSPDTNYLFMGDYVDRGYYSVETVTLLVTLKVRFRERITILRGNHES
Pedhu-PP-V      QFHDLMELFRIGGKSPDTNYLFMGDYVDRGYYSVETVTLLVALKVRYRERITILRGNHES
Nasvi-PPIIA     QFHDLMELFRIGGKSPDTNYLFMGDYVDRGYYSVETVTLLVALKVRYRERITILRGNHES
                *********:***:***************************:****:*************

Drome-MTS       RQITQVYGFYDECLRKYGNANVWKYFTDLFDYLPLTALVDGQIFCLHGGLSPSIDSLDHI
Tal-MTS         RQITQVYGFYDECLRKYGNANVWKYFTDLFDYLPLTALVDNQIFCLHGGLSPSIDTLDHI
Pedhu-PP-V      RQITQVYGFYDECLRKYGNANVWKFFTDLFDYLPLTALVDGQIFCLHGGLSPSIDTLDHI
Nasvi-PPIIA     RQITQVYGFYDECLRKYGNANVWKFFTDLFDYLPLTALVDGQIFCLHGGLSPSIDTLDHI
                ************************:***************.**************:****

Drome-MTS       RALDRLQEVPHEGPMCDLLWSDPDDRGGWGISPRGAGYTFGQDISETFNNTNGLTLVSRA
Tal-MTS         RALDRLQEVPHEGPMCDLLWSDPDDRGGWGISPRGAGYTFGQDISETFNHSNGLTLVSRA
Pedhu-PP-V      RALDRLQEVPHEGPMCDLLWSDPDDRGGWGISPRGAGYTFGQDISETFNHSNGLTLVSRA
Nasvi-PPIIA     RALDRLQEVPHEGPMCDLLWSDPDDRGGWGISPRGAGYTFGQDISETFNHSNGLTLVSRA
                *************************************************::*********

Drome-MTS       HQLVMEGYNWCHDRNVVTIFSAPNYCYRCGNQAALMELDDSLKFSFLQFDPAPRRGEPHV
Tal-MTS         HQLVMEGYNWCHERNVVTIFSAPNYCYRCGNQAAIMELDDSLKYSFLQFDPAPRRGEPHV
Pedhu-PP-V      HQLVMEGYNWCHDRNVVTIFSAPNYCYRCGNQAAIMELDDALKYSFLQFDPAPRRGEPHV
Nasvi-PPIIA     HQLVMEGYNWCHDRNVVTIFSAPNYCYRCGNQAAIMELDDALKYSFLQFDPAPRRGEPHV
                ************:*********************:*****:**:****************

Drome-MTS       TRRTPDYFL
Tal-MTS         TRRTPDYFL
Pedhu-PP-V      TRRTPDYFL
Nasvi-PPIIA     TRRTPDYFL
                *********
```

Figure S11. Putative *Talitrus saltator* MICROTUBULE STAR (MTS) protein

Alignment of *Drosophila melanogaster* MTS (Drome-MTS; Accession no. AAF52567) with the *T. saltator* MTS (Tal-MTS) deduced from the Trinity *de novo* transcriptome assembly, together with the top two tblastn species homologue sequences *Pediculus humanus corporis* PP-V (Pedhu-PP-V; Accession No. XM_002426681) and *Nasonia vitripennis* PPIIA (Nasvi-PPIIA; Accession No. XM_001602456). '*' indicates identical amino acid residues in the two proteins, '.' and ':' indicate similar amino acid residues between the two proteins. In this figure SMART identified protein phosphatase 2Ac catalytic domains of the serine/threonine phosphatase family are highlighted in green.
