# Supplementary material for: Identification and temporal expression of putative circadian clock transcripts in the amphipod crustacean Talitrus saltator
Source: PeerJ. 2016 Oct 5;4:e2555. doi: 10.7717/peerj.2555 (PMC5068443; doi:10.7717/peerj.2555)
Supplement: Figure S12 — Alignment of Drosophila melanogaster WBT (Drome-WBT; Accession No. AAF56720) with the T. saltator WBT (Tal-WBT) deduced from the Trinity de novo transcriptome assembly, together with the top two tblastn species homologue sequences Tribolium castaneum PPIIA (Trica-PPIIA; Accession No. XM_008197190) and Bombyx mori PPIIA (Bommo-PPIIA; Accession No. XM_004924466). ’*’ indicates identical amino acid residues in the two proteins, ’.’ and ’:’ indicate similar amino acid residues between the two proteins. In this figure a SMART identified B56 domain is highlighted in yellow. [file peerj-04-2555-s012.pdf]

```
Drome-WBT        MSSG--TFVDRIDPFAKRSLKKKGKKSQGSSRYRNSQDVELQQLPPLKADCSSLEQEELF
Tal-WBT          MSAGSGNFVDRIDPFAKRSLKKKPKRSQGSSRYRTANDVDLQPLPLLK-DVPGGEQEDLF
Trica-PPIIA      MSTT--AFVDRIDPFAKRSLKKKTKKSQGSSRYRNSQDVELQPLPLLK-DVPASEQEELF
Bommo-PPIIA      MSSG--TFVDRIDPFAKRSLKKKTKKSQGSSRYRNTQDVELQALPLLK-DVPSSEQEELF
                 **:    **************** *:********.::**:** ** ** * .. ***:**

Drome-WBT        IRKLRQCCVSFDFMDPVTDLKGKEIKRAALNDLSTYITHGRGVLTEPVYPEIIRMISCNL
Tal-WBT          LRKLRQCCVGFDFLDPVADLKGKETKRSTLNELVDYITAGRGVLTEPVYPEIIAMIACNL
Trica-PPIIA      IRKLRQCCVPFDFMDPVADLKGKEVKRCTLNELVDYITGGRGVLTEPVYPEIIKMISANL
Bommo-PPIIA      IRKLRQCCVAFDFMDPVADLKGKEIKRATLNELVEYIHGGRGVLTEPVYPEIIKMISANL
                 :******** ***:***:****** **.:**:*  **  ************** **:.**

Drome-WBT        FRTLPPSENPDFDPEEDDPTLEASWPHLQLVYEVFLRFLESQDFQATIGKRVIDQKFVLQ
Tal-WBT          FRTLPPSDNPDFDPEEDDPTLEASWPHLQLVYEFFLRFLESPDFQPAIGKKVIDQKFVLQ
Trica-PPIIA      FRTLPPSENPDFDPEEDDPTLEASWPHLQLVYEFFLRFLESSDFQPAIGKRVIDQKFVLQ
Bommo-PPIIA      FRTLPPSENPDFDPEEDDPTLEASWPHLQLVYEFFLRFLESTDFQPTIGKKVIDQKFVLQ
                 *******:*************************.******* ***.:***:*********

Drome-WBT        LLELFDSEDPRERDFLKTVLHRIYGKFLGLRAFIRKQINNIFLRFIYETEHFNGVGELLE
Tal-WBT          LLELFDSEDPRERDFLKTVLHRIYGKFLGLRAFIRKQINNIFLRFIYETEHFNGVGELLE
Trica-PPIIA      LLELFDSEDPRERDFLKTVLHRIYGKFLGLRAFIRKQINNIFLRFVYETEHFNGVGELLE
Bommo-PPIIA      LLDLFDSEDPRERDFLKTVLHRIYGKFLGLRAFIRKQINNIFLRFVYETEHFNGVGELLE
                 **:******************************************:**************

Drome-WBT        ILGSIINGFALPLKAEHKQFLVKVLLPLHKVKCLSLYHAQLAYCIVQFLEKDPFLTEPVV
Tal-WBT          ILGSIINGFALPLKAEHKQFLIKVLIPLHKAKCLSLYHAQLAYCVVQFLEKDPTLTEPVI
Trica-PPIIA      ILGSIINGFALPLKAEHKQFLVKVLIPLHKVKCLSLYHAQLAYCVVQFLEKDPTLTEPVV
Bommo-PPIIA      ILGSIINGFALPLKSEHKQFLVKVLLPLHKVKCLSLYHAQLAYCVVQFLEKDATLTEPVV
                 **************:******:***:****.*************:*******. *****:

Drome-WBT        RGLLKFWPKTCSQKEVMFLGEIEEILDVIDPPQFVKIQEPLFRQIAKCVSSPHFQVAERA
Tal-WBT          KGLLKIWPKTCSQKEVMFLSEIEEILDVIEPNQFGKVQEPLFKQISKCVSSPHFQVAERA
Trica-PPIIA      RGLLKYWPKTCSQKEVMFLGEIEEVLDVIEPSQFTRIQEPLFRQIAKCVSSPHFQVAERA
Bommo-PPIIA      RGLLKFWPKTCSQKEVMFLGEIEEILDVIEPAQFAKIQEPLFRQIAKCVSSPHFQVAERA
                 :**** *************.****:****:* ** ::*****:**:**************

Drome-WBT        LYLWNNEYAMSLIEENNAVIMPIMFPALYRISKEHWNQTIVALVYNVLKTFMEMNSKLFD
Tal-WBT          LYFWNNEYIMSLIEENSNVILPIMFPALYRISKEHWNQTIVALVYNVLKTFMEMNSKLFD
Trica-PPIIA      LYFWNNEYIMSLMEENNHVIMPIMFPALYRISKEHWNQTIVALVYNVLKTFMEMNSKLFD
Bommo-PPIIA      LYFWNNEYIMSLMEENNHVIMPIMFPALYRISKEHWNQTIVALVYNVLKTFMEMNSKLFD
                 **:***** ***:***. **:***************************************

Drome-WBT        ELTSSYKAERQKEKKRERDREELWKKLHELESNRSSGRTAGGSATTSNSAASAASTSLQP
Tal-WBT          ELTASYKSERQREKKRDREREELWKKLQALELNRTQ--------PKE-------------
Trica-PPIIA      ELTVSYKAERQKEKKREKERDELWKKLAELELNHNKNLPAQNNSPPSKK-----------
Bommo-PPIIA      ELTASYKAERQKEKKREKERDELWKRLGELEISHKR---QQHAAPLAK------------
                 *** ***:***:****:::*:****:*  ** .:.         .

Drome-WBT        PSSAGLNSHQQQSNSGSSGSLSSGGAGGDNNPATTNAKIKQDKADN
Tal-WBT          ----------------------------------------------
Trica-PPIIA      ----------------------------------------------
Bommo-PPIIA      ----------------------------------------------
```

Figure S12. Putative *Talitrus saltator* WIDERBORST (WBT) protein

Alignment of *Drosophila melanogaster* WBT (Drome-WBT; Accession No. AAF56720) with the *T. saltator* WBT (Tal-WBT) deduced from the Trinity *de novo* transcriptome assembly, together with the top two tblastn species homologue sequences *Tribolium castaneum* PPIIA (Trica-PPIIA; Accession No. XM_008197190) and *Bombyx mori* PPIIA (Bommo-PPIIA; Accession No. XM_004924466). '*' indicates identical amino acid residues in the two

proteins, '.' and ':' indicate similar amino acid residues between the two proteins. In this figure a SMART identified B56 domain is highlighted in yellow.
