# Supplementary material for: Identification and temporal expression of putative circadian clock transcripts in the amphipod crustacean Talitrus saltator
Source: PeerJ. 2016 Oct 5;4:e2555. doi: 10.7717/peerj.2555 (PMC5068443; doi:10.7717/peerj.2555)
Supplement: Figure S13 — Alignment of Drosophila melanogaster (Drome-TWS; Accession no. AAF54498) with the T. saltator TWS (Tal-TWS) deduced from the Trinity de novo transcriptome assembly, together with the top two tblastn species homologue sequences Scylla paramamosain PPIIA (Scypa-PPIIA; Accession No. JQ867383) and Megachile rotundata PPIIA (Megro-PPIIA; Accession No. XM_012288516). ’*’ indicates identical amino acid residues in the two proteins, ’.’ and ’:’ indicate similar amino acid residues between the two proteins. In this figure seven SMART identified WD40 domains are highlighted in green. [file peerj-04-2555-s013.pdf]

```
Drome-TWS        MGRWGRQSPVLEPPDPQMQTTPPPPTLPPRTFMRQSSITKIGNMLNTAININGAKKPASN
Tal-TWS          --------------------------------------------------------MAGN
Scypa-PPIIA      --------------------------------------------------------MAGN
Megro-PPIIA      --------------------------------------------------------MAGN
                                                                          *.*

Drome-TWS        G-EASWCFSQIKGALDDDVTDADIISCVEFNHDGELLATGDKGGRVVIFQRDPASKAANP
Tal-TWS          GSDTQWCFSQVKGTLDDEITDADVISCVEFSHDGDLLATGDKGGRVVIFQRDPLSKGCSP
Scypa-PPIIA      G-DIQWCFSQVKGTLDDDVSEADIISCVEFNHDGDLLATGDKGGRVVIFQRDPSSKNCHP
Megro-PPIIA      G-DIQWCFSQVKGTLEDDVTEADIISCVEFNHDGDLLATGDKGGRVVIFQRDPISKNSIP
                 * : .*****:**:*:*::::**:******.***:****************** ** . *

Drome-TWS        RRGEYNVYSTFQSHEPEFDYLKSLEIEEKINKIRWLQQKNPVHFLLSTNDKTVKLWKVSE
Tal-TWS          TRGEYNVYSTFQSHEPEFDYLKSLEIEEKINKIRWLKRKNPAHFLLSTNDKTIKLWKVSE
Scypa-PPIIA      RRGEYNVYSTFQSHEPEFDYLKSLEIEEKINKIRWLKRKNPAHFLLSTNDKTIKLWKVSE
Megro-PPIIA      RRGEYNVYSTFQSHEPEFDYLKSLEIEEKINKIRWLKRKNPAHFLLSTNDKTIKLWKVSE
                  ***********************************::***.**********:*******

Drome-TWS        RDKSFGGYNTKEENGLIRDPQNVTALRVPSVKQIPLLVEASPRRTFANAHTYHINSISVN
Tal-TWS          RDKRAEGYNLRDDSGQIRDSSSITSLRVPTLKQMELMVEASPRRIFANAHTYHINSISIN
Scypa-PPIIA      RDKRAEGYNLRDESGQIRDPTSLTALRVPVLKPMELMVEASPRRIFANAHTYHINSISIN
Megro-PPIIA      RDKRVEGYNTKEENGTIRDPACITSLRVPTIKPMELMVEASPRRIFANAHTYHINSISVN
                 ***   *** :::.* ***.  :*:**** :* : *:******* *************:*

Drome-TWS        SDQETFLSADDLRINLWHLEVVNQSYNIVDIKPTNMEELTEVITAAEFHPTECNVFVYSS
Tal-TWS          SDQETYLSADDLRINLWHLEVTDQSFNIVDIKPSNMEELTEVITAAEFHPRDCNAFVYSS
Scypa-PPIIA      SDQETYLSADDLRINLWHMEVTDQSFNIVDIKPTNMEELTEVITAAEFHPHDCNVFVYSS
Megro-PPIIA      SDQETYLSADDLRINLWHLEITDQSFNIVDIKPTNMEELTEVITAAEFHPAECNVLVYSS
                 *****:************:*:.:**:*******:**************** :**.:****

Drome-TWS        SKGTIRLCDMRSAALCDRHSKQFEEPENPTNRSFFSEIISSISDVKLSNSGRYMISRDYL
Tal-TWS          SKGTIRLCDMRAAALCDSHAKMFEEAEDPSNRSFFSEIISSISDVKFSNSGSLMISRDYL
Scypa-PPIIA      SKGTIRLCDMRQAALCDSHSKLFEEPEDPTNRSFFSEIISSISDVKFSNSGRYMISRDYL
Megro-PPIIA      SKGTIRLCDMRSAALCDQHSKLFEEPEDPTNRSFFSEIISSISDVKLSNSGRYMISRDYL
                 *********** ***** *:* ***.*:*:****************:****  *******

Drome-TWS        SIKVWDLHMETKPIETYPVHEYLRAKLCSLYENDCIFDKFECCWNGKDSSIMTGSYNNFF
Tal-TWS          TIKVWDLRKENQPLETYSVHDYLRSKLCSLYENDCIFDKFECCWSGNDKHIMTGSYNNFF
Scypa-PPIIA      SVKVWDLHMETKPIETYPVHEYLRPKLCSLYENDCIFDKFECCWSGNDSAIMTGSYNNFF
Megro-PPIIA      SVKVWDLQMETKPIECYPVHEYLRSKLCSLYENDCIFDKFECCWSGNDSAIMTGSYNNFF
                 ::*****: *.:*:* *.**:***.*******************.*:*. **********

Drome-TWS        RVFDRNSKKDVTLEASRDIIKPKTVLKPRKVCTGG-KRKKDEISVDCLDFNKKILHTAWH
Tal-TWS          RMFDRENKKDVTLEAAREIAKPRTVLKPRKIGGSGSKRKKDEINVDCLDFSKKILHTAWH
Scypa-PPIIA      RMFDRTSKRDVTLEASRETAKPRYLLKPRKVCTAG-KRKKDEISVDCLDFNKKILHTAWH
Megro-PPIIA      RVFDRTTKRDLTLEAARDIAKPKTLLKPRKVCTGG-KRKKDEISVDCLDFNKKILHTAWH
                 *:*** .*:*:****:*:  **: :*****:  .* *******.******.*********

Drome-TWS        PEENIIAVAATNNLFIFQDKF
Tal-TWS          PTENVIAVAATNNLYIFQDKQ
Scypa-PPIIA      PHENIIAVAATNNLYIFQDKF
Megro-PPIIA      PSENVVAVAATNNLFLFQDKL
                 * **::********::****
```

Figure S13. Putative *Talitrus saltator* TWINS (TWS) protein

Alignment of *Drosophila melanogaster* (Drome-TWS; Accession no. AAF54498) with the *T. saltator* TWS (Tal-TWS) deduced from the Trinity *de novo* transcriptome assembly, together with the top two tblastn species homologue sequences *Scylla paramamosain* PPIIA (Scypa-PPIIA; Accession No. JQ867383) and *Megachile rotundata* PPIIA (Megro-PPIIA; Accession No. XM_012288516). '*' indicates identical amino acid residues in the two proteins, '.' and ':'

indicate similar amino acid residues between the two proteins. In this figure seven SMART identified WD40 domains are highlighted in green.
