# Supplementary material for: Identification and temporal expression of putative circadian clock transcripts in the amphipod crustacean Talitrus saltator
Source: PeerJ. 2016 Oct 5;4:e2555. doi: 10.7717/peerj.2555 (PMC5068443; doi:10.7717/peerj.2555)
Supplement: Figure S14 — Alignment of Drosophila melanogaster SGG (Drome-SGG; Accession no. AAN09084) with the T. saltator SGG (Tal-SGG) deduced from the Trinity de novo transcriptome assembly, together with the top two tblastn species homologue sequences Athalia rosae GSK-III (Athro-GSK-III; Accession No. XM_012400594) and Linepithema humile GSK-III (Linhu-GSK-III; Accession No. XM_012372547). ’*’ indicates identical amino acid residues in the two proteins, ’.’ and ’:’ indicate similar amino acid residues between the two proteins. In this figure a SMART identified serine/threonine protein kinase catalytic domain is highlighted in yellow. [file peerj-04-2555-s014.pdf]

```
Drome-SGG           MSGRPRTSSFAEGNK-QSPSLVLGGVKTCSRDGSKITTVVATPGQGTDRVQEVSYTDTKV
Tal-SGG             MSGRPRTTSFAEGNKGPLAAVNFPGMKISSKDGNKVTTVVATAGLGSDRPQEVSYMDTKV
Athro-GSK-III       MSGRPRTTSFAEGNK-PPTNPPLGGMKISSKDGSKVTTVVASAGAGPDRPQEVGYTDTKV
Linhu-GSK-III       MSGRPRTTSFAEGNK-PPANPPLGGMKISSKDGSKVTTVVATPGAGPDRPQEVAYTDTKV
                    *******:*******   .   : *:* .*:**.*:*****:.* *.** ***.* ****

Drome-SGG           IGNGSFGVVFQAKLCDTGELVAIKKVLQDRRFKNRELQIMRKLEHCNIVKLLYFFYSSGE
Tal-SGG             IGNGSFGVVFQAKLVESGELVAIKKVLQDKRFKNRELQIMRRLEHCNIVELKYFFYSCGD
Athro-GSK-III       IGNGSFGVVYQAKLCDTGEMVAIKKVLQDKRFKNRELQIMRRLEHCNIVKLKYFFYSSGD
Linhu-GSK-III       IGNGSFGVVYQAKLCDSGEMVAIKKVLQDKRFKNRELQIMRRLEHCNIVKLKYFFYSSGD
                    *********:**** ::**:*********:***********:*******:* *****.*:

Drome-SGG           KRDEVFLNLVLEYIPETVYKVARQYAKTKQTIPINFIRLYMYQLFRSLAYIHSLGICHRD
Tal-SGG             KKDEVFLNLVLEYVPETIYKVARHHGKQKQTIPMSYIKLYMYQLFRSLAYMHALGVCHRD
Athro-GSK-III       KKDEVYLNLVLEYIPETVYKVARHYSKSKQTIPISFIKLYMYQLFRSLAYIHSLGICHRD
Linhu-GSK-III       KKDEVYLNLVLEYIPETVYKVARHYSKSKQTIPISFIKLYMYQLFRSLAYIHSLGICHRD
                    *:***:*******:***:*****::.* *****:.:*:************:*:**:****

Drome-SGG           IKPQNLLLDPETAVLKLCDFGSAKQLLHGEPNVSYICSRYYRAPELIFGAINYTTKIDVW
Tal-SGG             IKPQNLLVDPETGVLKLCDFGSAKHLVRGEPNVSYICSRYYRAPELIFGATDYTTNIDVW
Athro-GSK-III       IKPQNLLLDPETGVLKLCDFGSAKHLVKGEPNVSYICSRYYRAPELIFGAIDYTTKIDVW
Linhu-GSK-III       IKPQNLLLDPDTGVLKLCDFGSAKHLVKGEPNVSYICSRYYRAPELIFGAIDYTTKIDVW
                    *******:**:*.***********:*::********************** :***:****

Drome-SGG           SAGCVLAELLLGQPIFPGDSGVDQLVEVIKVLGTPTREQIREMNPNYTEFKFPQIKSHPW
Tal-SGG             SAGCVLSELLLAQPIFPGDSGVDQLVEIIKVLGTPTREQIREMNPNYTEFKFPQIKSHPW
Athro-GSK-III       SAGCVLAELLLGQPIFPGDSGVDQLVEIIKVLGTPTREQIREMNPNYTEFKFPQIKSHPW
Linhu-GSK-III       SAGCVLAELLLGQPIFPGDSGVDQLVEIIKVLGTPTRDQIREMNPNYTEFKFPQIKSHPW
                    ******:****.***************:*********:**********************

Drome-SGG           QKVFRIRTPTEAINLVSLLLEYTPSARITPLKACAHPFFDELRMEGNHTLPNGRDMPPLF
Tal-SGG             QKVFRQRTPEDAINLVSRLLEYTPSARISPLQACTHRFFDELREPN-TRLPNNRPLPPLF
Athro-GSK-III       QKVFRARTPPEAMELVARLLEYTPSLRWTPLQACAHSFFNELREQG-TRLPNGRELPPLF
Linhu-GSK-III       QKVFRARTPPEAMDLVARLLEYTPSLRMTPLQACAHSFFNELREQG-TRLPSGRELPPLF
                    ***** *** :*::**: ******* * :**:**:* **:***  .   **..* :****

Drome-SGG           NFTEHELSIQPSLVPQLLPKHLQNASGPGGNRPSAGGAASIAASGSTSVSSTGSGASVEG
Tal-SGG             NFTELELKIQPELASKLIPSHYQS------------------------------------
Athro-GSK-III       NFTEQELQIQPTLNSILIPKYMQ-------------------------------------
Linhu-GSK-III       NFTEHELRIQPVLNSMLIPKYMQSTA----------------------------------
                    **** ** *** * . *:*.: *

Drome-SGG           SAQPQSQGTAAAAGSGSGGATAGTGGASAGGPGSGNNSSSGGASGAPSAVAAGGANAAVA
Tal-SGG             ---------------------------ESGGSG------GGAVGSEAPTGAAGGS-----
Athro-GSK-III       ---------------------------TADNPG------AQSEASNVTAGASGNSSDNSA
Linhu-GSK-III       ---------------------AATTTGTEGNPGSGGGGGGQSDTTGATASASGNSNDNSV
                                                 ...*      . .     .: *:*.:

Drome-SGG           GGAGGGGGAGAATAAATATGAIGATNAGGANVTGSQSNSALNSSGSGGSGNGEAAGSGSG
Tal-SGG             -----------------------VTSANTATAAGAE------------------------
Athro-GSK-III       N---------------------ISTPTTTQNTDPSQSNMA--------------------
Linhu-GSK-III       TNP---------------NTTANTSTTNTQNIDPNSSNMA--------------------
                                            : :   .    .

Drome-SGG           SGSGSGGGNGGDNDAGDSGAIASGGGAAETEAAASG
Tal-SGG             ------------------------------------
Athro-GSK-III       ------------------------------------
Linhu-GSK-III       ------------------------------------
```

Figure S14. Putative *Talitrus saltator* SHAGGY (SGG) protein

Alignment of *Drosophila melanogaster* SGG (Drome-SGG; Accession no. AAN09084) with the *T. saltator* SGG (Tal-SGG) deduced from the Trinity *de novo* transcriptome assembly, together with the top two tblastn species homologue sequences *Athalia rosae* GSK-III

(Athro-GSK-III; Accession No. XM_012400594) and *Linepithema humile* GSK-III (Linhu-GSK-III; Accession No. XM_012372547). '*' indicates identical amino acid residues in the two proteins, '.' and ':' indicate similar amino acid residues between the two proteins. In this figure a SMART identified serine/threonine protein kinase catalytic domain is highlighted in yellow.
