# Supplementary material for: Identification and temporal expression of putative circadian clock transcripts in the amphipod crustacean Talitrus saltator
Source: PeerJ. 2016 Oct 5;4:e2555. doi: 10.7717/peerj.2555 (PMC5068443; doi:10.7717/peerj.2555)
Supplement: Figure S15 — Alignment of Drosophila melanogaster SLIMB (Drome-SLIMB; Accession No. AAF55853) with the T. saltator SLIMB (Tal-SLIMB) deduced from the Trinity de novo transcriptome assembly, together with the top two tblastn species homologue sequences Athalia rosae βTrCP (Athro-βTrCP; Accession No. XM_012405697) and Linepithema humile βTrCP (Linhu-βTrCP; Accession No. XM_012367510). ’*’ indicates identical amino acid residues in the two proteins, ’.’ and ’:’ indicate similar amino acid residues between the two proteins. In this figure SMART identified domains of one D domain of beta-TrCP, one F box domain and seven WD40 domains are highlighted in yellow, green and blue respectively. [file peerj-04-2555-s015.pdf]

```
Drome-SLIMB        ------MMKMETDKIMDETNSNAQAFTTTMLYDPVR------KKDS----SPTYQTEREL
Tal-SLIMB          MDTSSPLLDLDDSHDDSKTNIYSAMVHSTTPTATHTNNANNNRRKKESKGSGEYTSQRES
Athro-βTrCP        ---------------------------MTILYDPAR------KKEPPTGASAQYSQERET
Linhu-βTrCP        ------MINMETDKIIDDTNA-SLQYPMTILYDPSC------KKEPSTGVSTQYGQEREI
                                               *    .        ::.     *  *  :**

Drome-SLIMB        CFQYFTQWSESGQVDFVEHLLSRMCHYQHGQINAYLKPMLQRDFITLLPIKGLDHIAENI
Tal-SLIMB          CLKMFEHWSEQDQLEFMEHLLSRMCHYQHGHINAFLKPMLQRDFISLLPKKGLDHVAEKI
Athro-βTrCP        CMKLFERWSEPEQVQFVEQLLARMCHYQHGHINAYLKPMLQRDFISLLPKKGLDHVAESI
Linhu-βTrCP        CMKFFERWTEPEQVHFVEQLLARMCHYQHGHINAYLKPMLQRDFISLLPKKGLDHVAESI
                   *:: * :*:*  *:.*:*:**:********:***:**********:*** *****:**.*

Drome-SLIMB        LSYLDAESLKSSELVCKEWLRVISEGMLWKKLIERKVRTDSLWRGLAERRNWMQYLFKPR
Tal-SLIMB          LSYLDGKSLRDAELVCREWQRVIADGVLWKKLIERKVRTDPLWKGLSERRGWGQFLFKPR
Athro-βTrCP        LSYLDADSLCAAEMVCKGWYRVISEGMLWKKLIERKIRTDSLWRGLAERTGWIQYFFKPR
Linhu-βTrCP        LSYLDAKSLVSAELVCKEWHRVISEGMLWKKLIERKVRTDSVWRGLAERRGWIQYLFKPR
                   *****..**  :*:**: * ***::*:*********:***.:*:**:** .* *::****

Drome-SLIMB        PGQTQRPHSFHRELFPKIMNDIDSIENNWRTGRHMLRRINCRSENSKGVYCLQYDDGKIV
Tal-SLIMB          PGEQHPSHSYYRKMYPKIIQNIKTIEANWRMGRHNLQKINCRSETSKGVYCLQYDDNKIV
Athro-βTrCP        PGETHPNHIFYRTLFPKIITDIDNIENNWRMGRHNLQRINCRSENSKGVYCLQYDDQKIV
Linhu-βTrCP        PGESHPNHNFYRSLYPKIVKDIESIENNWRMGRFNLQRINCRSENSKGVYCLQYDDQKIV
                   **: :  * ::* ::***: :*..** *** **. *::******.*********** ***

Drome-SLIMB        SGLRDNTIKIWDRTDLQCVKTLMGHTGSVLCLQYDDKVIISGSSDSTVRVWDVNTGEMVN
Tal-SLIMB          SGLRDNTIKMWDRNTLQCYKVLTGHTGSVLCLQYDERVIISGSSDSTVRVWDANTGEMTN
Athro-βTrCP        SGLRDNTIKIWDRNTLQCIKVLTGHTGSVLCLQYDDKAIISGSSDSTVRVWDANTGEMLN
Linhu-βTrCP        SGLRDNTIKIWDRNTLQCIKVLTGHTGSVLCLQYDDKAIISGSSDSTVRVWDANTGEMVN
                   *********:***. *** *.* ************::.**************.***** *

Drome-SLIMB        TLIHHCEAVLHLRFNNGMMVTCSKDRSIAVWDMTSPSEITLRRVLVGHRAAVNVVDFDEK
Tal-SLIMB          TLIHHCEAVLHLRFTNGLLVTCSKDRSIAVWDMVSASEINLRRVLVGHRAAVNVVDFDEK
Athro-βTrCP        TLIHHCEAVLHLRFNNGMMVTCSKDRSIAVWDMTSQTEITLRRVLVGHRAAVNVVDFDEK
Linhu-βTrCP        TLIHHCEAVLHLRFNNGMMVTCSKDRSIAVWDMTSQTEIALRRVLVGHRAAVNVVDFDEK
                   **************.**::**************.* :** ********************

Drome-SLIMB        YIVSASGDRTIKVWSTSSCEFVRTLNGHKRGIACLQYRDRLVVSGSSDNSIRLWDIECGA
Tal-SLIMB          YIVSASGDRTIKVWGTSTCEFVRTLNGHKRGIACLQYRDRLVVSGSSDNTIRLWDIEYGA
Athro-βTrCP        YIVSASGDRTIKVWNTSTCEFVRTLIGHKRGIACLQYRDRLVVSGSSDNTIRLWDIECGA
Linhu-βTrCP        YIVSASGDRTIKVWNTSNCEFVRTLNGHKRGIACLQYRDRLVVSGSSDNTIRLWDIECGA
                   **************.**.******* ***********************:******* **

Drome-SLIMB        CLRVLEGHEELVRCIRFDTKRIVSGAYDGKIKVWDLVAALDPRAASNTLCLNTLVEHTGR
Tal-SLIMB          CLRILDGHEELVRCIRFDNKRIVSGAYDGKIKVWDLHAALDPRSPAGTLCLRTLVEHSGR
Athro-βTrCP        CLRVLEGHEELVRCIRFDNKRIVSGAYDGKIKVWDLVSALDPRAPASTLCLRTLVEHTGR
Linhu-βTrCP        CLRVLEGHEELVRCIRFDSKHIVSGAYDGKIKVWDLVAALDPRAVASTLCLRTLVEHTGR
                   ***:*:************.*:*************** :*****: :.****.*****:**

Drome-SLIMB        VFRLQFDEFQIVSSSHDDTILIWDFLNFTPNEN--KTGR---------------------
Tal-SLIMB          VFRLQFDEFQIVSSSHDDTILIWDFLNCSPPDTPPLQGPLASSPASDTGVSAAGGGGGTA
Athro-βTrCP        VFRLQFDEFQIVSSSHDDTILIWDFLNFSPPEGTPASGNASINTS---------------
Linhu-βTrCP        VFRLQFDEFQIVSSSHDDTILIWDFLNYNPSSGSVCSGRLPMDG----------------
                   *************************** .* .     *

Drome-SLIMB        ----------TPSPALMEH------------------------------
Tal-SLIMB          TAGAAQPVVQPPGPVLFRGAEGPAPLPCPTPPLPSNDSMDRSIDEGQD
Athro-βTrCP        ----VANQSSTRSPSPFE-------------------------------
Linhu-βTrCP        ----APIQSDTRSPSPFE-------------------------------
                             . .*  :.
```

Figure S15. Putative *Talitrus saltator* SUPERNUMERARY LIMBS (SLIMB) protein

Alignment of *Drosophila melanogaster* SLIMB (Drome-SLIMB; Accession No. AAF55853) with the *T. saltator* SLIMB (Tal-SLIMB) deduced from the Trinity *de novo* transcriptome assembly, together with the top two tblastn species homologue sequences *Athalia rosae*

βTrCP (Athro-βTrCP; Accession No. XM_012405697) and *Linepithema humile* βTrCP (Linhu-βTrCP; Accession No. XM_012367510). '*' indicates identical amino acid residues in the two proteins, '.' and ':' indicate similar amino acid residues between the two proteins. In this figure SMART identified domains of one D domain of beta-TrCP, one F box domain and seven WD40 domains are highlighted in yellow, green and blue respectively.
