# Supplementary material for: Identification and temporal expression of putative circadian clock transcripts in the amphipod crustacean Talitrus saltator
Source: PeerJ. 2016 Oct 5;4:e2555. doi: 10.7717/peerj.2555 (PMC5068443; doi:10.7717/peerj.2555)
Supplement: Figure S16 — lns:o=”urn:schemas-microsoft-com:office:office” xmlns:w=”urn:schemas-microsoft-com:office:word” xmlns:m=”http://schemas.microsoft.com/office/2004/12/omml” xmlns=”http://www.w3.org/TR/REC-html40”¿Alignment of Drosophila melanogaster VRI (Drome-VRI; Accession No. AAF52237) with the T. saltator VRI (Tal-VRI) deduced from the Trinity de novo transcriptome assembly, together with the top two tblastn species homologue sequences Clunio marinus VRI (Cluma-VRI; Accession No. JQ011276) and Acyrthosiphon pisum VRI (Acypi-VRI; Accession No. FM998650). ’*’ indicates identical amino acid residues in the two proteins, ’.’ and ’:’ indicate similar amino acid residues between the two proteins. In this figure a SMART identified basic region leucin zipper domain is highlighted in yellow. [file peerj-04-2555-s016.pdf]

```
Drome-VRI       MSIVCTLEQKVNFFKAAATTKNLLILKNNTDTNYINNYKQDNPSNNKFPRIQAQSNNSHL
Tal-VRI         -----------------------------MTAVMQN-----------------------V
Cluma-VRI       ------------------------------------------------------------
Acypi-VRI       -----------------------------MVADTANSYHHQQKTQQQQP-----------


Drome-VRI       QHQQQLQQKLAQLHHYSQQKLSGSDFPYGPRPPTGGKEEKLLLLAPPGKLYPEASVSTAM
Tal-VRI         LQREIMVAETVKSYPYP-------------------------------------ALLPLA
Cluma-VRI       -------VMVTELHHQE----SG-------APINGG------------------TIQRNI
Acypi-VRI       QQLNQHQHQQQQQHHHQ-------------------------------------QQQQKL
                           : :

Drome-VRI       PEVLSGTPTNSHNKANIAMMNNVRLSNISPTLSMNGSSNEASNLHPLSMYGGSISPQSND
Tal-VRI         QQGLNYSCTTSSCGPSLLPQALGQPYPAQASPIMERNHVGRARNAPSLGANVSSTPSG--
Cluma-VRI       DSGLS-KSCHYSNDSSVLML---RSDSMSPTMMS-----EMGDEH----YSKDSSPNS--
Acypi-VRI       HQQQQQQLHQIHLQRQQQQQ---QEQNSGGAMPQYCSDMEQSGGPGSMRYDDSVSPGP--
                 .  .          .       :      :          .        . . :*

Drome-VRI       SGMSDSLGKYVPGSGYGDGMMAQSPSQGGNGPQ---SALTAAQKELFSQRKQREFTPDNK
Tal-VRI         -----------------------LGASGMIGAN-----SALLKDSIFAQRKQREFIPDSK
Cluma-VRI       ----------------------PTPYDG-----------SVKRKDIFSQRKQREFIPDAK
Acypi-VRI       ----------------------ESPGPGEPYPPGFDLTAHLQHKEFFAQRKQREFIPDNK
                                           *              :..:*:******* ** *

Drome-VRI       KDESYWDRRRRNNEAAKRSREKRRYNDMVLEQRVIELTKENHVLKAQLDAIRDKFNISGE
Tal-VRI         KDESYWDRRRRNNEAAKRSREKRRFNDMILEQRVIELSKENHILRAQLTALENKFHVKGE
Cluma-VRI       KDDSYWDRRRRNNEAAKRSREKRRFNDMVLEQRVIELTKENHVLKAQLDAIKDKYNISGE
Acypi-VRI       KDESYWDRRRRNNEAAKRSREKRRFNDMILEQRVVELSKENHVLKAQLSAIKDKFGISGE
                **:*********************:***:*****:**:****:*:*** *:.:*: :.**

Drome-VRI       NLVSVEKILASLPTSEQVLSNTKRAKMSGSGGSSSGSSPSGSGSGEGSPQGGHNGYPVGP
Tal-VRI         GLVNEEQVLSSMPQADQILSLTRRSNLSLLSMTSP----------------------TS
Cluma-VRI       NLVSVDQIMATLPTSEQVLSLTKR------------------------------------
Acypi-VRI       AVVSVEQVMASLPTNEQVLSITKRPKLSTSSSSSS----------------------VG-
                 :*. :::::::*  :*:** *:*

Drome-VRI       PLSPLIYGPNGNARPEATVKSVHHIHHAGVAPPPTHLQQLVVPQSQTQHLYQPQPQQHQP
Tal-VRI         LLSPTSMPSTSPAP---------------------------------------------P
Cluma-VRI       ------------------------------------------------------------
Acypi-VRI       ------YSQNGSG----------------------------------------------P


Drome-VRI       HQQQQISQPPQQQQQQQEPSPSAGSSSPVISDPHNRPPSTTIANLQVQLQQALNRNVRPE
Tal-VRI         QQSSHSEDDHHFAVPQYSQHQHMEQSLPSPPQSNARSQSPDYYQRDVPLQAQSSQ-----
Cluma-VRI       ------------------------------------------------------------
Acypi-VRI       IPTSVIHQPVQSTTPKMNGSRTMAVHSPTAT-----------------------------


Drome-VRI       DLDSLRKVVAAGALYNAAAVVGAPPPPPSAGLYVPAPSAYKDHLEAAAAWSHNVEAAVSS
Tal-VRI         ---------------------------------------------AHSTYSSESHHLYES
Cluma-VRI       ------------------------------------------------------------
Acypi-VRI       ------------------------------------------------------------


Drome-VRI       SAVDAVSSSSVSGSAASVLNLSRRACSPSYEHMLSSTTSSTLSSASSSGAVSGDDEQEHE
Tal-VRI         TALNLSSRSSRSPNNMDCCYEQNRSLDYGGSSLPHKLRHKNQQHNTHSMSNNFNNPSCPM
Cluma-VRI       ------------------------------------------------------------
Acypi-VRI       ------------------------------------------------------------


Drome-VRI       PAHMAPLQLQRSSPQQGSDANNCLPLKLRHKSHLGDKDAAATALLSLQHIKQEPNCSRAS
Tal-VRI         GRPLSTSPLQDQMPPTSPHHYQQISSPMMVNGQHSGTRSSTSPSSPTSSHMLYPIKSEPI
Cluma-VRI       ------------------------------------------------------------
Acypi-VRI       ------------------------------------------------------------
```

```
Drome-VRI       PPAWNDGGDNSSDERDSGISIASAEWTAQLQRKLLAPKEANVVTSAERDQMLKSQLERLE
Tal-VRI         SREAGEESPGSSDDRDSGISLASSPPLSGAQSYPSSNRDSTEDMDCDSEQQLRVELQRLA
Cluma-VRI       ------------------------------------------------------------
Acypi-VRI       ------------------------------------------------------------

Drome-VRI       SEVASIKMILAE-------------
Tal-VRI         TEVRSLKSIISRNVDSNRQRDSPPRR
Cluma-VRI       --------------------------
Acypi-VRI       --------------------------
```

Figure S16. Putative *Talitrus saltator* VRILLE (VRI) protein

Alignment of *Drosophila melanogaster* VRI (Drome-VRI; Accession No. AAF52237) with the *T. saltator* VRI (Tal-VRI) deduced from the Trinity *de novo* transcriptome assembly, together with the top two tblastn species homologue sequences *Clunio marinus* VRI (Cluma-VRI; Accession No. JQ011276) and *Acyrthosiphon pisum* VRI (Acypi-VRI; Accession No. FM998650). '*' indicates identical amino acid residues in the two proteins, '.' and ':' indicate similar amino acid residues between the two proteins. In this figure a SMART identified basic region leucin zipper domain is highlighted in yellow.
