# Supplementary material for: Identification and temporal expression of putative circadian clock transcripts in the amphipod crustacean Talitrus saltator
Source: PeerJ. 2016 Oct 5;4:e2555. doi: 10.7717/peerj.2555 (PMC5068443; doi:10.7717/peerj.2555)
Supplement: Figure S17 — Alignment of Drosophila melanogaster EBONY (Drome-EBONY; Accession No. AAF55870) with the T. saltator EBONY (Tal-EBONY) deduced from the Trinity de novo transcriptome assembly, together with the top two tblastn species homologue sequences Tribolium castaneum EBONY (Trica-EBONY; Accession No. XM_008199683) and Periplaneta americana EBONY (Peram-EBONY; Accession No. AJ865468). ’*’ indicates identical amino acid residues in the two proteins, ’.’ and ’:’ indicate similar amino acid residues between the two proteins. In this figure SMART identified domains consisting of one AMP binding domain, one AMP binding C domain and one PP binding domain are highlighted in yellow, green and blue respectively. [file peerj-04-2555-s017.pdf]

```
Drome-EBONY      MGSLPQLSIVKGLQQDFVPRALHRIFEEQQLR-HADKVALIYQPSTTGQGMAPSQSSYRQ
Tal-EBONY        -----MVSTLRGPTQCPPPTAHSVVEGFQWAALAHPHKAALVDP------QRGTRMTYAQ
Trica-EBONY      MGSLPQLSILKGPTRPLVVSQLGRIFEKVAAGPAASNIALIFE--ENGQ---SRQLSYSQ
Peram-EBONY      MGSIPTLSILRGDPRPLDSEASLHRLLERAAGASAQQTALLFH---------NQSTSFGA
                       :* ::*  :                     : * : .             ::

Drome-EBONY      MNERANRAARLLVAETHGRFLQPNSDGDFIVAVCMQPSEGLVTTLLAIWKAGGAYLPIDP
Tal-EBONY        LRDEVNAIARVLEAHLR-DAPNHNRDGDVLVALCMSPSPRLVATILAVMQVGAAYVALDP
Trica-EBONY      VDQITNKFARVISETIKTKNLKPNSDGDHIIAVNMHPSDHLILVLLSIWKSGCAYLPLDH
Peram-EBONY      LDAAASRLARALVRRARNVGARPNSDGDFLVAVCMEPSERLVVALLAVWKAGAAYLPLDP
                 :   ..  ** :    :    . * *** ::*: * **  *: .:*:: : * **:.:*

Drome-EBONY      SFPANRIHHILLEAKP-TLVIRDDDIDAGRFQGTPTLSTTELYAKSLQLAGSNLLSEEML
Tal-EBONY        NFPPSRVDHILQDCAPQLLVVEDGGIMPQSAPYTTTLTTSTIERQMRSQEGTTNSPSVRV
Trica-EBONY      SFPASRIDHIIQESKP-VIVIYDE--DSKNFP--AKLSYEELLTASDGRSDKRLEDKERV
Peram-EBONY      AFPQARVAHILGEARP-VLVLAEKE-DPELFS--DAALYEDLRREAADLSSEPLPDAETL
                  **  *: **: :. *  :*: :    .             :        .        :

Drome-EBONY      RGGNDHTAIVLYTSGSTGVPKGVRLPHESILNRLQWQWATFPYTANEAVSVFKTALTFVD
Tal-EBONY        CG-GEDVAVVLYTSGSTGVPKGVRLPHRALLNRLQWQWHTFPYEENE-VCVFKTALTFVD
Trica-EBONY      YQ-KDDLAIVLYTSGSTGIPKGVRIPHKIILNRLQWQFKTFPFSETEKVCVFKTALTFVD
Peram-EBONY      PGCAPTLALVLYTSGSTGVPKGVRLPHAVVLNRLRWQWRTFPYGPQERVCVFKTALTFVD
                        *:*********:*****:**  :****:**: ***:   * *.**********

Drome-EBONY      SIAELWGPLMCGLAILVVPKAVTKDPQRLVALLERYKIRRLVLVPTLLRSLLMYLKMEGG
Tal-EBONY        SLSEVLGPLLTGRTLVVVAKSTVARVESLVEVLTQHRVRRLVLVPTLLRALLLHCSRLP-
Trica-EBONY      SVCEIWGPLISGISILIVPKSVTLDPEKLIQKLDDYKIERLVLVPSLLRSILMCLELK--
Peram-EBONY      SVGELWGPLLQERSVLVVPREVTKDPERLLRVLEDHQIERLVLVPSLLRSLLLALGLES-
                 *: *: ***:   ::::*.: ..   : *:  *  :::.******:***::*:

Drome-EBONY      GAAQKLLYNLQIWVCSGEPLSVSLASSFFDYFDEGVHRLYNFYGSTEVLGDVTYFACESK
Tal-EBONY        --SHARLPHLKLWVCSGEQFPLDLLENFFQVFTQ-EQGICNFYGSTEVMGDVTFVHFKSV
Trica-EBONY      -KNRTLLKNLKLWVCSGETLTTSLAEEFFRYFPENEYKLCNFYGSTEIMGDVTFYIISGM
Peram-EBONY      -RNKGMLSRLKTWVCSGEPLSAQLAREFFTYFESGDHILCNFYGSTEVMGDVTYHVVRSA
                    :  * .*: ****** :. .*  .**  * .    : *******::****:    .

Drome-EBONY      KQLSL---YDNVPIGIPLSNTVVYLLD--ADYRP---------------------------
Tal-EBONY        EDVRLKVVSSSVPIGEVVSNNCIVLGSDVEELRQRCAAAGGASAAPHSASSAGSANSVTN
Trica-EBONY      QQLKN---LLTVPIGAPVDNTIVYLLD--PELRP---------------------------
Peram-EBONY      AELKD---STKVPIGRPLDNTAIYLLD--DNFRP---------------------------
                  ::       .****  :.*. : * .   : *

Drome-EBONY      ---------------------------------------VKNG-----------------
Tal-EBONY        GNGVTNGNGVANGNGVANGNGVTNGTIVTHRNGVANGNGVQNGNSVANGNGITNGSSITN
Trica-EBONY      ---------------------------------------VKTG-----------------
Peram-EBONY      ---------------------------------------VVSG-----------------
                                                        * .*

Drome-EBONY      ---------------------------------------EIGEIFASGLNLAAGYVNGRD
Tal-EBONY        GNGTANGNAVTDSTTSGKDCSSPPGVEGHGAPQGGRGGHQKGEMLVYGANLALGYVGVGG
Trica-EBONY      ---------------------------------------DIGELFVSGLNLASGYVNNRD
Peram-EBONY      ---------------------------------------EAGELYVSGLNLAQGYVKGRD
                                                        : **: . * *** ***   .

Drome-EBONY      PERFLENPLAVEKKYARLYRTGDYGSLK-NGSIMYEGRTDSQVKIRGHRVDLSEVEKNVA
Tal-EBONY        ADKFFH--LLIDGKTELVYRTGDFATLHPSGTLLYDGRTDGQIKIRGHRVDCGEVQRAVQ
Trica-EBONY      KEKFLENQLAIDPIFSKLYRTGDFARLQ-NDVLLYEGRTDSQVKIRGHRVDLSEVEKAVS
Peram-EBONY      PERFLANPLTVDPEHSRLYRTGDFARFV-KGTLVYEGRTDSQVKIRGHRVDLAEVERAVA
                  ::*:   * ::     :*****:. :  .. ::*:****.*:******** .**:: *

Drome-EBONY      ELPLVDKAIVLCYHAGQVDQAILAFVKLRDDAPMVTETQMEARLKDKLADYMTPQVVILE
Tal-EBONY        ATEGVTSCAVLCHAPGTVQQALVAFYCS-ETVEDDYEERLEASLASRLLTYMRPKLVRVP
Trica-EBONY      GVEGVEKAVVLCYQPGEMNQALLAFVKS---SALMNENQIENILRSKLTSYMVPQVILVE
Peram-EBONY      GLPGVDKCVVLCYKPGELEQALLAYVTTCKDSSMSSQ-QLEAVLHKNLAAYMLPQVFVVD
                     * .. ***: .* ::**::*:           : ::*  * ..*  ** *::. :
```

```
Drome-EBONY      HVPLLVNGKVDRQALLKTYETANNNEGDSSIVLDFDYSQVPEDLKLTARDLFETVGGVIG
Tal-EBONY        HMPLLVNGKVDRQKLLGDYSAALHHN-----EGSVDVSGAPEGLQREAVALLTVVGRVLG
Trica-EBONY      SIPLLVNGKIDRQALLKSYENTNNND-DSSVEIEIDYSGVKPAQMAAAKVLFDTVASVLN
Peram-EBONY      SIPLLVNGKTDRQALLRRYESYSTSRNSESEGPELDYTGVPANRLDAARVLFETVAEVLG
                  :******* *** **  *.             ..* : .       *  *: .*. *:.

Drome-EBONY      RSTETTLPPHSNFYELGGNSLNSIFTVTLLREKGYNIGISEFIAAKNLGEIIEKMAANHD
Tal-EBONY        GGSKKKLSLSDNFFKIGGDSLNSVLVVTALGDLGYSLGLTDFLDCDDLEAVVRKMGGLKG
Trica-EBONY      RSARSAISLDSNFYEIGGNSLNSIYTITRLNEEGYQISIGDFLSAIDLGEVLERMTSSND
Peram-EBONY      GGTRSKVGLQANFYELGGNSLNSVFTVTKLRQLGYTIGITDFISSVNLQQVLERMQSNDD
                  .:.. :    **:::**:****: .:* * : ** :.: :*: . :*  ::.:* . ..

Drome-EBONY      AVQLEEESLNACPHLKMEAVPLRLEHRQEVIDIIVASFYNKADLEQWLKPGVLRTDYSDI
Tal-EBONY        GSEGTVP---PSCSSNYTFHPLQEEMEQEVIELLAVSFSSKSDME--VATGAKKEDFYPL
Trica-EBONY      ---------IHCSPPTYTSELLKNEHKSAVLDIITTSFYQKADLEQWLMPDIFESDYKEL
Peram-EBONY      TTATVS---EKRESKRYSAEMLQDQHKQSVNHMITESFYEKADLEQWLKPDIHREDYKDL
                                      *: : .. * .::. ** .*:*:*  : ..  . *:  :

Drome-EBONY      LNDIWNVLVERDLSFVVYDTNTDRIIGTALNFDARNEPEVDIKSKLLIVFEFLEFCEGPI
Tal-EBONY        LHGIYGDVLLQRLSFVVQDA-GGRIVACSLNFDLQAEPPVDVDYPLTWVFDFLDQCESKV
Trica-EBONY      MDALWEPLVEKALSFVAKAE-SGKIIGVGLNFDARDEPDVQITSKLTVIFEFLETVEGPV
Peram-EBONY      TDKLWEPLVQKNLSFIVKDT-QGVPVGVALNFDAHDEPAVEITSKLLIVFDFLEFLEGPI
                  . ::  :: : ***:.     .  :. .**** : ** *::   *  :*:**:  *. :

Drome-EBONY      RDNYLPKGLNQILHSFMMGTAEKLNPRENIACMHFMEHEVLRVAREKQFAGIFTTNTSPL
Tal-EBONY        R-NKLPQNVGEVVHSFMMSTLVDLSPKQNIELVKLMEEHNIRLARERQYKAVFTTNTSAA
Trica-EBONY      RDKCLPGGKGKILHSFMMGTHSSLSPKENVAVMQFMEDEVLKLATERHFEGIFTTNTSPL
Peram-EBONY      RDNKLPQGKGQVLHSFMMATHPSLNYQQNVEVIQFMEAEVLTIAHSRGFAGIFTTNTNPL
                 * : ** . .:::*****.*  .*. ::*:  :::** . : :* .: : .:*****..

Drome-EBONY      TQQL-ADVYHYKTLLNFQVNEYVHSDGSRPFGDAPDEQRAIVHWKEVGK------
Tal-EBONY        TRYVCDDLLGYTVLAETPFCSYTAADGQQPFASIPPDHVAACTVFYV--------
Trica-EBONY      TQQLGTDVYHYQTLLDYQVNRYVASDNTRPFGMAPDSQRAIVQWKPIKSDIVVDV
Peram-EBONY      TQQLGTDIYDYKVFNDYQVNLYEAPDGSKPFSEAPDSQRAVCSWRPI--------
                 *: :  *:  * .: :  .  *  .*. :**.  *  .: *      :
```

Figure S17. Putative *Talitrus saltator* EBONY protein

Alignment of *Drosophila melanogaster* EBONY (Drome-EBONY; Accession No. AAF55870) with the *T. saltator* EBONY (Tal-EBONY) deduced from the Trinity *de novo* transcriptome assembly, together with the top two tblastn species homologue sequences *Tribolium castaneum* EBONY (Trica-EBONY; Accession No. XM_008199683) and *Periplaneta americana* EBONY (Peram-EBONY; Accession No. AJ865468). '*' indicates identical amino acid residues in the two proteins, '.' and ':' indicate similar amino acid residues between the two proteins. In this figure SMART identified domains consisting of one AMP binding domain, one AMP binding C domain and one PP binding domain are highlighted in yellow, green and blue respectively.
