# Supplementary material for: Identification and temporal expression of putative circadian clock transcripts in the amphipod crustacean Talitrus saltator
Source: PeerJ. 2016 Oct 5;4:e2555. doi: 10.7717/peerj.2555 (PMC5068443; doi:10.7717/peerj.2555)
Supplement: Figure S18 — Alignment of Eurydice pulchra PDH (Ep-PDH; Accession no. GQ380440) with the T. saltator PDH-I and PDH-II (Tal-PDH-I and Tal-PDH-II) deduced from the Trinity de novo transcriptome assembly, together with the top two Tal-PDH-I tblastn species homologue sequences Marsupenaeus japonicus PDH-II (Marja-PDH-II; Accession No. AB073368), Daphnia magna PDH (Dapma-PDH; Accession No. HQ843173) and the Euphausia crystallorophias sequences PDH-La, PDH-LbI, and PDH-LbII (Eupcr-PDH-La, Eupcr-PDH-LbI, and Eupcr-PDH-LbII respectively). ’*’ indicates identical amino acid residues in the two peptides, ’.’ and ’:’ indicate similar amino acid residues. In this figure the SMART identified PDH domain is highlighted in yellow. The region of Tal-PDH-II that corresponds to the PDH regions of Drome-PDF and Tal-PDH-I is highlighted in blue, though no PDH domain per se was identified in this peptide using SMART. [file peerj-04-2555-s018.pdf]

```
Ep-PDH              --MRFIILGVLFIAVASMILSNGV------------------------------------
Tal-PDH-I           -MLSTTVAVWSAVVMLGCVAGGQD------------------------------------
Marja-PDH-II        --------MCRAAVLLFLMLAVAA------------------------------------
Dapma-PDH           MHQLSAKLSHFSIALLVLFVSLTMDVESAPAATSSINRPEAQLSIQEMEKFLEGLTRYLH
Eupcr-PDH-Lα        --------MRASVVFLGLFLAFIA------------------------------------
Eupcr-PDH-LβI       --------MRGLVMICFMVLVVGT------------------------------------
Eupcr-PDH-LβII      ------------------------------------------------------------
Tal-PDH-II          ----MASFKHQLLLLLGCMLCLAL------------------------------------

Ep-PDH              --------MAQ-----------SRDFSISEREIVASLAKQLLRVARMGYVPEGDLPR---
Tal-PDH-I           --------LGDLG---------SAPLTVPQRLAISDWAQSIARLADGGLDGPSGLSRRLE
Marja-PDH-II        --------VMVTEA--------QREPTASKCQAATELAIQILQAVKGAHTGVAAGPH---
Dapma-PDH           RQRLEQQRMHQPQAKSLMSEEVTYDPDAIDRAILGEMAAPLDAERSSSVELANNSMSHAR
Eupcr-PDH-Lα        --------INDVV---------AQSLTEQDSIVVASLAAQILDVVAGSENDGDSGVQAV-
Eupcr-PDH-LβI       --------IAI-----------VHAQEDQERQAVGNLALDILRVVGRAPSAMQ-------
Eupcr-PDH-LβII      -------------------------QEDQERQVVGELALGILRIVGQESSGPQ-------
Tal-PDH-II          --------VA------------AQPRRHPDLYSDDDVAAQLDGEGSYGAVPDDLAAWLI-
                                                 .     . *  :

Ep-PDH              -----------------------------KRNAELINSLLGVP------RVMSDAGRR
Tal-PDH-I           LLQQQVELGNLGGWRGSDRLSYGSTRPHDKRNSELINSLLGLP------KFLRE----
Marja-PDH-II        -----------------------------KRNSELINSLLGLP------KFMIDAGRR
Dapma-PDH           PPTNNKWPWSLSNFERIEDQNVKQRQPYGKRNSELINSLLGLP------RFMKVVG--
Eupcr-PDH-Lα        -----------------------------KRNSGTINSMLGLPRTYNLRRMMMHAGRK
Eupcr-PDH-LβI       -----------------------------KRNSELINSMLGLPQTLRAQKLMANMGR-
Eupcr-PDH-LβII      -----------------------------KRNAETINTMLGLPQTLRAQKLMAKLGR-
Tal-PDH-II          ----------------------RSRMP--KRNSELLNTLLGSKNL----VALRAAGRR
                                                 ***:  :*::**          :
```

Figure S18. Putative *Talitrus saltator* PIGMENT DISPERSING HORMONE (PDH) peptides

Alignment of *Eurydice pulchra* PDH (Ep-PDH; Accession no. GQ380440) with the *T. saltator* PDH-I and PDH-II (Tal-PDH-I and Tal-PDH-II) deduced from the Trinity *de novo* transcriptome assembly, together with the top two Tal-PDH-I tblastn species homologue sequences *Marsupenaeus japonicus* PDH-II (Marja-PDH-II; Accession No. AB073368), *Daphnia magna* PDH (Dapma-PDH; Accession No. HQ843173) and the *Euphausia crystallorophias* sequences PDH-La, PDH-LbI, and PDH-LbII (Eupcr-PDH-La, Eupcr-PDH-LbI, and Eupcr-PDH-LbII respectively). '*' indicates identical amino acid residues in the two peptides, '.' and ':' indicate similar amino acid residues. In this figure the SMART identified PDH domain is highlighted in yellow. The region of Tal-PDH-II that corresponds to the PDH regions of Drome-PDF and Tal-PDH-I is highlighted in blue, though no PDH domain *per se* was identified in this peptide using SMART.
