# Supplementary material for: Identification and temporal expression of putative circadian clock transcripts in the amphipod crustacean Talitrus saltator
Source: PeerJ. 2016 Oct 5;4:e2555. doi: 10.7717/peerj.2555 (PMC5068443; doi:10.7717/peerj.2555)
Supplement: Figure S19 — Alignment of Drosophila melanogaster RORA (Drome-RORA; Accession No. NP_788301) with the T. saltator RORA (Tal-RORA) deduced from the Trinity de novo transcriptome assembly, together with the top two tblastn species homologue sequences Drosophila grimshawi RORA (Drogr-RORA; Accession No. XM_001987136) and Drosophila ananassae RORA (Droan-RORA; Accession No. XM_001959222). ’*’ indicates identical amino acid residues in the two proteins, ’.’ and ’:’ indicate similar amino acid residues between the two proteins. In this figure SMART identified domains consisting of one C4 zinc finger domain and one HOLI ligand binding domain are highlighted in yellow and green respectively. [file peerj-04-2555-s019.pdf]

```
Drome-RORA      MASLLGSAPPAQILANQPIIVKIEPTQSFHIVDEGDTRVLSLPLSDADKLGASWIDLKDI
Tal-RORA        -------MDDSTLDLDTSGLLRVGGLLQSSLQDGSGSMGDSSSNRDEDMMGITHISPANS
Drogr-RORA      ------MYTQRMFDMWSSVTSKLEA--------------------HANNLG---------
Droan-RORA      ------MYTQRMFDMWSSVTSKLEA--------------------HANNLG---------
                            :    .   .:                        : :*

Drome-RORA      AGLQAGGGATLLDVCFEQANEDGTIIATVQPDLENELEAELKAEGEPEDETEPEPPAPKR
Tal-RORA        SQFKQQQESQLSTTTPNILGFPGGNPSSNSGSTLLNISSIPNLTSPPLSCASSNQNQSER
Drogr-RORA      ------------------------------------------------------------
Droan-RORA      ------------------------------------------------------------

Drome-RORA      LATTRPAQSRPQQQQQQQQQQVKFLSDPPALARSSSFS--SLSSFSSISNISSVCKNMAS
Tal-RORA        ----SPHLQQPHSSNNNRDCPAAFETNRSFLESCEDFTLDEISSDRSASTMSSLQNIPSI
Drogr-RORA      ----QSNVQSPAAQNN--------------------------------------------
Droan-RORA      ----QSNVQSPAGQNN--------------------------------------------
                     .  . *  .::

Drome-RORA      NTSQEGSLKRTKERTPPPMPPLTTHKPAATTTATSATSAAAATSAATASATATSARENSR
Tal-RORA        SGSKMGLLERKADRD-------LLNEILAGINEDRDRERDRDRDMADTTTPTSSTPTPTP
Drogr-RORA      ------------------------------------------------------------
Droan-RORA      ------------------------------------------------------------

Drome-RORA      EHSSSSSGSNGAMTAQIEIIPCKVCGDKSSGVHYGVITCEGCKGFFRRSQSSVVNYQCPR
Tal-RORA        RIQSVADKKCNSIKAQIEIIPCKVCGDKSSGVHYGVITCEGCKGFFRRSQSSVVNYQCPR
Drogr-RORA      --------SSGSIKAQIEIIPCKVCGDKSSGVHYGVITCEGCKGFFRRSQSSVVNYQCPR
Droan-RORA      --------SSGSIKAQIEIIPCKVCGDKSSGVHYGVITCEGCKGFFRRSQSSVVNYQCPR
                        . .::.**********************************************

Drome-RORA      NKQCVVDRVNRNRCQYCRLQKCLKLGMSRDAVKFGRMSKKQREKVEDEVRFHRAQMRAQS
Tal-RORA        QKNCVVDRVNRNRCQYCRLQKCLQLGMSRDAVKFGRMSKKQREKVEDEVRYHKSQMRAAS
Drogr-RORA      NKQCVVDRVNRNRCQYCRLQKCLKLGMSRDAVKFGRMSKKQREKVEDEVRFHRAQMRAQS
Droan-RORA      NKQCVVDRVNRNRCQYCRLQKCLKLGMSRDAVKFGRMSKKQREKVEDEVRFHRAQMRAQS
                :*:********************:**************************:*.:**** *

Drome-RORA      --DAAPDSSVYDTQTPSSSDQLHHNNYN--SGGYSNNEVGYGSPYGYSASVTPQQTMQYD
Tal-RORA        GPETSPDSSVYEPTSP--SQDMFSAQY-----GFGSNELNFNNHYSFSPRPGGASGSSLD
Drogr-RORA      --DAAPDSSVYDTQTPSSSDQLQHTNYNSYSGGYSSNEVGYGSPYGYSASVTPQQTMQYD
Droan-RORA      --DAAPDSSVYDTQTPSSSDQLHHNNYNSYSGGYSNNEVGYGSPYGYSASVTPQQTMQYD
                  :::******:. :*  *:::   :*     *:..**:.:.. *.:*.     .  . *

Drome-RORA      ISADYVDSTTYEPRSTIIDPEFISHADGDINDVLIKTLAEAHANTNTKLEAVHDMFRKQP
Tal-RORA        ISS--ADSTTYDPQSR------------GCLDIQAQTSLDS-GDTDSHSSPLLSACRNER
Drogr-RORA      ISADYVDSTTYEPRSTMIDPEFISHADGDINDVLIKTLAEAHANTNTKLESVNDMFRKPQ
Droan-RORA      ISADYVDSTTYEPRSTIIDPEFISHADGDINDVLIKTLAEAHNNTNTKLEAVHDMFRKQP
                **:  .*****:*.*             .  *:  :*  ::  :*::: ..: .  *:

Drome-RORA      DVSRILYYKNLGQEELWLDCAEKLTQMIQNIIEFAKLIPGFMRLSQDDQILLLKTG--SF
Tal-RORA        Q---------------W-----KNSEAVPASWDYRKSVQGFRNLPLD-----IRSGGCSS
Drogr-RORA      DLSRILYYKNLGQEELWLDCAEKLTQMIQNIIEFAKLIPGFMRLSQDDQILLLKTG--SF
Droan-RORA      DVSRILYYKNLGPEELWLDCAEKLTQMIQNIIEFAKLIPGFMRLSQDDQILLLKTG--SF
                :               *     * :: :    :: * : ** .*. *     :.:*  *

Drome-RORA      ELAIVRMSRLLDLSQNAVLYG-DVMLPQEAFYTSDSEEMRLVSRIFQTAKSIAELKLTET
Tal-RORA        NMQGAMKPDLMEGGGGSLSHDMDILIKQEV----DGLDMNNLQSSHISGDSVETDMASPD
Drogr-RORA      ELAIVRMSRLLDLSQNAVLYG-DVMLPQEAFYTSDSDEMRLVSRIFQTAKSIAELKLTET
Droan-RORA      ELAIVRMSRLLDLSQNAVLYG-DVMLPQEAFYTSDSEEMRLVSRIFQTAKSIAELKLTET
                ::  .  . *:: . .:: :. *::: **.    *. :*. :.  . :..*:     :

Drome-RORA      ELALYQSLVLLWPERNGVRGNTEIQRLFNLSMNAIRQELETNHAPLKGDVTVLDTLLNNI
Tal-RORA        HMTSVDSTTFLPCPSVSPQPNSSQA---GLHLHHDRPHMGPFGAADHGDFTDLHHHQQQQ
Drogr-RORA      ELALYQSLVLLWPERNGVRGNTEIQRLFNLSMNAIRQELETNHAPLKGDVTVLDTLLNNI
Droan-RORA      ELALYQSLVLLWPERNGVRGNTEIQRLFNLSMNAIRQELETNHAPLKGDVTVLDTLLNNI
                 ::  :* .:*     .  . *:.     .* ::  *  :  .  *. :**.* *    ::
```

```
Drome-RORA      PNFRDISILHMESLSKFKLQHPNVVFPALYKELFSIDSQQDLT
Tal-RORA        QQQQ------------------------------EQQQQQQ
Drogr-RORA      PNFRDISILHMEALSKFKQQHPTVVFPALYKELFSIDSQQDLT
Droan-RORA      PNFR---------------------------------------
                 : .
```

Additional file 24. Putative *Talitrus saltator* RORA protein

Alignment of *Drosophila melanogaster* RORA (Drome-RORA; Accession No. NP_788301) with the *T. saltator* RORA (Tal-RORA) deduced from the Trinity *de novo* transcriptome assembly, together with the top two tblastn species homologue sequences *Drosophila grimshawi* RORA (Drogr-RORA; Accession No. XM_001987136) and *Drosophila ananassae* RORA (Droan-RORA; Accession No. XM_001959222). '*' indicates identical amino acid residues in the two proteins, '.' and ':' indicate similar amino acid residues between the two proteins. In this figure SMART identified domains consisting of one C4 zinc finger domain and one HOLI ligand binding domain are highlighted in yellow and green respectively.
