# Supplementary material for: Identification and temporal expression of putative circadian clock transcripts in the amphipod crustacean Talitrus saltator
Source: PeerJ. 2016 Oct 5;4:e2555. doi: 10.7717/peerj.2555 (PMC5068443; doi:10.7717/peerj.2555)
Supplement: Figure S20 — Alignment of Drosophila melanogaster E75 (Drome-E75; Accession No. NP_730321) with the T. saltator (Tal-REVERB) deduced from the Trinity de novo transcriptome assembly, together with the top two tblastn species homologue sequences Blattella germanica E75 (Blage-E75; Accession No. AM710419) and Apis dorsata E75 (Apido-E75; Accession No. XM_006618394). ’*’ indicates identical amino acid residues in the two proteins, ’.’ and ’:’ indicate similar amino acid residues between the two proteins. In this figure SMART identified domains consisting of one C4 zinc finger domain and one HOLI ligand binding domain are highlighted in yellow and green respectively. [file peerj-04-2555-s020.pdf]

```
Drome-E75       MEAVQAAAAATSSGGSSGSVPGSGSGSASKLIKTEPIDFEMLHLEENERQQDIEREPSSS
Tal-REVERB      -MGSTSSDAGVPLISSTQDGDNRTIGHSTNKLRQGREFVQNIEFPYTLKTTDIVRATSSD
Blage-E75       ----------------------------------------------MTDTKDFRIPTSVI
Apido-E75       ------------------------------------------------MSADVARDD---
                                                                   *.

Drome-E75       NSNSNSNSLTPQRYTHVQVQTVPPRQPTGLTTPGGTQKVILTPRVEYVQQRATSSTGGGM
Tal-REVERB      DRKFDFAYSLADGSQAGIIASSTMHSKNG-------------------------------
Blage-E75       A-----------------------------------------------------------
Apido-E75       ------------------------------------------------------------

Drome-E75       KHVYSQQQGTAASRSAPPETTALLTTTSGTPQIIITRTLPSNQHLSRRHSASPSALHHYQ
Tal-REVERB      KSIYPYPHEEKIKIKQEPSEYADLQSQQATSVLHMKGEFDSKVGHVKQEKKEDFARWWKN
Blage-E75       ------------------------------------------------------------
Apido-E75       ------------------------------------------------------------

Drome-E75       QQQPQRQQSPPPLHHQQQQQQQHVRVIRDGRLYDEATVVVAARRHSVSPPPLHHHSRSAP
Tal-REVERB      DDDEKPLKSEPQMKGQRDEDMM--------------------------PPPKG-------
Blage-E75       ------------------------------------------------PPPL--------
Apido-E75       ------------------------------------------------PSEM--------
                                                                *.

Drome-E75       VSPVIARRGGAAAYMDQQYQQRQTPPLAPPPPPPPPPPPPPPPPQQQQQQYISTGVPPPTA
Tal-REVERB      ---------------------------PPPSSLGNIHRFSKPGKHHKIQDFRKNFPGAQY
Blage-E75       ------------------------------------------------------------
Apido-E75       ------------------------------------------------------------

Drome-E75       AARKFVVSTSTRHVNVIASNHFQQQQQQHQAQQHQQQHQQHQQHQQHVIASVSSSSSSSA
Tal-REVERB      LPSYPNITDVSRFPSVVSSEMWHAHVSSYRMSLDQRAMYETEGAP---------------
Blage-E75       ------------------------------------------------------------
Apido-E75       ------------------------------------------------------------

Drome-E75       IGSGGSSSSHIFRTPVVSSSSSSNMHHQQQQQQQQSSLGNSVMRPPPP-----------P
Tal-REVERB      -----------------SSSSVNELAKSSSDAQEPSSSGHLAPKSTKPEANIFTVKKNQP
Blage-E75       ---------------------------VSSSTCXPPGTASSTMRPPPP-----------P
Apido-E75       -------------------------------------VDHTTTRNNGV-----------P
                                                         . .               *

Drome-E75       P-PPKVKHASSSSSGNSSSSNTNNSSSSSNGEEPSSSIPDLEFDGTTVLCRVCGDKASGF
Tal-REVERB      P-PANVENFYQTH--GTSGGRKAAHTRRRDENIVSSSISDLEFDGTTLLCRVCGDRSSGF
Blage-E75       P-PPKARS-------NGVAGGCDVKSERNHHEEPSSSIPDLEFDGTTVLCRVCGDKASGF
Apido-E75       IYPIEVCE-------ATIAVNETQNPERRH-------LRVKEFDGTTVLCRVCGDKASGF
                  * :.            .      .           :   ******:*******.:***

Drome-E75       HYGVHSCEGCKGFFRRSIQQKIQYRPCTKNQQCSILRINRNRCQYCRLKKCIAVGMSRDA
Tal-REVERB      HYGVHSCEGCKGFFRRSIQQKIQYRPCSKNQQCLVQKTNRNRCQYCRLKKCIEVGMSKDA
Blage-E75       HYGVHSCEGCKGFFRRSIQQKIQYRPCTKNQQCSILRINRNRCQYCRLKKCIAVGMSRDA
Apido-E75       HYGVHSCEGCKGFFRRSIQQKIQYRPCTKNQQCSILRINRNRCQYCRLKKCIAVGMSRDA
                ***************************:***** : . ************** ****.**

Drome-E75       VRFGRVPKREKARILAAMQQSTQNRGQQRALATELDDQPRLLAAVLRAHLETCEFTKEKV
Tal-REVERB      VRFGRVPKNEKERIRQAMEKASSNIPQQDY------NDDFLIQSITRSHLQSSAVANENM
Blage-E75       VRFGRVPKREKARILAAMQQSSNSRSQEKAVAAELEDEQRLLSTVVRAHLDTCDFTREKV
Apido-E75       VRFGRVPKREKARILAAMQQSSHSRSQEKAVAAELEDEQRLLATVVQAHLDTCDFTRDKV
                ********.** **  **:::: .  *:        ::  *: :: .:**::. .:.:::

Drome-E75       SAMRQRARDCPSYS-MPTLLACPLNPAPE-LQSE----QEFSQRFAHVIRGVIDFAGMIP
Tal-REVERB      REIIRTSEEHPQVANIDPISDCPLNP----YVGT----EDFQQKFSPIVRDVVAFAKNIP
Blage-E75       EPMLARARDQPSYTACPPTLACPLNPNPQPLTGQQELLQDFSKRFSPAIRGVVEFAKRIP
Apido-E75       APILVRARETPNYTACPPTLACPLNPNPQPLTGQQELLQDFSKRFSPAIRGVVEFAKRIP
                  :   : : *. :   .   *****      .     ::*.:.*:  :*.*: **  **
```

```
Drome-E75       GFQLLTQDDKFTLLKAGLFDALFVRLICMFDSSINSIICLNGQVMRRDAIQNGANARFLV
Tal-REVERB      GFSDIKHDDKITLLRACVFEVLLIRFARLIDAKNRRMITISGYIINASIYANIKTSNDFF
Blage-E75       GFALLPQDDQVTLLKAGVFEVLLVRLACMFDAQTNSMICLNGQVLKREAIHNSSNARFLM
Apido-E75       GFSLLAQDDQVTLLKAGVFEVLLVRLACMFDAQTNSMICLNGQVLKRESIHNSSNARFLM
                **  : :**:.***.* :*:.*::*:  ::*:. . :* :.* ::. .   *  .:. :.

Drome-E75       DSTFNFAERMNSMNLTDAEIGLFCAIVLITPDRPGLRNLELIEKMYSRLKGCLQYIVAQN
Tal-REVERB      NTLFTFVDRINTLKLSDEEMALFSAAIVINPKRQGLHELSKVSSLHKRIVHCLQVIMQRE
Blage-E75       DSMFDFAERLNSLRLSDAEVGLFCSVVVIAPDRPGLRNTELIERMQGKLKAALQMVVSQN
Apido-E75       DSMFDFAERVNSLRLSDAELGLFCSVVVIAADRPGLRNTELVERMHNKLRNALQTVLAQN
                :: * *.:*:*::.*:* *:.**.: ::* ..* **.: . :. :  .:  .** :: .:

Drome-E75       RPDQPEFLAKLLETMPDLRTLSTLHTEKLVVFR-TEHKELLRQQMWSMEDGNNSDGQQNK
Tal-REVERB      RPNDPSLCQDLLSTLNDLWILNGMHS-----------KQTQAKQKGSNSDCDGRMDVSGP
Blage-E75       HPGHANICHELMKKIPDLRTLNTLHSEKLLAFKMTEQQQLQQQQQQHLWGTSPEEESNS
Apido-E75       HPQHPDILRELLKKIPDLRTLNTLHSEKLLAFKMTEQQQQMQAQQQHQ------------
                .*  ..:  .*:..: **. *. :*:           ::    *

Drome-E75       SPSG------SWADAMDVEAAKSPLGSVSSTESADLDYGSPSSSQPQGVSLPSPPQQQPS
Tal-REVERB      QTEGFSSQYPCYSDYPEVRGCPMRYSNAS-------EYDEK----------RAPMEEQTS
Blage-E75       KSPAGSSSWSSSSDVTMDEAVKSPLGSVS-------STESVCSGEVASLTEYQPNHHPVS
Apido-E75       ------------------------------------------------------------

Drome-E75       ALASSAPLLAATLSGG-CPLRNRANSGSSGDSGAAEMDIVGSHAHLTQNGLTITPIVRHQ
Tal-REVERB      QTSPTSPSSSTFCPDEEMRSPGSADSGCSTDSGCSIESGCSSDSG---CSMNIASN----
Blage-E75       HQASSAPLLAATLAGGICPHRHRANSGST--SGDDDMSGLPHHSH---HGLTITAV----
Apido-E75       ------------------------------------------------------------

Drome-E75       QQQQQQQQIGILNNAHSRNLNGGHAMCQQQQQHPQLHHHLTAGAARYRKLDSPTDSGIES
Tal-REVERB      ----------------------DHARSTLLSDEPR------------ETMSVPDDETTER
Blage-E75       -----------------------NPPSRQQPLMPQHHHRFQ------RKLDSPSDSGIES
Apido-E75       ------------------------------------------------------------

Drome-E75       GNEKNECKAVSSGGSSS-----CSSPRSSVDDALDCSDAAANHNQVVQHPQLSVVSVSPV
Tal-REVERB      FRKCYIKRKPN-----------VEESRIPLEK----------------------------
Blage-E75       GTEKLDKLTSGGGSTGSAPTSVCSSPRSSLED----------------------------
Apido-E75       ------------------------------------------------------------

Drome-E75       RSPQPSTSSHLKRQIVEDMPVLKRVLQAPPLYDTNSLMD------EAYKPHKKFRALRHR
Tal-REVERB      GRPMTWHKPQKESEKLADSPVLRMCLEAPSTLKMDSFKDVYRGHSAHSHPHKKFRPTVDR
Blage-E75       KDEEKHHNGSSGSSHIDDMPVLKRVLQAPPLYDTNSLMD------EAYKPHKKFRACRNK
Apido-E75       ------------------------------------------------------------

Drome-E75       EFETAEADASSSTSGSNSLSAGSPRQSPVPNSVATPPPSAASAAAGNPAQSQLHMHLTRS
Tal-REVERB      ---------SGHPHETLDYSHSSPRPSTTPSPSYSSSPSPSSSISSSPSRIS----SSPY
Blage-E75       ---------DSAEAEPMIVHVSPPPPSHPVPPQHHSSPQLHLHLTSNNHQSQ----SSTS
Apido-E75       ------------------------------------------------------------

Drome-E75       SPKASMASSHSVLAKSLMAEPRMTPEQMKRSDIIQNYLKRENSTAASSTTNGVGNRSPSS
Tal-REVERB      SSSSSSNPSSPANVSILAHRLAMPCKKFPKTLSLVQSLSQESK---------FGTGKALV
Blage-E75       STSSSLSSTHSTLAKSLMESPRMTAEQLKRTDIIHNYIMRADSPNPVTIEFPPSAPSPSA
Apido-E75       ------------------------------------------------------------

Drome-E75       SSTPPPSAVQNQQRWGSSSVITTTCQ----------QRQQSVSPHSNGSSSSSS------
Tal-REVERB      SDTLHDCIMNDQSKIPRQHVLATASPS-------RYDCGRNSSPLRSQSYMSSP------
Blage-E75       NSASTTSTSSYKMNSGNLLVCANSTPSTGYHYIPQIQPQQQQQQTVVGRWQGSPGFSNGA
Apido-E75       ------------------------------------QQQQQTQHVINVGW----------
                                                    :  .. .
```

```
Drome-E75          --------------------SSSSSSSSSSS--------------------TSSNCSSSS
Tal-REVERB         --------------------THSMTTSPPPH--------------------D--DVPPHS
Blage-E75          SVITTTTGRNPTPQQTYVLLQNSTNISPPVHHAEMQQSEFSRIYFHPGNAVSPHHAAASS
Apido-E75          ------------------------------------------------------------

Drome-E75          ASSCQYFQSPHSTSNGTSAPASSSSGSNSATP-------LLELQVDIADSAQPLNLSKKS
Tal-REVERB         YSSPIPVAHSSPSHTPTGLGASAMSGCNFSSS-------------YICDNAEPLNLSTKT
Blage-E75          STSPSPLPIPHRKTPPAVASCPPSPSSSGVTPIVISSPKMMELQVDIADSQQPLNLSKKS
Apido-E75          ------------------------------------------------------------

Drome-E75          PTPPPSKLHALVAAANAVQRYPTLSADVTVTASNGGPPSAAASPAPSSSPPASVGSPNPG
Tal-REVERB         PSPPPA------------------------------------------------------
Blage-E75          PSPSPH------------------------------------------------PMASPA
Apido-E75          ------------------------------------------------------------

Drome-E75          LSAAVHKVMLEA
Tal-REVERB         ---------MET
Blage-E75          TTVTHKVVSLEA
Apido-E75          ------------
```

Figure S20. Putative *Talitrus saltator* REVERB protein

Alignment of *Drosophila melanogaster* E75 (Drome-E75; Accession No. NP_730321) with the *T. saltator* (Tal-REVERB) deduced from the Trinity *de novo* transcriptome assembly, together with the top two tblastn species homologue sequences *Blattella germanica* E75 (Blage-E75; Accession No. AM710419) and *Apis dorsata* E75 (Apido-E75; Accession No. XM_006618394). '*' indicates identical amino acid residues in the two proteins, '.' and ':' indicate similar amino acid residues between the two proteins. In this figure SMART identified domains consisting of one C4 zinc finger domain and one HOLI ligand binding domain are highlighted in yellow and green respectively.
