# Supplementary material for: Identification and temporal expression of putative circadian clock transcripts in the amphipod crustacean Talitrus saltator
Source: PeerJ. 2016 Oct 5;4:e2555. doi: 10.7717/peerj.2555 (PMC5068443; doi:10.7717/peerj.2555)
Supplement: Figure S21 — Alignment of Drosophila melanogaster SIR2 (Drome-SIR2; Accession No. NP_477351) with the T. saltator (Tal-SIRT1) deduced from the Trinity de novo transcriptome assembly, together with the top two tblastn species homologue sequences Microplitis demolitor SIRT1 (Micde-SIRT1; Accession No. XM_008555996) and Fopius arisanus SIR2 (Fopar-SIR2; Accession No. XM_011303457). ’*’ indicates identical amino acid residues in the two proteins, ’.’ and ’:’ indicate similar amino acid residues between the two proteins. In this figure one SMART identified SIR2 domain is highlighted in yellow. [file peerj-04-2555-s021.pdf]

```
Drome-SIR2        ------MMENYEEIRLGHIR--SKDLGNQVPDTTQFY--PPTKFDFGAEILASTSTEAEA
Tal-SIRT1         --EGLRVMDSTVPAKRLRLDYSGEMLANEQKESFQYYIRTPDMLSTGG-RVSSTSSTSSV
Micde-SIRT1       MASGSELPEYSSSVKRRKIE------------GQVYRVSEPLDLDFSESKQPRQSSSSDL
Fopar-SIR2        MASGSELPEYSSQVKRRKIE------------SKVYGVSVHFNIDFPEVKVNRDTQNIDI
                        : :     .  .:                :       :.         :   .

Drome-SIR2        EAEATATTTEPATSEL------------------------AGKANGEIKTKTLAAREEQE
Tal-SIRT1         AMEGGSLTCDSGFNELTPPRLLEEEPQSQLSSVSGSLSSHSPQPHGIKRSHASCIDEDDE
Micde-SIRT1       E---EPLSGDSGFTEL------------------------S---DGSKSMSTTP------
Fopar-SIR2        E---EPLGGDSGFTDM------------------------S---DGAKSMSNTP------
                       .   :.. .::                        :    *

Drome-SIR2        IGA------------------NLEHKTKNPT-----KSMGEDEDDEEEEEEDDEEEE---
Tal-SIRT1         ETAPPARRSPVASLSLGAQRSSMSHCSNSPSPSHHFNHHSSLDGGDPDDANDGDDN--S
Micde-SIRT1       ---------------------DLMHLSSTPS-----RTDSTSDDTGCPLDTADEKDEVSS
Fopar-SIR2        ---------------------ESTNLSSTPS-----RTDSTSDDTGCPLDTADEKDEVSS
                                       .  : :..*:     ..   . *     :  * .::

Drome-SIR2        -EDDEEGITGTSNEDEDSSSNCSSSVEPDWK---LRWLQREFYTGRVPRQVIASIMPHFA
Tal-SIRT1         VSSDDSRVSRLSNGDDDAAGGEGSDCGEEGTPEPAHWVQQQMRRGQNPRAVLSRVLGADP
Micde-SIRT1       TVSNLSDLSGLSELSEDS--------GHLWR-NASSWVQKQMITGANPRELLQHLL-MDP
Fopar-SIR2        TVSNLSDLSGLSELSEET--------GQLWR-SASAWVQKQMQTGTDPRDLLQHLL-MDP
                    .: . ::  *: .:::                  *:*.::  *  **  ::  ::   .

Drome-SIR2        TGLAGDTDDSVLWDYLAHLLNEPKRRNKLASVNTFDDVISLVKKSQKIIVLTGAGVSVSC
Tal-SIRT1         SDIPEHVSDEMIWRLLLNVLTDGPRRERLRHLNTLDDAVRLMRTSRRIIVLTGAGVSVSC
Micde-SIRT1       TQIPEQVDDITLWKLVLNIMSDPPRRHKLEHINTLADVVRLIKNSKKIIVLTGAGVSVSC
Fopar-SIR2        TQIPEQVDDITLWKLVLNIMSDPPRRQKLSYVNTLNDVVRLIKDSKKIIILTGAGVSVSC
                  : :.  ..*  :*  : :::.:  **  .*  :**: *.: *:. *..**:**********

Drome-SIR2        GIPDFRSTNGIYARLAHDFPDLPDPQAMFDINYFKRDPRPFYKFAREIYPGEFQPSPCHR
Tal-SIRT1         GIPDFRSRDGIYARLAVDFPNLPDPQAMFDINFFKRDPRPFFKFAREIYPGQFTPSLCHR
Micde-SIRT1       GIPDFRSRDGIYSRLAQDFPDLPDPQAMFDINYFGQDPRPFFKFAREIYPGQFKPSPCHR
Fopar-SIR2        GIPDFRSRDGIYSRLAQDFPNLPDPQAMFDINFFAQDPRPFFKFAREIYPGLFKPSPCHR
                  ******* :***:*** ***:***********:* .*****:********* * ** ***

Drome-SIR2        FIKMLETKGKLLRNYTQNIDTLERVAGIQRVIECHGSFSTASCTKCRFKCNADALRADIF
Tal-SIRT1         FIRSMENHNKLLRNYTQNIDTLEQVAGINNVIQCHGSFASATCQRCGFKVSAGAIKDDIF
Micde-SIRT1       FIKMLDKKNKLLRNYSQNIDTLEQVAGIENVIECHGSFATASCTKCKYQVTAEDIREDIF
Fopar-SIR2        FIKMLEKHKKLLRNYSQNIDTLEQVAGIENVIECHGSFATASCTKCKFQVTAEDIRTDIF
                  **. ::.: ******:*******.****:.**:*****::*:* .* :: .*  :. ***

Drome-SIR2        AQRIPVCPQCQPNKEQ--------------------------------------------
Tal-SIRT1         QQRIPMCPHCTPPTTPLPCYGPRDSFYGHHHGGDTPTPPLQSSASLQASPARNESTSPQP
Micde-SIRT1       AQRIPLCPKCYTNSLP--------------------------------------------
Fopar-SIR2        SQRIPLCPKCSTNSLP--------------------------------------------
                   ****:**:* . .

Drome-SIR2        ----------------------------------SVDASVAVTEEELRQLV-ENGIMKPD
Tal-SIRT1         GAAGSESPQQATGASHNLNLADSPAHNDDDSNLETFNLATLCARSDSSNLMQSQPIMKPD
Micde-SIRT1       ------------------------------------SLSSTNTNDNYANFV-SQGIMKPD
Fopar-SIR2        ------------------------------------SLATTNTSQNYTDLV-SLGIMKPD
                                                      . :   : .:  ::: .  *****

Drome-SIR2        IVFFGEGLPDEYHTVMATDKDVCDLLIVIGSSLKVRPVAHIPSSIPATVPQILINREQLH
Tal-SIRT1         IVFFGEGLPDEFHDSISEDQNQCDLLIVIGSSLKVRPVAHIPNSIPPHVPQILINREPLD
Micde-SIRT1       IVFFGEGLPDAFHDAIANDKDECDLLIVIGSSLKVRPVALIPSSIPSYVPQILINRESLP
Fopar-SIR2        IVFFGEGLPDAFHDSIAKDKDECDLLIVIGSSLKVRPVALIPSSIPSHIPQILINRESLP
                  ********** :*  :: *:: ***************** **.***. :******** *

Drome-SIR2        HLKFDVELLGDSDVIINQICHRLSDNDDCWRQLCCDESVLTESKELMPPEHSNHHLHHHL
Tal-SIRT1         HLTFDIELLGDCDVIVNELCHRLGPS---WTNVCNSHVRLSEIRDL-PPKPETP------
Micde-SIRT1       HLKFDIELLGDGDIIINQLCHLIGEE---FSEICWKKDILEETPHLLPPRFITD------
Fopar-SIR2        HLKFDVELLGDGDIIINQLCHLMGEE---FLQLCWSEKLLEETPHLLPPRFEDS------
                  **.**:***** *:*:*::** :. .   : ::* .   * *   * **
```

```
Drome-SIR2       LHHRHCSSESERQSQLDTDTQSIKSNSSADYILGSAGTCSDSGFESSTFSCGKRSTAAEA
Tal-SIRT1        ------SSSPASARLVAGDGDASVAGSGVAPVAAV--------ADAPHPKDEDDIEALRA
Micde-SIRT1      ---------DSWERSQDSTINQELSQDSTEVVLKT--------YHHNVSTESQDSLMINS
Fopar-SIR2       -----------WERSTTS------TQDSTEVILKQ----------HNLFNESQDSLSINS
                                         : ...  :                 .  .      :

Drome-SIR2       AAIERIKTDILVELNETTAL--SCDRLGLEGPQTTVESYRHLSIDSSKDSGIEQCDNEAT
Tal-SIRT1        CWAPKIRESVAARLPENCYLYNGGHRYVFKGAEVFYDPDDVEGDDDSQNQEDDDDDDDED
Micde-SIRT1      NTTPKRLENTDICISP----FHAGH---MEATEESFA-----LLGESPKRPLGDSSLESS
Fopar-SIR2       NTTPKHLGSTEICISP----FDAGH---MEGNDGF-------SLLESPKRPLDESSVESS
                     .   .    :        .     ::. :            .* .    : . :

Drome-SIR2       PSYV-----------R----PSNLVQETKTVAPSLTPIPQ--QRGKRQTAAERLQPGTFY
Tal-SIRT1        GELVDNDDDSSPPLHE----ASNSLNPKLVSGDALSGIPSEKEFLPSFSLQNTKEPATGV
Micde-SIRT1      PKRINLEVRASPPSSSGSSGSSGSISRSLDDSQDINYTTS--RYNRVISVESTSENNGHI
Fopar-SIR2       PKRLN---------SR----SCGSTPRSSDDGERF-------RFTRVVSFESTPENNGQV
                  . :                ...    .   .  :             :  .  :

Drome-SIR2       SHTNNYSYVFPGAQVFWD-------------------NDYSDDDDEEEERSHNRHSDLFG
Tal-SIRT1        SPPHSLDDVVEAVQAVEEA----------DQLLYSSCDTITHSDCTR--RTLSCDSSASS
Micde-SIRT1      ---YNLEECHVVPRIIDDSTLKNSISTLDDLTQSNNSNTPVDVDSSNDSEELLDSDKSNG
Fopar-SIR2       ---FNLEECHVLPRILDQ-------------------DSGVKGEERRDERE---GEDKYG
                     . .      . . :                   :     :           ..  .

Drome-SIR2       NVGHNYKDDDE-----DACD-LNAVPLSPLLPPSLEAHIVTD---IVNGSNEPLPNSSPG
Tal-SIRT1        SSCHLSTAATV------TVEAPSVQEMDSLLSGSEQEEDCVDSD-WM-----TLHASLAD
Micde-SIRT1      KSRHISIDSAIDSGLGDSCNSVDSSDDKSTIEQGDSKRHNLQRHCWQPEVKESLASRLPE
Fopar-SIR2       KSRHASIDSAIDSGIGDSCNSVDSSEAKEI----EEKRQEIERR-WQGEIKESLANRLPE
                 .  *             : :  .    .       .     :          .*    .

Drome-SIR2       QKRTACIIEQQPTPAIETEIPPLKKRRPSEENKQQTQIERSEESPPPGQLAAV
Tal-SIRT1        TPSFSSDVAAPTLRPSEDCSRPESQPQFNEPSESAPKLNDKIESISRTSVAL-
Micde-SIRT1      NTYYQVSPGKYIFTGAEIYIEPDDYEQLSSSSSRSPSMSTMIRPATEYNTT--
Fopar-SIR2       GSYYQVSSGKYIFPGAEIYLEGDD---FGEPETRANEISSLTYTEDECRTT--
                                 *      .    .. .    .:.    .      :
```

Figure S21. Putative *Talitrus saltator* SIRT1 protein

Alignment of *Drosophila melanogaster* SIR2 (Drome-SIR2; Accession No. NP_477351) with the *T. saltator* (Tal-SIRT1) deduced from the Trinity *de novo* transcriptome assembly, together with the top two tblastn species homologue sequences *Microplitis demolitor* SIRT1 (Micde-SIRT1; Accession No. XM_008555996) and *Fopius arisanus* SIR2 (Fopar-SIR2; Accession No. XM_011303457). '*' indicates identical amino acid residues in the two proteins, '.' and ':' indicate similar amino acid residues between the two proteins. In this figure one SMART identified SIR2 domain is highlighted in yellow.
