# Supplementary material for: Identification and temporal expression of putative circadian clock transcripts in the amphipod crustacean Talitrus saltator
Source: PeerJ. 2016 Oct 5;4:e2555. doi: 10.7717/peerj.2555 (PMC5068443; doi:10.7717/peerj.2555)
Supplement: Figure S22 — Alignment of Drosophila melanogaster SIRT2 (Drome-SIRT2; Accession No. NP_650880) with the T. saltator SIRT2 (Tal-SIRT2) deduced from the Trinity de novo transcriptome assembly, together with the top two tblastn species homologue sequences Tribolium castaneum SIRT2 (Trica-SIRT2; Accession No. XM_963962) and Branchiostoma floridae hypothetical protein (Brafl-HYPO; Accession No. XM_002596789). ’*’ indicates identical amino acid residues in the two proteins, ’.’ and ’:’ indicate similar amino acid residues between the two proteins. In this figure one SMART identified SIR2 domain is highlighted in yellow. [file peerj-04-2555-s022.pdf]

```
Drome-SIRT2      --------MDKVRRFFANTLHLG-GSSDAKEEVKVEK-----------------------
Tal-SIRT2        -------------KSFAMASASPKRDADAQDSPDPQKPEKAVDDSGVFDASSSEETTAGA
Brafl-HYPO       --------VEHLRRLFARTLGLSQERETEEGTARPQQ-----------------------
Trica-SIRT2      MLKRCKSLLNSTSNLYKTVLKMSRDSSKDRAEAKPKQPP---------EAQSEEDNASGT
                              . :  .                ::

Drome-SIRT2      ----------------------------------VIPDLSFDGFAEHWRVHGFRKIVTMV
Tal-SIRT2        SATDDDSGVFESLMSLIEQKLNFKKTDEEPQ---ILSSLDVDGIVDFMKSDKCHNVITLA
Brafl-HYPO       ----------------------------------VLDEVTVEGIAKYITDGKCKNIIVLT
Trica-SIRT2      SMS------IDSLRKYLAEKLGISDKDDEKEKIKILDDVSVDGIAVYIKKKNCKNIITMA
                                                   :: .: .:*:. .      .:::.:.

Drome-SIRT2      GAGISTSAGIPDFRSPGSGLYSNLKKYELPHPTAIFDLDYFEKNPAPFFALAKELYPGSF
Tal-SIRT2        GAGISTSAGIPDFRSPGSGLYNNLQKYNLAYPEAIFELGFFRENPQPFFTLAKELYPGSF
Brafl-HYPO       GAGISTSAGIPDFRSPGTGLYDNLQKYNLPNPHAIFEIGFFKENPEPFFALAKELYPGKF
Trica-SIRT2      GAGISTSAGIPDFRSPGSGLYDNLQKYNLPHPQAIFELDFFHENPKPFFTLAKELYPGSF
                 *****************:***.**:**:*. * ***::.:* :** ***:********.*

Drome-SIRT2      IPTPAHYFIRLLNDKGLLQRHYTQNIDTLDRLTGLPEDKIIEAHGSFHTNHCIK-CRKEY
Tal-SIRT2        NPTPCHFFIKLLEQKGLLLRHYTQNIDTLEHVAGISEEKLIEAHGSFRTAHCLG-CRKSY
Brafl-HYPO       KPTWCHYFIKLLSDKGLLLRNFTQNIDTLERVAGVSAGAMVEAHGTFYTAHCLGECRKEY
Trica-SIRT2      KPTISHYFIKLLIEKDVLLRHYTQNIDTLERIAGIPEEKIVEAHGTFYTGHCLA-CRKEY
                  ** .*:**.** :*.:* *::*******:.::*:.   ::****:* * **:  ***.*

Drome-SIRT2      DMDWMKAEIFADRLPKCQ--KCQGVVKPDIVFFGENLPKRFYSSPEEDFQDCDLLIIMGT
Tal-SIRT2        DQQWIKDEVFADRVPTCE--SCGSVVKPDIVFFGEGLPDKFFGAVRSDFRKCDLLIVMGT
Brafl-HYPO       TQEWVKEKVFNDEVPRCP--DCDGVVKPDIVFFGEAMPAKFFPSVLADFPRCDLLIVMGT
Trica-SIRT2      QLEWMKERIFKDEVPECESKDCKGVVKPDIVFFGEALPDKFYSLIENDFKKCDLLIILGS
                   :*:*  :* * :* *   .* .*********** :* .*:     **  *****::*:

Drome-SIRT2      SLEVQPFASLVWRPGPRCIRLLINRDAV---GQASCVLFMDPNTRSLLFDKPNNTRDVAF
Tal-SIRT2        SLTVQPFASLIDNVSSTCPRLLINRDAVGPTGAAATIQRLMGHGSGFQPDSPKNKRDVAL
Brafl-HYPO       SLQVQPFASLVDRVPETCPRLLINREKC---GQVDPIMRMLGFGGGMEFDSENNYRDVAW
Trica-SIRT2      SLVVQPFASLVDRVLDTCPRLLINREKV---NNGSGIMAMFGFGAGFDFDGKNNTRDVAW
                 ** *******: .    * ******:     .    :  :     .:  *  :* ****

Drome-SIRT2      LGDCDAGVMALAKALGWDQELQQLITSERKKLSGSQNSEELQQGKEKPQSDPDKMTSGDR
Tal-SIRT2        LGDCDDGCLMLAEKLGWKEDLQKLIEEGKKKPDAT-------------------------
Brafl-HYPO       LGDCDEGCKALAELLGWKEQLEELVHREHTRIEAQ-------------------------
Trica-SIRT2      IGDCDEGCQLLADKLGWGDELKKLRQTEIEKIEKA-----------------------EA
                 :**** *   **. *** ::*::*      . .

Drome-SIRT2      DKKDASL
Tal-SIRT2        -------
Brafl-HYPO       -------
Trica-SIRT2      TPVKSSM
```

Figure S22. Putative *Talitrus saltator* SIRT2 protein

Alignment of *Drosophila melanogaster* SIRT2 (Drome-SIRT2; Accession No. NP_650880) with the *T. saltator* SIRT2 (Tal-SIRT2) deduced from the Trinity *de novo* transcriptome assembly, together with the top two tblastn species homologue sequences *Tribolium castaneum* SIRT2 (Trica-SIRT2; Accession No. XM_963962) and *Branchiostoma floridae* hypothetical protein (Brafl-HYPO; Accession No. XM_002596789). '*' indicates identical amino acid residues in the two proteins, '.' and ':' indicate similar amino acid residues between the two proteins. In this figure one SMART identified SIR2 domain is highlighted in yellow.
