# Supplementary material for: Identification and temporal expression of putative circadian clock transcripts in the amphipod crustacean Talitrus saltator
Source: PeerJ. 2016 Oct 5;4:e2555. doi: 10.7717/peerj.2555 (PMC5068443; doi:10.7717/peerj.2555)
Supplement: Figure S24 — Alignment of Drosophila melanogaster SIRT6 (Drome-SIRT6; Accession No. NP_649990) with the T. saltator SIRT6 (Tal-SIRT6) deduced from the Trinity de novo transcriptome assembly, together with the top two tblastn species homologue sequences Papilio polytes SIRT6 (Pappo-SIRT6; Accession No. XM_013284914) and Papilio xuthus SIRT6 (Papxu-SIRT6; Accession No. XM_013314262). ’*’ indicates identical amino acid residues in the two proteins, ’.’ and ’:’ indicate similar amino acid residues between the two proteins. In this figure one SMART identified SIR2 domain is highlighted in yellow. [file peerj-04-2555-s024.pdf]

```
Drome-SIRT6     -----------MSCNYADGLSAYDNKGILGAPESFDSDEVVAEKCQELAELIKKSGHVVL
Tal-SIRT6       FTTFYCFHRFAMSCNYAEGLSPYPDKGQVGMPERFAGKEEVSRKVSELAALMKASRHTVF
Pappo-SIRT6     -----------MSCNYAEGLSPYEHKGVLGIPEKFEAIEKLNEKCEMLARLITESKHIVV
Papxu-SIRT6     -----------MSCNYAEGLSPYEHKGVLGIPEKFEAIEKLNEKCELLARLISESKHIVV
                           ******:***.*  ** :* ** * . * :  * . ** *:. * * *.

Drome-SIRT6     HTGAGISTSAGIPDFRGPKGVWTLEEKGEKPDFNVSFDEARPTKTHMAIIALIESGYVQY
Tal-SIRT6       HTGAGISTSAGIPDFRGPKGVWTLEKKGLRPEVNVSFDDARPTYTHMALVALEKAGLIHY
Pappo-SIRT6     HTGAGISTSAGIPDFRGPNGVWTLEKEGKRPSTNISFTDAKPTKTHMILKKLIESNKIQY
Papxu-SIRT6     HTGAGISTSAGIPDFRGPNGVWTLEKEGKRPSTNISFTDAKPTKTHMILKKLIDCNKIQY
                ******************:******::* .*. *:** :*.** *** :  * ... ::*

Drome-SIRT6     VISQNIDGLHLKSGLDRKYLSELHGNIYIEQCKKCRRQFVSPSAVETVGQKSLQRACKSS
Tal-SIRT6       LVTQNIDGLHLRSGFPRRKMAELHGNMYLDKCSVCKREFVRCTAVSTVGQKSLGVGCPGK
Pappo-SIRT6     IVSQNIDGLHMKSGLSRKYLSELHGNMFIDECSLCKRQFIRSSPVETVGKKCSGVPCASG
Papxu-SIRT6     IVSQNIDGLHLKSGLSRKYLSELHGNMFIDECSLCKRQFIRSSPVETVGKKCSGVPCASG
                :::*******:.**: *. ::*****:::::*. *.*:*:  :.*.***:*.    * .

Drome-SIRT6     MDSKGRSCRSGILYDNVLDWEHDLPENDLEMGVMHSTVADLNIALGTTLQIVPSGDLPLK
Tal-SIRT6       RDT-GRRCR-GRLHDNILDWEHDLPHLDYNLAEKHSVGSDLSVCLGTTLQIIPSGVLPTL
Pappo-SIRT6     YNG-GRPCR-GRLYDGVLDWEHSLPEDDLLMAEWHSSIADLSICLGTTLQIVPSGNLPLE
Papxu-SIRT6     HNG-GRPCR-GRLYDGVLDWEHSLPENDLLMAEWHSSIADLSICLGTTLQIIPSGNLPLE
                 :  ** ** * *:*.:*****.**  *  :.  **  :**.:.*******:*** **

Drome-SIRT6     NLKCGGKFVICNLQPTKHDKKANLIISSYVDVVLSKVCKLLGVEIPEYSEASDP------
Tal-SIRT6       AKQSGGKLVICNLQPTKHDKISDIIINGYVDDVMRELLSLLNITCPEYSMEQDPVLITRA
Pappo-SIRT6     TVKYGGKLVICNLQPTKHDNKSDLIINYYVDDILEKVMNILKLDIPEYNEDTDL------
Papxu-SIRT6     TVKYGGKLVICNLQPTKHDNKADLIINYYVDDILEKVMNILNLDIPQYNEDTDL------
                  : ***:***********: :::**. *** :: :: .:* :  *:*.   *

Drome-SIRT6     --TKQSKP-----MEWTIPTS---NVNTFHRQYK------------KYVKDSKIESKAKK
Tal-SIRT6       LKTKLACSSDAELIEWTIPDSWFDDKDLIEKVSRNKAIADQKNAGKKYPRATKIDRRKRK
Pappo-SIRT6     --TKLAST---EIVEWTIKKK---DVLVLEKVFKAKC---------KGVKKKKILIKTKR
Papxu-SIRT6     --TKLAST---SIVEWTINKK---DVLVLEKVFKAKC---------KGVKKKKILIKNKR
                  ** : .     :****  .   :   : .  .            *  . .**  . ..

Drome-SIRT6     T--------------------------KYT--------------
Tal-SIRT6       SPIKPEVKKEETGESSSQDEGLSARNERYRKRSLDAGQLGTNKR
Pappo-SIRT6     N--------------STESNGIENGNCKVIKLEVKQECDISEIK
Papxu-SIRT6     N-------------STEINNGIENGNCKVIKLEIKDECV-----
                .                          .
```

Additional file 29. Putative *Talitrus saltator* SIRT6 protein

Alignment of *Drosophila melanogaster* SIRT6 (Drome-SIRT6; Accession No. NP_649990) with the *T. saltator* SIRT6 (Tal-SIRT6) deduced from the Trinity *de novo* transcriptome assembly, together with the top two tblastn species homologue sequences *Papilio polytes* SIRT6 (Pappo-SIRT6; Accession No. XM_013284914) and *Papilio xuthus* SIRT6 (Papxu-SIRT6; Accession No. XM_013314262). '*' indicates identical amino acid residues in the two proteins, '.' and ':' indicate similar amino acid residues between the two proteins. In this figure one SMART identified SIR2 domain is highlighted in yellow.
