# Supplementary material for: Identification and temporal expression of putative circadian clock transcripts in the amphipod crustacean Talitrus saltator
Source: PeerJ. 2016 Oct 5;4:e2555. doi: 10.7717/peerj.2555 (PMC5068443; doi:10.7717/peerj.2555)
Supplement: Figure S25 — Alignment of Drosophila melanogaster SIRT7 (Drome-SIRT7; Accession No. NP_651664) with the T. saltator SIRT7 (Tal-SIRT7) deduced from the Trinity de novo transcriptome assembly, together with the top two tblastn species homologue sequences Megachile rotundata SIRT7 (Megro-SIRT7; Accession No. XM_012287821) and Apis dorsata SIRT7 (Apido-SIRT7; Accession No.). ’*’ indicates identical amino acid residues in the two proteins, ’.’ and ’:’ indicate similar amino acid residues between the two proteins. In this figure one SMART identified SIR2 domain is highlighted in yellow. [file peerj-04-2555-s025.pdf]

```
Drome-SIRT7      MEKDLGEEKDQDQEQNTEMEPKQEMDVAQSYITRAKMNPAKKDNEKRRRKDAMRRVSMIL
Tal-SIRT7        ---------VLMDERKTTVNMMDEEGDGVVENAKRKAATNATLNLSKKSSSKMKRVTWLL
Megro-SIRT7      -----------------------MEEAASEKFLSRRRSAALKAFKVKDERVATFKKVAAIL
Apido-SIRT7      -----------------------MEETSNEKFLSRRRSATLKAFKVKDERIATFKKVAAIL
                                        *        :. .  .    : .     :..*: :*

Drome-SIRT7      RKCDSMR--TTEDRQFLEKHPDMVKTTKKRKERVEIYKERVVEREDAPHVIEAKVEQLAN
Tal-SIRT7        RKRQEHKQLSNDEQKFLADNPKLVDQFNKRREKRQEYQSRHIEKEDSPETLREKCAELAQ
Megro-SIRT7      QKSETDR--TAEETGILVSCSDVVKEVNLRQEKRHRVKARLEEVEDAPEILEEKCIRLAA
Apido-SIRT7      QKSETDR--TAEEIGILISCSDIVKEVNLRQEKRHRVKARLEEIEDAPELLEEKCMRLAA
                 .* :   .  : ::  :* . ..:*.  : *.*.    : *  * **:*  :  *   **

Drome-SIRT7      IISQAKHLVCYTGAGISTAALIPDYRGSQGIWTLLQKGQDIGEHDLSSANPTYTHMALYE
Tal-SIRT7        ALHDAEFLVVYTGAGISTSANIPDYRGPNGIWTRLQKGEDIGSHDLTEAEPTVTHMALSA
Megro-SIRT7      AISRATSLAIYTGAGISTAASIPDYRGTNGVWTRLQQGKDIGNHDLSQAEPTVTHMALYA
Apido-SIRT7      AISRATSLAVYTGAGISTAASIPDYRGTNGVWTRLQQGKDIGNHDLSQAEPTLTHMALYA
                  :  *  *. ********:* ******.:*:** **:*:***.***:.*:** *****

Drome-SIRT7      LHRRRLLHHVVSQNCDGLHLRSGLPRNSLSEIHGNMYVEVCKNCRPNSVYWRQFDTTEMT
Tal-SIRT7        LQKTGRLQHIVSQNCDGLHLRSGLPKTALSEVHGNMYIEVCRGCVPAREYVRTFDVTERT
Megro-SIRT7      LYKARVLKHIVSQNCDGLHLRSGIPRTLLSEVHGNMYIEVCRTCKPYREYWRLFDVTEKT
Apido-SIRT7      LYKARVLKHIVSQNCDGLHLRSGIPRTLLSEVHGNMYVEVCRICKPYREYWRLFDVTEKT
                 * .   *:*:*************:*.. ***:*****:***. * *   * * **.** *

Drome-SIRT7      ARYCHKTHRLCHRCSEPLYDTIVHFGERGNVKWPLNWAGATANAQRADVILCLGSSLKVL
Tal-SIRT7        STYKHATGRRCYRCGEPLVDSIVHFGERGKLRWPLNWQGACQAANAADTILCLGSSLKVL
Megro-SIRT7      ARYSHGTGRLCHRCNSVLHDSIVHFGERGNLPWPINWNGATRAAKQADVILCLGSSLKVL
Apido-SIRT7      ARYSHGTGRLCHRCNSVLQDSIVHFGERGNLPWPINWNGATRAAKQADVILCLGSSLKVL
                 : * * * * *:**.. * *:********:: **:** **   *: **.***********

Drome-SIRT7      KKYTWLWQMDRPARQRAKICVVNLQWTPKDAIASIKINGKCDQVMAQLMHLLHIPVPVYT
Tal-SIRT7        KKYTWLWQMDRPARRRPRLYVVNLQWTPKDKEATMKINGKCDEVMKLVMDYMGIEIPRYN
Megro-SIRT7      KKYPWLWQMDRPIHKRPSLYIVNLQWTPKDENAVLKINGKCDEVMKRVMVHLGLEIPQYD
Apido-SIRT7      KKYPWLWQMDRPIHKRPSLYIVNLQWTPKDENAVLKINGKCDEVMKRIMTHLGLEIPQYN
                 ***.********  ..*. : :*********  *  :*******:**  :*  : : :* *

Drome-SIRT7      KEKDPIFAHASLLMPEELHTLTQPLLKN--------ADEEEAFTTTTEETQDSTISSESC
Tal-SIRT7        RSEDLIFDMATPLHPDELRTTTRPSLVPPIDLPIPIEESSNNLEENVTERNKSPVSVETC
Megro-SIRT7      RTKDPIFFHAVRLRSNEQQTTSQPCLEEPANM---TLKEIDEITDNFHEDIASETKEEDC
Apido-SIRT7      RAKDPIFFHAVRLRNSEQHTTSQPCLEEPTNI---VHKELNQIDDNFHEDISSSTKEKEC
                 . :* **  *  *  .* .* :.* *             .. : :  .  *   *  . : *

Drome-SIRT7      SFNYS-------DLPIGKGPRIRTPIKNGRRVKTNLELRQKFKTLNGQDEEIKVEHVKTN
Tal-SIRT7        QNSRRFDRDDLGKNEVSVDERCSKVLNCAPVCNSSGDRISKYDGISASTMTNDIECGETI
Megro-SIRT7      ASPIK-------------------------------------------------------
Apido-SIRT7      ISSKR-------------------------------------------------------

Drome-SIRT7      GEVKTEKDLITLESSIKIETEVKLEKLECSDTNFQQELK---------------------
Tal-SIRT7        IELPSVAQCKNGISLEKLKHFTREDNLNCCSSQTCCHTRSQYSDILQVNSKCSNYDSRLC
Megro-SIRT7      ------------------------------------------------------------
Apido-SIRT7      ------------------------------------------------------------

Drome-SIRT7      ------------------------------LLELLPKLEPLSLKEETEETPSNGFPELPK
Tal-SIRT7        ISTSDSVVRTLATSTASVVVTRVSRSPCAVSWESVPAEVLSETCSSQSDNPGSEFVHESS
Megro-SIRT7      ------------------------------------------------------------
Apido-SIRT7      ------------------------------------------------------------

Drome-SIRT7      LVAIQKTHAECLSAVPTESRLKPLQLPPLVPIGAPLSTPFVEPKLVLPPASQSSS-----
Tal-SIRT7        VSPSVEINTSSVTLSSSTSCSPSYSTVATPRMTTTLEGEISGPGLAPSKCEHCTTGSCIH
Megro-SIRT7      -----------ISQNPS--------------------------QLVP-------------
Apido-SIRT7      -----------VTESMSAYSAPFFTALPFLSMGLPFPPMYMCPQLTP-------------
                            ::    :                          *.
```

```
Drome-SIRT7      ---IQIKSEGDGDSSTENDNEEEEESELAQM-------DLLRQNNDEELLRQLP------
Tal-SIRT7        CEVLRLFSGMDGCELLSGDQCCKCEVVMKKMFSCGAAPKLMKCDKCGLFLRFFLLGTCRP
Megro-SIRT7      ------------------------------------------------------------
Apido-SIRT7      -------------------------------------------------LFYYP------

Drome-SIRT7      -------------------------TWYDAKYAYSGLHSILIPPPADLNIWNSQVVPNFA
Tal-SIRT7        ASRNGELNVNGKLNFGCTVSHLFCDCFEKGQLAVNGAKLPSSASGDASVAATAAAAATVT
Megro-SIRT7      ------------------------------------------------------------
Apido-SIRT7      ----------------------------------------------------FVQVPNIT

Drome-SIRT7      --MNRSAAS-CFFCFDRYAELECQFYRRWNLS------QRKHKKRARSGRFVVCECCPTS
Tal-SIRT7        SIIATAAESRSLVTLHVDASELCSQESSGEDSIVMLDCDDMEFKDQKFQDGDIKDIKDEV
Megro-SIRT7      ----------------------SVWRTRGHLL----------------------------
Apido-SIRT7      SDVPKPKPT-CTFCMENEGSLTCLYYQRETDNSTLIGTESKQIKDTK-------------
                                       .

Drome-SIRT7      DDDDDYDENISLAHIAAAETAKRRQQLSTSFPRKLARTQAGWYGKGYKKGRKRR------
Tal-SIRT7        KDVKNFEIFNDACKERKEDIKISKPTFLTNHALSRLSSDHELSKNDFRKNFELHSDSLKT
Megro-SIRT7      -------------------------VFITNEIPIILV-----------------------
Apido-SIRT7      -------------------------TLVIHADPPIAAKNPGWFGKGYRKGMKKKR-----
                                          :

Drome-SIRT7      ---------------------------------------------------------
Tal-SIRT7        EDVKEEKLDVHSSCPVLNFNDTSCKLEKKDENCKLYDSLASSIVNMTSKSKSFCQED
Megro-SIRT7      ---------------------------------------------------------
Apido-SIRT7      ---------------------------------------------------------
```

Figure S25. Putative *Talitrus saltator* SIRT7 protein

Alignment of *Drosophila melanogaster* SIRT7 (Drome-SIRT7; Accession No. NP_651664) with the *T. saltator* SIRT7 (Tal-SIRT7) deduced from the Trinity *de novo* transcriptome assembly, together with the top two tblastn species homologue sequences *Megachile rotundata* SIRT7 (Megro-SIRT7; Accession No. XM_012287821) and *Apis dorsata* SIRT7 (Apido-SIRT7; Accession No.). '*' indicates identical amino acid residues in the two proteins, '.' and ':' indicate similar amino acid residues between the two proteins. In this figure one SMART identified SIR2 domain is highlighted in yellow.
