# Supplementary material for: Identification and temporal expression of putative circadian clock transcripts in the amphipod crustacean Talitrus saltator
Source: PeerJ. 2016 Oct 5;4:e2555. doi: 10.7717/peerj.2555 (PMC5068443; doi:10.7717/peerj.2555)
Supplement: Figure S26 — Alignment of Drosophila melanogaster JETLAG (Drome-JET; Accession No. NP_608880) with the T. saltator JETLAG (Tal-JET) deduced from the Trinity de novo transcriptome assembly, together with the top two tblastn species homologue sequences Orussus abietinus F-box/LRR repeat protein 20 (Oruab-FBOX/LRR; Accession No. XM_012430170) and Harpegnathos saltator F-box/LRR repeat protein 20 Harsa-FBOX/LRR; Accession No. XM_011152298). ’*’ indicates identical amino acid residues in the two proteins, ’.’ and ’:’ indicate similar amino acid residues between the two proteins. In this figure SMART identified domains consisting of one F-box domain and multiple leucine-rich repeat domains are highlighted in yellow and green respectively. [file peerj-04-2555-s026.pdf]

```
Drome-JET             -----------------------------------MCTLHPEEEAHLMAMASGGQPTRTA
Tal-JET               HIILAHLPPIFSHIFWERISRKMKMINQNNINRLELVKVFSEDEALINKKLPRELLLRVL
Oruab-FBOX/LRR        MI-------------------------HSGRTRLELTWVFHDDEAQINKKLPKELLLRIM
Harsa-FBOX/LRR        MI-------------------------HSGRTRLELTWVFHDDEAQINKKLPKELLLRIL
                                                         :  :. ::** :    .     *

Drome-JET             SPRPLVTASIAA--PRSLFDVCWDDVLIPQVAVYLSLKDL------------------FN
Tal-JET               SFLDVVSLCRCAQVSHYWHSLALDGANWQRIDLFNFQTDIEGVVVEQIGRRCGGFLKELS
Oruab-FBOX/LRR        SYLDVVSLCRCAQVSKAWNVLALDGSNWQRIDLFDFQRDVEGPVIENISRRCGGFLRQLS
Harsa-FBOX/LRR        SYLDVVSLCRCAQVSKAWNVLALDGSNWQRIDLFDFQRDVEGPVIENISRRCGGFLRQLS
                      *   :*: . .*  ..    :. *.    .: ::    *:                  :.

Drome-JET             LRCCSRTAQ---RFVEAALEKRQELHLSGNNTKNI-DVAFRVLARCCQRLEVLHLACCRW
Tal-JET               LRGCQSVKDDPLITITSLCRNIERLNLT--NCKNITDLTCGALSRHCPRLLRLELESCSK
Oruab-FBOX/LRR        LRGCQSIGNNSMRTLAQSCPNIEDLNLR--QCKKISDATCAALSSHCPKLQRLNLDSCPE
Harsa-FBOX/LRR        LRGCQSIGNNSMRTLAQSCPNIEELNLS--QCKKISDATCAALSSHCPKLQRLNLDSCPE
                      ** *.   :     :     : : *:*   : *:* * :  .*:  * .*  * * .*

Drome-JET             LTDELLLPL---------------------------------------------------
Tal-JET               VTDQSLQLLADGCPHLVFLNIAWCSEITSAGVEAVAKGCPELEVFVAKGVKELRDQALLQ
Oruab-FBOX/LRR        VTDLSLKSLSDGCPLLTHINLSWCELITDNGVEALARGCPELRSFLSKGCRQLTDRAVKC
Harsa-FBOX/LRR        ITDISLKDLSDGCPLLTHINLSWCELLTDNGVEALARGCPELRSFLSKGCRQLTDRAVKC
                      :**  *  *

Drome-JET             LANNKKRLWAVNLNECVNITALSLQPIIVECKELRVLKLSKCQWLTTGAVDALTLHQSKL
Tal-JET               LASNCPKLRHVNLHSCVLVTDASVTVLAEKCPGLRYLCLSNCSALSDASLQTLALHNTKL
Oruab-FBOX/LRR        LARYCPNLEAINLHECRNITDDAVRELSERCPRLHYVCLSNCPNLTDASLCTLAQHCPLL
Harsa-FBOX/LRR        LARYCPNLEAINLHECRNITDDAVRELSEQCPRLHYVCLSNCPNLTDASLVTLAQHCPLL
                      **    .*  :**:.*  :*  ::  :   *  *. : **:*  *: .:: :*: * . *

Drome-JET             VEFDISYCGAIGERCLIIFFRKLNKLTVLSLANTPSVTDQVLIQIGNYCRELEHINVIGC
Tal-JET               ETLEVANCAQFTDNGFQALARSCRLLERIDLEDCLLITDATLSHLAMGCPRLEKLSLSHC
Oruab-FBOX/LRR        SVLECVACTHFTDAGFQALARNCRLLEKMDLEECVLITDATLIHLAMGCPRLEKLSLSHC
Harsa-FBOX/LRR        SVLECVGCTHFTDAGFQALAKNCRLLEKMDLEECLLITDATLIHLAMGCPRLEKLSLSHC
                        ::    *  : :  :  :  .. . *  :.* :   :** .* ::.  *  **::.:  *

Drome-JET             AAISDYGVHAL---TVHCLRLRTLLIRRCPRVTELSLAPLRQRRLYIDRPQ---------
Tal-JET               ELITDEGIRHLGSAACSTEQLSVLELDNCPLITDASLDHLL-ACHNLHRIELYDCQQITR
Oruab-FBOX/LRR        ELITDEGIRQLALSPCASEHLAVLELDNCPLITDASLDHLLQACHNLQRIELYDCQLITR
Harsa-FBOX/LRR        ELITDEGIRQLALSPCAAEHLAVLELDNCPLITDASLDHLLQACHNLERIELYDCQLITR
                        *:* *:. *   .    .* .* : .** :*: **  *      : * :

Drome-JET             ----------PDVGLNAYNLNDFYPSD-----------FLVY
Tal-JET               AGIRRLKAHLPNIKVQAYFAPQTPPVPENTP-HRLHRCCTIL
Oruab-FBOX/LRR        AGIRRLRTHLPNIKVHAYFAPVTPPPSAGASTQRYCRCCVIL
Harsa-FBOX/LRR        AGIRRLRTHLPNIKVHAYFAPVTPPPSAGASRPRYCRCCVIL
                                *:: ::**      *               :
```

Figure S26. Putative *Talitrus saltator* JETLAG protein

Alignment of *Drosophila melanogaster* JETLAG (Drome-JET; Accession No. NP_608880) with the *T. saltator* JETLAG (Tal-JET) deduced from the Trinity *de novo* transcriptome assembly, together with the top two tblastn species homologue sequences *Orussus abietinus* F-box/LRR repeat protein 20 (Oruab-FBOX/LRR; Accession No. XM_012430170) and *Harpegnathos saltator* F-box/LRR repeat protein 20 (Harsa-FBOX/LRR; Accession No. XM_011152298). '*' indicates identical amino acid residues in the two proteins, '.' and ':' indicate similar amino acid residues between the two proteins. In this figure SMART

identified domains consisting of one F-box domain and multiple leucine-rich repeat domains are highlighted in yellow and green respectively.
