# Supplementary material for: Identification and temporal expression of putative circadian clock transcripts in the amphipod crustacean Talitrus saltator
Source: PeerJ. 2016 Oct 5;4:e2555. doi: 10.7717/peerj.2555 (PMC5068443; doi:10.7717/peerj.2555)
Supplement: Figure S27 — Timecourse samples taken from behaviourally rhythmic animals held in DD yield rhythmic changes in Talper abundance with peak mRNA levels in early night. Talper expression is normalised to Talak and shown as mean expression +/- SEM (N = 4). Black and grey bars illustrate time of expected day and night, respectively. [file peerj-04-2555-s027.pdf]

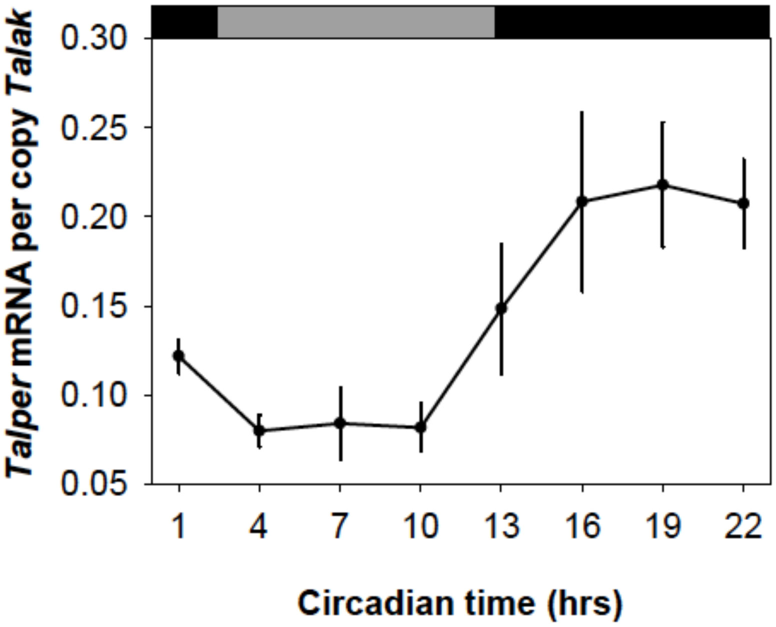

Figure S27. Expression profiling of *Talper* mRNA by Taqman PCR

Timecourse samples taken from behaviourally rhythmic animals held in DD yield rhythmic changes in *Talper* abundance with peak mRNA levels in early night. *Talper* expression is normalised to *Talak* and shown as mean expression +/- SEM (N=4). Black and grey bars illustrate time of expected day and night, respectively.
