# Supplementary material for: Identification and temporal expression of putative circadian clock transcripts in the amphipod crustacean Talitrus saltator
Source: PeerJ. 2016 Oct 5;4:e2555. doi: 10.7717/peerj.2555 (PMC5068443; doi:10.7717/peerj.2555)
Supplement: Table S1 [file peerj-04-2555-s028.docx]

**Supplementary Table S1. Primer, probes and adapter sequences used for *Talitrus saltator* degenerate Taqman and RACE PCR.** T7 refers to the phage promotor sequence added at the 5’ of the oligonucleotide; VIC**^®^** and NED**^®^** are probe fluors; MGB- Minor groove binding.

| Method | Oligonucleotide name | Sequence |
| --- | --- | --- |
| *Talitrus* Cry2 Degenerate PCR | Tal_Degen Cry2 F | GARGARYTIGGNTTYGAYAC |
|  | Tal_Degen Cry2 F Nested | GARMGIAARGCNTGGGTNGC |
|  | Tal_Degen Cry2 R | ATRWARTCICCRTTNGGRTC |
|  | Tal_Degen Cry2 R Nested | ACYTTCATNCCYTCYTCCCA |
| *Talitrus* Clk Degenerate PCR | Tal_Degen Clk F | TGGAARTTYYTNTTYYTNGAYCA |
|  | Tal_Degen Clk F Nested | AYCAYMGNGCNCCNCCANT |
|  | Tal_Degen Clk R | CCADATCCAYTGYTGNCCYTT |
|  | Tal_Degen Clk R Nested | TGNCCYTTNGTNARRAANCKRTA |
| *Talitrus* Cry2 3' RACE PCR | Tal_Cry2 3’ RACE F4 | GAGTTTTTCTACACGGCAGCCACCAACA |
|  | Tal_Cry2 3’ RACE F5 | ACGGCAGCCACCAACAACCCCAAA |
|  | Tal_Cry2 3’ RACE F6 | GACCACATGAAAGGCAATCCCATATG |
|  | Adapter Oligo(dT) | GACCACGCGTATCGATGTCGACTTTTTTTTTTTTTTTTV |
|  | Adapter Oligo(dT) primer | GACCACGCGTATCGATGTCGAC |
| *Talitrus* Clk 3' RACE PCR | Tal_Clk 3’ RACE F2 | GAGGGCCTCTGCAATCATCGGTTA |
|  | Tal_Clk 3’ RACE F3 | ATGTGGACGACCTGGAACGGGTGTCTAC |
| *Talitrus* PDH 3' RACE PCR | Tal_PDH 3’ RACE F1 | TGGGGCTGCCAAAGTTTTTGAGGGAG |
|  | Tal_PDH 3’ RACE F2 | GACAAGAGGAACTCGGAGCTCATCAACTC |
| *Talitrus* Cry2 5' RACE PCR | Tal_Cry2 5’ RACE R8 | CAGGCGAACAACGATTGTGGTGTCAT |
|  | Tal_Cry2 5’ RACE R9 | CCGAACGAGGCGACCCAGGCTTT |
|  | GeneRacer™ 5’ | CGACTGGAGCACGAGGACACTGA |
|  | GeneRacer™ 5’ Nested | GGACACTGACATGGACTGAAGGAGTA |
|  | Adapter GeneRacer™ RNA Oligo | CGACUGGAGCACGAGGACACUGACAUGGACUGAAGGAGUAGAAA |
| *Talitrus* PDH 5' RACE PCR | Tal_PDH 5’ RACE R1 | GAGTTGATGAGCTCCGAGTTCCTCTTGTC |
|  | Tal_PDH 5’ RACE R2 | CTCCCTCAAAAACTTTGGCAGCCCCA |
| *Talitrus* Taqman PCR | TalPerTaqF1 | GCCTCATGCCTCCATTCTACTG |
|  | TalPerTaqR1 | CGCAGAGGCTTGGTAGCATT |
|  | TalPerTaqProbe | VIC**^®^**-CGAGTTCGCGAGAAC |
|  | TalPerTaqStandF1 | CTCGAGATTCGGTGCATTTT |
|  | TalPerTaqStandR1T7 | T7-GCCATCACCTCTTCTTCCAA |
|  | TalAKTaqF1 | TCGAGGAACGGCTGTCCTT |
|  | TalAKTaqR1 | AGCAGCGTCAACCATTTTGTT |
|  | TalAKqProbe | NED**^®^**-CCTGAGCCAAGACAG-MGB |
|  | TalAKTaqStandF1 | TATTTGGGGGCACAATTTGA |
|  | TalAKTaqStandR1T7 | T7-CCAAGGCCAGTTTTCTTGTC |
