# Supplementary material for: Identification and temporal expression of putative circadian clock transcripts in the amphipod crustacean Talitrus saltator
Source: PeerJ. 2016 Oct 5;4:e2555. doi: 10.7717/peerj.2555 (PMC5068443; doi:10.7717/peerj.2555)
Supplement: Table S2 [file peerj-04-2555-s029.docx]

Supplementary Table S2. tblastn analyses of all *Talitrus saltator* circadian proteins vs. all NCBI curated non-redundant sequences

| Query | Top NCBI non-redundant sequence |  |  |  |
| --- | --- | --- | --- | --- |
|  | Accession no. | Species | E-value | % amino acid  identity/similarity |
| *Core clock proteins* | | | | |
| Tal-CRY2 | [KC885970](http://www.ncbi.nlm.nih.gov/nuccore/541906815) | *Eurydice pulchra* | 0.0 | 78/89 |
| Tal-CLK | [AY842303](http://www.ncbi.nlm.nih.gov/nuccore/61353790) | *Macrobrachium rosenbergii* | 3e-64 | 76/90 |
| Tal-PER | [KC885967](http://www.ncbi.nlm.nih.gov/nuccore/541906784) | *Eurydice pulchra* | 3e-121 | 44/64 |
| Tal-TIM | [XM_009048115](http://www.ncbi.nlm.nih.gov/nucleotide/676431782?report=genbank&log$=nuclalign&blast_rank=1&RID=S0RKY850016) | *Lottia gigantea* | 3e-108 | 49/68 |
| Tal-BMAL1 | [JQ670886](http://www.ncbi.nlm.nih.gov/nuccore/409711833) | *Pacifasticus leniusculus* | 4e-139 | 43/55 |
| *Clock associated proteins* | | | | |
| Tal-PDH I | [AB073368](http://www.ncbi.nlm.nih.gov/nuccore/20269278) | *Marsupenaeus japonicus* | 0.073 | 90/95 |
| Tal-PDH II | - | *-* | - | - |
| Tal-CK2α | [XM_011140473](http://www.ncbi.nlm.nih.gov/nucleotide/749752824?report=genbank&log$=nuclalign&blast_rank=1&RID=S0SHFB7V016) | *Harpegnathos saltator* | 0.0 | 92/97 |
| Tal-CK2β | [XM_012208415](http://www.ncbi.nlm.nih.gov/nucleotide/801375997?report=genbank&log$=nuclalign&blast_rank=1&RID=S0SN3J73016) | *Atta cephalotes* | 5e-124 | 82/90 |
| Tal-CWO | [XP_003744690](http://www.ncbi.nlm.nih.gov/protein/391340731?report=genbank&log$=prottop&blast_rank=1&RID=6MCU1T9W013) | *Metaseiulus occidentalis* | 8e-80 | 82/89 |
| Tal-DBT | [KC885972](http://www.ncbi.nlm.nih.gov/nuccore/541906832) | *Eurydice pulchra* | 0.0 | 95/97 |
| Tal-PDP1ε | [XM_012203776](http://www.ncbi.nlm.nih.gov/nucleotide/801397515?report=genbank&log$=nuclalign&blast_rank=1&RID=S0YKPYFS016) | *Atta cephalotes* | 2e-35 | 70/85 |
| Tal-PP1 | [XM_011065955](http://www.ncbi.nlm.nih.gov/nucleotide/746866386?report=genbank&log$=nuclalign&blast_rank=1&RID=S0Z8KE7A016) | *Acromyrmex echinatior* | 0.0 | 79/85 |
| Tal-MTS | [XM_002426681](http://www.ncbi.nlm.nih.gov/nuccore/242011989) | *Pediculus humanus corporis* | 0.0 | 95/98 |
| Tal-WBT | [XM_008197190](http://www.ncbi.nlm.nih.gov/nucleotide/642927805?report=genbank&log$=nuclalign&blast_rank=1&RID=S1014MMM013) | *Tribolium castaneum* | 0.0 | 88/94 |
| Tal-TWS | [JQ867383](http://www.ncbi.nlm.nih.gov/nuccore/391092522) | *Scylla paramamosain* | 0.0 | 86/92 |
| Tal-SGG | [XM_012400594](http://www.ncbi.nlm.nih.gov/nucleotide/817068181?report=genbank&log$=nuclalign&blast_rank=1&RID=S10R8F9P016) | *Athalia rosae* | 0.0 | 79/86 |
| Tal-SLIMB | [XM_012405697](http://www.ncbi.nlm.nih.gov/nucleotide/817053286?report=genbank&log$=nuclalign&blast_rank=1&RID=S10RK2WP016) | *Athalia rosae* | 0.0 | 82/90 |
| Tal-VRI | [JQ011276](http://www.ncbi.nlm.nih.gov/nucleotide/406507566?report=genbank&log$=nuclalign&blast_rank=1&RID=S10S4SRR016) | *Clunio marinus* | 6e-41 | 64/91 |
| Tal-EBONY | [XM_008199683](http://www.ncbi.nlm.nih.gov/nucleotide/642935141?report=genbank&log$=nuclalign&blast_rank=1&RID=S10SVXH7013) | *Tribolium castaneum* | 7e-85 | 39/56 |
| Tal-RORA | [XM_001987136](http://www.ncbi.nlm.nih.gov/nucleotide/195028615?report=genbank&log$=nuclalign&blast_rank=1&RID=T5PHTSME014) | *Drosophila grimshawi* | 3e-71 | 68/77 |
| Tal-REVERB | [AM710419](http://www.ncbi.nlm.nih.gov/nucleotide/187468481?report=genbank&log$=nuclalign&blast_rank=1&RID=T5RB1CBM015) | *Blattella germanica* | 2e-102 | 45/64 |
| Tal-SIRT1 | [XM_008555996](http://www.ncbi.nlm.nih.gov/nucleotide/665786802?report=genbank&log$=nuclalign&blast_rank=1&RID=T5SHZY5T015) | *Microplitis demolitor* | 2e-81 | 69/84 |
| Tal-SIRT2 | [XM_963962](http://www.ncbi.nlm.nih.gov/nucleotide/642928255?report=genbank&log$=nuclalign&blast_rank=1&RID=T5TETPM8014) | *Tribolium castaneum* | 5e-130 | 62/75 |
| Tal-SIRT4 | [XM_002605838](http://www.ncbi.nlm.nih.gov/nucleotide/260821124?report=genbank&log$=nuclalign&blast_rank=1&RID=T5YJJD2M015) | *Branchiostoma floridae* | 4e-110 | 57/69 |
| Tal-SIRT6 | [XM_011552750](http://www.ncbi.nlm.nih.gov/nucleotide/768420562?report=genbank&log$=nuclalign&blast_rank=1&RID=T5ZUJ41B015) | *Plutella xylostella* | 3e-120 | 55/71 |
| Tal-SIRT7 | [XM_012287821](http://www.ncbi.nlm.nih.gov/nucleotide/805796150?report=genbank&log$=nuclalign&blast_rank=1&RID=T60490GF014) | *Megachile rotundata* | 3e-144 | 56/71 |
| Tal-JET | [XM_012430170](http://www.ncbi.nlm.nih.gov/nucleotide/817219794?report=genbank&log$=nuclalign&blast_rank=1&RID=T60GMVKT014) | *Orussus abietinus* | 0.0 | 65/77 |
