# Supplementary material for: Identification and temporal expression of putative circadian clock transcripts in the amphipod crustacean Talitrus saltator
Source: PeerJ. 2016 Oct 5;4:e2555. doi: 10.7717/peerj.2555 (PMC5068443; doi:10.7717/peerj.2555)
Supplement: Table S3 [file peerj-04-2555-s030.docx]

Supplementary Table S3. blastp analyses of all *Talitrus saltator* circadian proteins vs. all NCBI curated non-redundant protein sequences

| Query | Top NCBI non-redundant sequence |  |  |  |
| --- | --- | --- | --- | --- |
|  | Accession no. | Species | E-value | % amino acid  identity/similarity |
| *Core clock proteins* | | | | |
| Tal-CRY2 | [AGV28717](http://www.ncbi.nlm.nih.gov/protein/541906816?report=genbank&log$=prottop&blast_rank=2&RID=6MATK5RN013) | *Eurydice pulchra* | 0.0 | 78/89 |
| Tal-CLK | [AAX44045](http://www.ncbi.nlm.nih.gov/protein/61353791) | *Macrobrachium rosenbergii* | 2e-67 | 71/84 |
| Tal-PER | [AGV28714](http://www.ncbi.nlm.nih.gov/protein/541906785?report=genbank&log$=prottop&blast_rank=1&RID=6MBGZCUU013) | *Eurydice pulchra* | 0.0 | 37/53 |
| Tal-TIM | [KDR17447](http://www.ncbi.nlm.nih.gov/protein/646712887?report=genbank&log$=prottop&blast_rank=1&RID=6MBPTE4Y015) | *Zootermopsis nevadensis* | 6e-139 | 52/72 |
| Tal-BMAL1 | [AFV39705](http://www.ncbi.nlm.nih.gov/protein/409711834?report=genbank&log$=prottop&blast_rank=1&RID=6MBZJDYA01R) | *Pacifasticus leniusculus* | 2e-154 | 43/57 |
| *Clock associated proteins* | | | | |
| Tal-PDH I | [BAB91011](http://www.ncbi.nlm.nih.gov/protein/20269279?report=genbank&log$=prottop&blast_rank=2&RID=6MC8CZ1N013) | *Marsupenaeus japonicus* | 0.041 | 90/95 |
| Tal-PDH II | [BAD13514](http://www.ncbi.nlm.nih.gov/protein/46090753?report=genbank&log$=prottop&blast_rank=1&RID=6MCBJ2SA013) | *Meimuna opalifera* | 0.091 | 34/48 |
| Tal-CK2α | [1NA7_A](http://www.ncbi.nlm.nih.gov/protein/34810211?report=genbank&log$=protalign&blast_rank=1&RID=S2HY4HSS01N) | *Homo sapiens* | 0.0 | 88/94 |
| Tal-CK2β | [XP_012287730](http://www.ncbi.nlm.nih.gov/protein/817181979?report=genbank&log$=protalign&blast_rank=1&RID=S2HYJ7Y2016) | *Orussus abietinus* | 8e-144 | 86/94 |
| Tal-CWO | [XP_003744690](http://www.ncbi.nlm.nih.gov/protein/391340731?report=genbank&log$=prottop&blast_rank=1&RID=6MCU1T9W013) | *Metaseiulus occidentalis* | 2e-80 | 82/89 |
| Tal-DBT | [AGV28719](http://www.ncbi.nlm.nih.gov/protein/541906833?report=genbank&log$=prottop&blast_rank=1&RID=6MD2G03W013) | *Eurydice pulchra* | 0.0 | 95/97 |
| Tal-PDP1ε | [EZA50108](http://www.ncbi.nlm.nih.gov/protein/607355530?report=genbank&log$=protalign&blast_rank=1&RID=S2JDZ25301N) | *Cerapachys biroi* | 7e-37 | 68/82 |
| Tal-PP1 | [XP_011136198](http://www.ncbi.nlm.nih.gov/protein/749748087?report=genbank&log$=protalign&blast_rank=1&RID=S2JEA21Z01N) | *Harpegnathos saltator* | 0.0 | 94/97 |
| Tal-MTS | [XP_002426726](http://www.ncbi.nlm.nih.gov/protein/242011990?report=genbank&log$=prottop&blast_rank=1&RID=6MDVCP96015) | *Pediculus humanus corporis* | 0.0 | 95/98 |
| Tal-WBT | [XP_971164](http://www.ncbi.nlm.nih.gov/protein/189238934?report=genbank&log$=prottop&blast_rank=1&RID=6ME39RXZ01R) | *Tribolium castaneum* | 0.0 | 88/94 |
| Tal-TWS | [AFK24473](http://www.ncbi.nlm.nih.gov/protein/388252859?report=genbank&log$=prottop&blast_rank=1&RID=6MED04EZ013) | *Scylla paramamosain* | 0.0 | 87/93 |
| Tal-SGG | [XP_012256017](http://www.ncbi.nlm.nih.gov/protein/817068182?report=genbank&log$=protalign&blast_rank=1&RID=S2K70DRR016) | *Athalia rosae* | 0.0 | 80/87 |
| Tal-SLIMB | [KDR19729](http://www.ncbi.nlm.nih.gov/protein/646716491?report=genbank&log$=prottop&blast_rank=1&RID=6MEH6R7K015) | *Zootermopsis nevadensis* | 0.0 | 84/92 |
| Tal-VRI | [KDR16467](http://www.ncbi.nlm.nih.gov/protein/646711216?report=genbank&log$=prottop&blast_rank=1&RID=6MEM59CT013) | *Zootermopsis nevadensis* | 3e-46 | 50/64 |
| Tal-EBONY | [CAI26307](http://www.ncbi.nlm.nih.gov/protein/71534702?report=genbank&log$=prottop&blast_rank=1&RID=6MER3AJ2013) | *Periplaneta americana* | 1e-95 | 39/57 |
| Tal-RORA | [XP_011290218](http://www.ncbi.nlm.nih.gov/protein/755858648?report=genbank&log$=protalign&blast_rank=1&RID=T5EXPTU201R) | *Musca domestica* | 2e-75 | 70/80 |
| Tal-REVERB | [XP_011259848](http://www.ncbi.nlm.nih.gov/protein/752884227?report=genbank&log$=protalign&blast_rank=1&RID=T5RB6UTA014) | *Camponotus floridanus* | 1e-105 | 48/65 |
| Tal-SIRT1 | [ABG78545](http://www.ncbi.nlm.nih.gov/protein/110613442?report=genbank&log$=protalign&blast_rank=1&RID=T5SJ7H0B015) | *Schistosoma mansoni* | 1e-115 | 47/62 |
| Tal-SIRT2 | [EFA06770](http://www.ncbi.nlm.nih.gov/protein/270010322?report=genbank&log$=protalign&blast_rank=1&RID=T5TF1G7U014) | *Tribolium castaneum* | 4e-141 | 60/73 |
| Tal-SIRT4 | [XP_008480918](http://www.ncbi.nlm.nih.gov/protein/662214193?report=genbank&log$=protalign&blast_rank=1&RID=T5YJU7ZF014) | *Diaphorina citri* | 8e-116 | 57/72 |
| Tal-SIRT6 | [EFX74386](http://www.ncbi.nlm.nih.gov/protein/321463370?report=genbank&log$=protalign&blast_rank=1&RID=T5ZUT70F015) | *Daphnia pulex* | 2e-123 | 50/67 |
| Tal-SIRT7 | [XP_012143211](http://www.ncbi.nlm.nih.gov/protein/805796151?report=genbank&log$=protalign&blast_rank=1&RID=T604D4KR014) | *Megachile rotundata* | 8e-147 | 56/71 |
| Tal-JET | [XP_008193983](http://www.ncbi.nlm.nih.gov/protein/642924044?report=genbank&log$=protalign&blast_rank=1&RID=T60GRNRG014) | *Tribolium castaneum* | 0.0 | 63/78 |
