# Supplementary material for: Identification and temporal expression of putative circadian clock transcripts in the amphipod crustacean Talitrus saltator
Source: PeerJ. 2016 Oct 5;4:e2555. doi: 10.7717/peerj.2555 (PMC5068443; doi:10.7717/peerj.2555)
Supplement: Table S4 [file peerj-04-2555-s031.docx]

Supplementary Table S4. blastp analyses of all *Talitrus saltator* circadian proteins vs. all FlyBase annotated protein sequences

| Query | Top FlyBase annotated protein |  |  |  |
| --- | --- | --- | --- | --- |
|  | FlyBase no. | Species | E-value | % amino acid  identity/similarity |
| *Core clock proteins* | | | | |
| Tal-CRY2 | - | *-* | - | - |
| Tal-CLK | [FBpp0306710](http://flybase.org/reports/FBpp0306710.html) | *Drosophila melanogaster* | 3e-56 | 56/77 |
| Tal-PER | [FBpp0304590](http://flybase.org/reports/FBpp0304590.html) | *Drosophila melanogaster* | 4e-50 | 29/50 |
| Tal-TIM | [FBpp0082180](http://flybase.org/reports/FBpp0082180.html) | *Drosophila melanogaster* | 4e-82 | 42/65 |
| Tal-BMAL1 | [FBpp0074693](http://flybase.org/reports/FBpp0074693.html) | *Drosophila melanogaster* | 1e-95 | 44/63 |
| *Clock associated proteins* | | | | |
| Tal-PDH I | [FBpp0084396](http://flybase.org/reports/FBpp0084396.html) | *Drosophila melanogaster* | 0.03 | 61/71 |
| Tal-PDH II | - | *-* | - | - |
| Tal-CK2 α | [FBpp0070041](http://flybase.org/reports/FBpp0070041.html) | *Drosophila melanogaster* | 7e-160 | 86/93 |
| Tal-CK2 β | [FBpp0300330](http://flybase.org/reports/FBpp0300330.html) | *Drosophila melanogaster* | 7e-109 | 85/91 |
| Tal-CWO | [FBpp0081723](http://flybase.org/reports/FBpp0081723.html) | *Drosophila melanogaster* | 4e-30 | 55/69 |
| Tal-DBT | [FBpp0306615](http://flybase.org/reports/FBpp0306615.html) | *Drosophila melanogaster* | 3e-154 | 82/90 |
| Tal-PDP1 ε | [FBpp0289727](http://flybase.org/reports/FBpp0289727.html) | *Drosophila melanogaster* | 1e-34 | 57/74 |
| Tal-PP1 | [FBpp0306442](http://flybase.org/reports/FBpp0306442.html) | *Drosophila melanogaster* | 3e-153 | 84/91 |
| Tal-MTS | [FBpp0310063](http://flybase.org/reports/FBpp0310063.html) | *Drosophila melanogaster* | 1e-174 | 92/97 |
| Tal-WBT | [FBpp0084575](http://flybase.org/reports/FBpp0084575.html) | *Drosophila melanogaster* | 0.0 | 86/92 |
| Tal-TWS | [FBpp0081671](http://flybase.org/reports/FBpp0081671.html) | *Drosophila melanogaster* | 0.0 | 80/91 |
| Tal-SGG | [FBpp0070450](http://flybase.org/reports/FBpp0070450.html) | *Drosophila melanogaster* | 0.0 | 79/88 |
| Tal-SLIMB | [FBpp0303082](http://flybase.org/reports/FBpp0303082.html) | *Drosophila melanogaster* | 0.0 | 78/88 |
| Tal-VRI | [FBpp0312171](http://flybase.org/reports/FBpp0312171.html) | *Drosophila melanogaster* | 5e-41 | 58/77 |
| Tal-EBONY | [FBpp0083505](http://flybase.org/reports/FBpp0083505.html) | *Drosophila melanogaster* | 1e-85 | 38/58 |
| Tal-RORA | [FBpp0297438](http://flybase.org/reports/FBpp0297438.html) | *Drosophila melanogaster* | 4e-68 | 69/78 |
| Tal-REVERB | [FBpp0297726](http://flybase.org/reports/FBpp0297726.html) | *Drosophila melanogaster* | 1e-92 | 45/65 |
| Tal-SIRT1 | [FBpp0080015](http://flybase.org/reports/FBpp0080015.html) | *Drosophila melanogaster* | 5e-80 | 60/78 |
| Tal-SIRT2 | [FBpp0310647](http://flybase.org/reports/FBpp0310647.html) | *Drosophila melanogaster* | 1e-100 | 58/72 |
| Tal-SIRT4 | [FBpp0070817](http://flybase.org/reports/FBpp0070817.html) | *Drosophila melanogaster* | 6e-84 | 53/70 |
| Tal-SIRT6 | [FBpp0293897](http://flybase.org/reports/FBpp0293897.html) | *Drosophila melanogaster* | 4e-99 | 56/72 |
| Tal-SIRT7 | [FBpp0084733](http://flybase.org/reports/FBpp0084733.html) | *Drosophila melanogaster* | 3e-115 | 56/71 |
| Tal-JET | [FBpp0111980](http://flybase.org/reports/FBpp0111980.html) | *Drosophila melanogaster* | 9e-140 | 59/76 |
