# Supplementary material for: Identification and temporal expression of putative circadian clock transcripts in the amphipod crustacean Talitrus saltator
Source: PeerJ. 2016 Oct 5;4:e2555. doi: 10.7717/peerj.2555 (PMC5068443; doi:10.7717/peerj.2555)
Supplement: File S1 [file peerj-04-2555-s032.docx]

Supplementary File S1- Supplementary Methods (degenerate and RACE PCR and qPCR)

*Degenerate PCR*

Messenger RNA was enriched from total RNA using Dynabeads**^®^** mRNA DIRECT**™** oligodT_25_ magnetic beads (Life Technologies, UK) according to manufacturer's instructions. This was then reverse transcribed using AMV-RT kit (Promega, UK) in the following 20μl reaction mix: 1μl AMVRT, 4μl 5X AMVRT Buffer, 1μl random hexamer primers, 2μl dNTPs, 1μl RNase Inhibitor, 6μl DEPC treated water and 5μl of mRNA. The reaction was incubated at 37°C for 1 h and subsequently terminated by heating to 95°C for 5 min. In order to amplify *TalCry2*, a10μl PCR reaction mix, including Amplitaq Gold**^®^** 2X PCR mix (Life Technologies, UK), 0.5μl 100µM each of Tal_Degen Cry2 F primer (see Supplementary Table 1 for all primer sequences) and Tal_Degen Cry2 R primer, 1μl cDNA and 8μl DEPC-treated water was subject to the following thermal cycling conditions: 95°C 4 min activation followed by 40 cycles of 94°C 30 sec, 42°C 45 sec, 72°C 45 sec with a final extension at 72°C for 7 min. One microliter of this product was then used in a second (nested) round of PCR using a mix of 10μl Bioline MyTaq™ Red 2X PCR mix (Bioline, UK), 0.5μl 10μM each Tal_Degen Cry2 F Nested primer, 0.5μl Tal_Degen Cry2 R Nested primer, and 8μl DEPC water. Cycling conditions were 95°C 4 min activation followed be 35 cycles 94°C 30 sec, 46°C 45 sec, 72°C 45 sec with a final extension at 72°C for 7 min. PCR amplicons were resolved and visualised on a 1.5 % agarose gel. Bands were excised, purified and cloned for sequencing as detailed below.

PCR procedures used to amplify TalClk were identical to those for TalCry2 except that TalClk primers were used (Table S1), and annealing temperatures differed as follows. For the first round PCR an annealing temperature of 40°C was used whilst second round (nested) PCR was done with an annealing temperature of 45°C.

*5’ and 3’ Rapid amplification of cDNA ends (RACE) PCR*

RACE PCR was done using GeneRacer**™** kit (Life Technologies, UK). mRNA extracted from 1 μg total RNA was reverse-transcribed with Superscript**™** III reverse transcriptase (Life Technologies, UK) at 50°C for 50 minutes and using the GeneRacer 3’ oligo (dT) adapter primer (see Table 1 for all adapter sequences). For 5’ RACE, mRNA was dephosphorylated, decapped, ligated to a 5’ RACE RNA oligo, and reverse-transcribed with Superscript III**™** and using random primers, according to the manufacturer’s instructions. After reverse transcription, templates were removed by incubation with RNase H (2 U, 15 minutes, 37°C). The reagents were heated to 65°C for 5 min then transferred to ice for 1 min. The cDNA was subsequently used as a starting template for nested RACE PCR. For 3’ RACE each of 3 PCR rounds was done in the following 20μl reactions: 10μl Bioline MyTaq™ Red 2X PCR Mix (Bioline, UK), 3’ RACE Primer, 0.5μl Gene Specific Primer (1st round - Tal_Cry2 3’ RACE F4, 2nd round Tal_Cry2 3’ RACE F5, 3rd round Tal_Cry2 3’ RACE F6), 8.5μl water and 0.5μl cDNA (each successive PCR round used the product from the preceding PCR round as template). For each round of PCR the cycling conditions were: 95°C 4 min activation followed by 40 cycles of 94°C 30 sec, 58°C 45 sec, 72°C 45 sec with a final extension at 72°C for 7 min. Amplicons were resolved and visualised on a 1.5% agarose gel and bands excised, purified and cloned for sequencing as detailed below. The same procedure was used for TalClk and TalPDH-I however only two rounds of PCR were used along with the appropriate primers (Tal_Clk 3’ RACE F2 for first round then Tal_Clk 3’ RACE F3 for second round for TalClk; Tal_PDH 3’ RACE F1 for first round then Tal_PDH 3’ RACE F2 for second round for TalPDH-I).

The 5’ ends of TalCry2 and TalClk were amplified in the following PCRs: 10μl Amplitaq Gold PCR 2X Mix (Applied Biosystems, UK), 1.2μl GeneRacer™ 5’ Primer, 0.4μl Gene Specific Primer (Tal_Cry2 5’ RACE R8), 8μl water and 0.4μl cDNA. Cycling conditions were; 95°C 5 min activation followed by 5 cycles 94°C 30 sec, 72°C 2 min, 5 cycles 94°C 30 sec, 70°C 2 min, 25 cycles 94°C 30 sec, 65°C 30 sec, 72°C 2 min with a final extension at 72°C for 7 min. The cDNA product was used to seed a second round (nested) PCR as follows. Ten microliters Bioline MyTaq™ Red 2X PCR Mix (Bioline, UK), 0.5μl GeneRacer™ 5’ Nested Primer, 0.5μl Gene Specific Primer (Tal_Cry2 5’ RACE R9), 8μl water and 1μl template from first round PCR. Cycling conditions were; 95°C 5 min activation followed be 40 cycles 94°C 30 sec, 65°C 30 sec, 72°C 2 min with a final extension at 72°C for 10 min. Amplicons were resolved and visualised on a 1.5% agarose gel before bands were excised, purified and cloned into TOPO PCR4 vector, grown in TOP10’ E. coli and prepared for sequencing as described elsewhere (see Wilcockson and Webster 2008). An identical strategy was used for TalPDH-I using the appropriate primers (Tal_PDH 5’ RACE R1 for first round and Tal_PDH 5’ RACE R2 for the second round).

*Quantitative PCR*

We validated FPKM values for *Talper* since this showed the most robust pattern of cycling (see results section).

Quantitative RT-PCR was done using Applied Biosystems TaqMan^®^ MGB hydrolysis probes 5’ labeled with VIC (*Talper*) or NED (*TalAK*) as described previously [1]. Sequences TaqMan primers and probes are given in Table 1. RNA was extracted and reverse transcribed as detailed (Methods). Standard curves in the range 1 x10^11^ copies per μL for qPCR were made from cRNA produced by *in vitro* transcription using DNA templates amplified from brain cDNA and using T7 phage promoter-flanked PCR primers (Table 1). Quantitative RT-PCR (qRT-PCR) was done using Bioline Sensimix™ Probe mastermix containing the internal reference dye, ROX, according the manufacturers recommendations. Each 20 μl reaction contained 0.5 μL each primer (10 μM) and probe (2.5 μM) and 1 μL cDNA. *Talper* was measured in duplex reactions with the internal reference gene *arginine kinase* (*Talak, Contig #* 96331_c1_seq1). We investigated several candidate reference genes including *β-actin* and *α-tubulin* and found that only *Talak* provided reliable normalization in time series data. Quantitative PCRs were run in triplicate on an Applied Biosystems StepOne Plus machine. For each assay, standards in serial ten-fold dilutions were run in the range 10^9^-10^3^ copies per reaction. PCR efficiencies were in the range 90-100%. Data were expressed as copies *Talper* per copy of *Talak*. Time-course qPCR data were analysed by one-way ANOVA.

1. Sharp JH, Wilcockson DC, Webster SG: **Identification and expression of mRNAs encoding bursicon in the plesiomorphic central nervous system of Homarus gammarus**. *General and comparative endocrinology* 2010, **169**(1):65-74.
