# Supplementary material for: Identification and temporal expression of putative circadian clock transcripts in the amphipod crustacean Talitrus saltator
Source: PeerJ. 2016 Oct 5;4:e2555. doi: 10.7717/peerj.2555 (PMC5068443; doi:10.7717/peerj.2555)
Supplement: Figure S23 — Alignment of Drosophila melanogaster SIRT4 (Drome-SIRT4; Accession No. NP_572241) with the T. saltator SIRT4 (Tal-SIRT4) deduced from the Trinity de novo transcriptome assembly, together with the top two tblastn species homologue sequences Branchiostoma floridae hypothetical protein (Brafl-HYPO; Accession No. XM_002605838) and Diaphorina sitri SIRT4 (Diasi-SIRT4; Accession No. XM_008482696). ’*’ indicates identical amino acid residues in the two proteins, ’.’ and ’:’ indicate similar amino acid residues between the two proteins. In this figure one SMART identified SIR2 domain is highlighted in yellow. [file peerj-04-2555-s023.pdf]

```
Drome-SIRT4      --------------------------------MRVGQLLRFRSTSLRSSTARQ-------
Tal-SIRT4        MYILQKSCTCHIFFIIKQKPIAATVTRNVLLQPPTKSHFKFLSNLRTDSTIKCFRSNRHQ
Brafl-HYPO       --------------------------------MPVR--LQSNATTRVNTTANF-------
Diasi-SIRT4      -----------------------------MTTCRIQRLFFKHFIVPPDVTSRSI------
                                                       :        . * .

Drome-SIRT4      ----------EYVPHHKPVVEDDIKRLEDFLLSKPNVLVLTGAGISTESGIPDYRSEGVG
Tal-SIRT4        FLLSRFCNNSAFVPKHDPCSEELIEQLQAFVTRSNKIFVLTGAGISTESGIPDYRSEGVG
Brafl-HYPO       ----------HFVPVSDPADLQDVEELQDFVSTSKRLFVITGAGISTESGIPDYRSEGVG
Diasi-SIRT4      ----------SFIPKHKPVEESDINKLKQFIEKYNKILVVTGAGISTESGIPDYRSEGVG
                            ::*  .*   . :: *: *:    .::*:********************

Drome-SIRT4      LYARSNHKPVQHMEFVKSSAVRKRYWARNFVGWPKFSATQPNATHHALARFEREERVQAV
Tal-SIRT4        LYATSSKRPVQIKDFIAKAEVRQSYWARNYVGWPRFSAFEPNTTHHTLAEWEQKGKVAHI
Brafl-HYPO       LYARSDNRPVQYADFLKSGAIRQRYWARNYVGWPKFSSFSPNISHETLSGWESVGKLHWL
Diasi-SIRT4      LYARSDKRPVQFQDFLKSRRVRIRYWARNFVGWPRFSSFQPNANHYALKQMEDNEKLSYI
                 *** *.:.***  :*: .  :*  *****:****.**: .** .* :*   *   .:  :

Drome-SIRT4      VTQNVDRLHTKAGSRNVVEVHGSGYVVKCLSCEYRIDRHEFQSILASLNPAF-KDAPDMI
Tal-SIRT4        VTQNVDALHQKAGSIKVTELHGSAHVVQCMQCSHSLPRHALQPLLASLNPHLALPSSQLI
Brafl-HYPO       VTQNVDSLHIKAGSRKVTELHGSAARVMCLSCPSVIPRTDMQTRIKHLNPVW-HAESQEM
Diasi-SIRT4      ITQNVDGLHYKAGNKKVIEMHGTAFRVMCLGCDYEIDRHKFQKILEDLNPDL-MIESQEM
                 :***** ** ***. :* *:**:.  * *: *   : *  :*  :  ***      .: :

Drome-SIRT4      RPDGDVEIPLEYIENFRIPECTQCGGDLKPEIVFFGDSVPRPRVDQIAGMVYNSDGLLVL
Tal-SIRT4        RPDGDVELTQEAVSRFVVPPCPSCGGLLKPHVVFFGDNVPRARVEAVKEELRRCDALLAL
Brafl-HYPO       APDADVFLAPEQIAGFRVPECEKCGGILKPQIVFFGDNVPKPTVQFVHKMLEESDAMLVA
Diasi-SIRT4      RPDGDVEMSEETISKFHVPQCPHCHGDLKPDIVFFGDNIPRHRMEKIDHLVRSCDGVLVL
                  **.** :. * :  * :* *  * * *** :*****.:*.  :: :   :  .*.:*.

Drome-SIRT4      GSSLLVFSGYRVVLQTKDLKLPVGIVNIGETRADHLADIKISAKCGDVIPKLFDFRNSKS
Tal-SIRT4        GTSLQVYSAYRFILLASELGLPMAAVNIGTTRGDHLLQLRVPARCGLVLPLLHL------
Brafl-HYPO       GSSLQVYSAYRFVSAARDQKKPIAVLNIGPTRGDKLADLKVSARCGDVLPQIHL------
Diasi-SIRT4      GSSLTVYSGYRILLEATDLHLPIAIVNIGPTRGDSLAQLKVDAKCSQVLASLRKDQS---
                 *:** *:*.**.:  : :   *:. :*** **.* * ::.: *.*. *:. :

Drome-SIRT4      VS
Tal-SIRT4        --
Brafl-HYPO       --
Diasi-SIRT4      --
```

Additional file 23. Putative *Talitrus saltator* SIRT4 protein

Alignment of *Drosophila melanogaster* SIRT4 (Drome-SIRT4; Accession No. NP_572241) with the *T. saltator* SIRT4 (Tal-SIRT4) deduced from the Trinity *de novo* transcriptome assembly, together with the top two tblastn species homologue sequences *Branchiostoma floridae* hypothetical protein (Brafl-HYPO; Accession No. XM_002605838) and *Diaphorina sitri* SIRT4 (Diasi-SIRT4; Accession No. XM_008482696). '*' indicates identical amino acid residues in the two proteins, '.' and ':' indicate similar amino acid residues between the two proteins. In this figure one SMART identified SIR2 domain is highlighted in yellow.
